# Variation in the skulls of *Elgaria* and *Gerrhonotus* (Anguidae, Gerrhonotinae) and implications for phylogenetics and fossil identification

**DOI:** 10.7717/peerj.11602

**Published:** 2021-07-22

**Authors:** David T. Ledesma, Simon G. Scarpetta, Christopher J. Bell

**Affiliations:** 1 Department of Integrative Biology, The University of Texas at Austin, Austin, Texas, United States; 2 Department of Geological Sciences, Jackson School of Geosciences, The University of Texas at Austin, Austin, Texas, United States

**Keywords:** Skull, Variation, Lizard, Elgaria, Gerrhonotus, Osteology, Fossils, CT

## Abstract

**Background:**

There are limited data on intra- and interspecific osteological variation for many squamate clades. Those data are relevant for phylogenetic analyses that use osteological characters and for apomorphic identifications of fossils. We investigate whether morphological features in the skulls of extant gerrhonotine lizards can be used to distinguish taxa at the species- and genus-level and assess whether newly discovered intra- and interspecific osteological variation alters the utility of previously reported apomorphic features. We examined skulls of species belonging to the gerrhonotine genera *Elgaria* and *Gerrhonotus*. These genera contain 17 extant species, but the cranial osteology of only a few species was previously examined. As a result, intra- and interspecific osteological variation of these gerrhonotines is poorly understood.

**Methods:**

We employed high-resolution x-ray computed tomography (CT) to scan 25 alcohol-preserved specimens. We provide data on the skulls of all eight species of *Elgaria*, four for the first time, and five species of *Gerrhonotus*, three for the first time. We examined 3-D reconstructed skulls of the scanned specimens as well as dry, traditionally prepared skeletons (when they were available).

**Results:**

We found that the purported diagnostic utility of many previously described morphological features is impacted because of substantial morphological variation between and within species. We present an assessment of osteological differences that may be useful to differentiate species of *Elgaria* and *Gerrhonotus*, many of which are present on isolated cranial elements commonly recovered as fossils, including the premaxilla, maxilla, parietal, pterygoid, prootic, dentary, and surangular. We demonstrate the importance of documenting patterns of osteological variation using large sample sizes, and the utility of examining disarticulated cranial elements of the squamate skull to identify diagnostic morphology. This study adds to a growing body of literature suggesting that extensive documentation of morphological variation is needed to further our understanding of the phylogenetic and diagnostic utility of morphological features across vertebrate clades. Efforts in that direction likely will benefit from examination of disarticulated skeletal elements.

## Introduction

There is currently a paucity of data on patterns of intra- and interspecific osteological variation for many squamate clades (Evans, 2008). A firm understanding of patterns of variation in extant taxa aids in discovering and describing morphological features that are useful for identifying fossils (Bhullar, 2012; Pérez Ben, Gómez & Báez, 2014). An understanding of variation is also paramount for reconstructing phylogenetic relationships among extant and extinct taxa using osteological characters (e.g., Conrad, 2008; Bhullar, 2011; Gauthier et al., 2012) and for studies that use phylogenetic reconstructions based on osteological characters to inform taxonomy (e.g., Conrad et al., 2011). Analysis of variation aids in the repeatability and testability of phylogenetic hypothesis (Poe & Wiens, 2000), in that studies of osteological variation can assess the constancy of characters upon which phylogenetic analyses of osteological data are based (Joyce & Bell, 2004; Bever, 2009). Accordingly, new data on osteological variation can alter our understanding of the reliability of reported apomorphies or other morphological features that were used to diagnose and identify fossils (Bever, 2005), leading to reevaluations of previous biogeographic or evolutionary hypotheses based on data from the fossil record (e.g., Good, 1988a).

The dearth of knowledge on intra- and interspecific osteological variation in squamates in particular was partly attributed to the relatively minor emphasis placed on maintaining and growing modern skeletal collections (Bell & Mead, 2014). A robust sample size in the number and type of specimens (e.g., those that preserve data on size, sex, and geographic location of collection) and variety of sampled taxa is necessary to account for different types of variation, including ontogenetic and individual variation, bilateral asymmetry, polymorphism within monophyletic lineages, teratologies, pathologies, ecophenotypic plasticity, and sexual dimorphism (Jones & German, 2005). In recent years, there has been a growing body of literature dedicated to understanding patterns of osteological variation in squamates (e.g., Bell, Evans & Maisano, 2003; Nance, 2007; Bell, Mead & Swift, 2009; Čerňanský et al., 2019; Paparella et al., 2020; Takesh et al., 2020) many utilizing x-ray computed tomography (CT) methods (partially reviewed by Broeckhoven & du Plessis, 2018). The use of x-ray computed tomography to scan wet alcohol-preserved specimens has the potential to partially supplement the lack of traditionally prepared dry skeletons (Bell & Mead, 2014). CT is also useful for producing osteological data for species for which skeletal data are rare or difficult to obtain as is often the case for species known from only a few specimens or species that are now near extinction. Here, we utilize CT data to document morphology of the skulls of several species of gerrhonotine lizards.

Gerrhonotinae is a diverse clade of anguid lizard that contains over 50 species. Lizard species in this group are ecologically diverse and inhabit a large geographical area from British Columbia to Panama (Lamar et al., 2015; Leavitt et al., 2017). Gerrhonotine fossils are known from early Eocene deposits (Smith, 2009) and possibly from late Cretaceous sediments (Estes, 1964; Good, 1988a; Longrich, Bhullar & Gauthier, 2012). Crown gerrhonotines are known from at least the middle Miocene (Scarpetta, 2018). The osteology of the group was previously studied by several researchers (Cope, 1892; Tihen, 1949; McDowell & Bogert, 1954; Romer, 1956; Criley, 1968; Meszoely, 1970; Rieppel, 1980; Gauthier, 1982; Good, 1987; Good, 1988a); however, only a few species from each group within Gerrhonotinae were sampled. Variation in gerrhonotine osteology was previously reported in studies of ontogenetic variation (Good, 1995) and timing of fusion relative to sexual maturity (Maisano, 2002) in *Elgaria coerulea*, and an osteological description of the skull of *Elgaria panamintina* (Ledesma & Scarpetta, 2018). However, intra- and interspecific variation in other gerrhonotine species was not previously documented or was not described in detail. In this study we present variation in the skulls of the gerrhonotine lizard genera *Elgaria* and *Gerrhonotus*. We selected *Elgaria* and *Gerrhonotus* because the two clades were described as morphologically similar (Tihen, 1949), although the two genera are hypothesized to form a grade as opposed to a clade (Stebbins, 1958; Good, 1987; Pyron, Burbrink & Wiens, 2013; Zheng & Wiens, 2016).

Our sample includes all eight species of *Elgaria* and five species of *Gerrhonotus*, representing the most taxonomically extensive osteological dataset of these genera. We provide the first discussion of variation in the skulls of four species of *Elgaria* (*Elgaria cedrosensis*, *Elgaria nana*, *Elgaria paucicarinata*, and *Elgaria velazquezi*) and three species of *Gerrhonotus* (*Gerrhonotus lugoi*, *Gerrhonotus ophiurus*, and *Gerrhonotus parvus*). We discuss variation in previously reported diagnostic and apomorphic morphology, as well as variation in previously undescribed morphology. We comment on the phylogenetic and taxonomic implications of variation discovered in our sample, and the efficacy of those features for diagnosing taxa at the genus and species levels based on our new variation data.

## Methods

Institutional abbreviations are as follows: CAS, California Academy of Sciences, San Francisco, CA; CM, Carnegie Museum of Natural History, Pittsburgh, PA; MVZ, Museum of Vertebrate Zoology, Herpetology Collection, University of California, Berkeley, CA; LACM, Natural History Museum of Los Angeles County, CA; SDNHM, San Diego Natural History Museum, CA; SRSU, Sul Ross State University, Alpine, TX; TCWC, Biodiversity Research and Teaching Collections, Texas A&M University, College Station, TX; TNHC, Biodiversity Collections, Herpetology Collections (Texas Natural History Collections), The University of Texas at Austin, TX; TxVP, Texas Vertebrate Paleontology Collections, Jackson Museum of Earth History, The University of Texas at Austin, TX (formerly TMM); UF, University of Florida, Florida Museum of Natural History, Gainesville, FL; UTCT, The University of Texas High-Resolution X-ray Computed Tomography Facility, Austin, TX.

### Anatomical nomenclature

Anatomical nomenclature follows Evans (2008) and Ledesma & Scarpetta (2018) unless otherwise noted. In several cases, multiple names of an anatomical feature are given in parentheses to facilitate interpretation. Abbreviations for anatomical features that appear in figures can be found in the figure captions.

### CT sample and scanning information

Our sample includes both dry skeletal specimens and CT-scans of alcohol-preserved specimens (Table 1). Most specimens were relatively large individuals, but we also examined some smaller specimens of *Elgaria multicarinata* (TxVP M- 8578, TxVP M-8982), *Elgaria kingii* TxVP M- 8582, and *Gerrhonotus liocephalus* TCWC 9896. The heads of all alcohol-preserved specimens were scanned at the University of Texas High-Resolution CT Facility (UTCT) except for *E. kingii* UF 74645 (https://www.morphosource.org/Detail/MediaDetail/Show/media_id/24786) and *E. coerulea* UF 152969 (https://www.morphosource.org/Detail/MediaDetail/Show/media_id/24778) which were downloaded from http://www.MorphoSource.org. Most specimens were scanned individually, but in some cases, specimens were scanned together, including scans of the two *Elgaria multicarinata*, the two *Elgaria coerulea*, the two *Gerrhonotus infernalis*, *Gerrhonotus liocephalus* TCWC 9896 with *Gerrhonotus lugoi* CM 49012, and *Gerrhonotus ophiurus* TCWC 35604 with *Gerrhonotus liocephalus* TCWC 8585. CT scanning specifications for specimens scanned at the UTCT are provided (see Table 2). Isotropic voxel sizes for scanned specimens range from 9.62 μm to 25.8 μm. We examined at least two specimens of each species with the exception of *G. ophiurus*, for which only a single specimen could be acquired. All raw CT data for the image-processed skulls used in this study are available for download without restrictions at http://www.MorphoSource.org. All CT-scanned specimens were digitally reconstructed in Avizo 3D 8.1 or 9.1 software. The skulls were segmented (digitally disarticulated) into individual cranial elements in Avizo using the magic wand tool or manual selections. Gray-scale values used to make magic wand selections varied substantially among datasets and are not directly comparable between datasets, but bone gray-scale values largely fell into the range of 18,000–30,000. We did not segment separate cranial elements when two or more elements were largely fused to one another and/or there was no distinct boundary between the bones in the CT slices. Our evaluations of morphology were based on observations of both volume- and surface-renderings. All figures are surface renderings because surface renderings of segmented bones provide higher quality images. Care was taken to ensure that the surface rendering represent the true morphology of the bones; however, some thin bones (e.g., the septomaxilla) may have small holes as the result of the smoothing process in generating the surface models.

**Table 1:** Specimens of *Elgaria* and *Gerrhonotus* in our study and associated data. SVL (snout-vent-length) is in mm.

Specimen	Sex	SVL (mm)	Locality
*Elgaria cedrosensis* SDNHM 30296	?	83	Isla de Cedros, Baja California, Mexico
*Elgaria cedrosensis* SDNHM 27702	?	73	Isla de Cedros, Baja California, Mexico
*Elgaria coerulea* CAS 14509	?	?	San Francisco Co., CA
*Elgaria coerulea* UF 152969	?	?	Rogue River National Forest, Siskiyou Co., CA
*Elgaria coerulea* TxVP M-9008	Female	114	Humbolt Co., CA
*Elgaria coerulea* TxVP M-8977	?	98	Mendocino Co., CA
*Elgaria coerulea* TNHC 14643	?	94	Humboldt Co., CA
*Elgaria coerulea* TNHC 58792	?	94	Benton Co., CA
*Elgaria coerulea* TxVP M-8965	?	74	21.6 mi W of Castle Crags State Park, Trinity Co., CA
*Elgaria kingii* CAS 266265	?	?	N/A
*Elgaria kingii* UF 74645	?	?	Cochise Canyon, Rincon Mts, Exit 297 off Interstate 10, Mescel Road. Cochise Co., AZ
*Elgaria kingii* TxVP M-8981	?	96	Catalina Mts., Tucson Pima Co., AZ
*Elgaria kingii* SDNHM 27895	?	95	Coconino, AZ
*Elgaria kingii* SDNHM 24252	?	86	Mimbres near Water Canyon, NM
*Elgaria kingii* TxVP M-8582	?	75	N/A
*Elgaria multicarinata* CAS 54241	?	?	Santa Clara Co., CA
*Elgaria multicarinata* TxVP M-8990	?	?	Saddle Mountain, Clatsop Co., Oregon
*Elgaria multicarinata* TxVP M-8993	?	157	Riverside Co., CA
*Elgaria multicarinata* TxVP M-8975	Female	153	Riverside Co., CA
*Elgaria multicarinata* TxVP M-8991	?	153	Riverside Co., CA
*Elgaria multicarinata* TxVP M-9007	Female	143	Riverside Co., CA
*Elgaria multicarinata* TNHC 35666	?	127	Los Angeles, CA
*Elgaria multicarinata* TxVP M-8986	?	117	San Bernardino Co., CA
*Elgaria multicarinata* TxVP M-9005	Female	115	Riverside Co., CA
*Elgaria multicarinata* TxVP M-8992	?	112	San Bernardino Co., CA
*Elgaria multicarinata* TxVP M-9004	?	111	Alameda Co., CA
*Elgaria multicarinata* TxVP M-8974	?	107	Los Angeles Co., CA
*Elgaria multicarinata* TxVP M-8987	?	106	N/A
*Elgaria multicarinata* TxVP M-8988	?	105	Santa Barbara Co., CA
*Elgaria multicarinata* TNHC 4478	?	98	Los Angeles, CA
*Elgaria multicarinata* TxVP M-12129	?	93	Oregon
*Elgaria multicarinata* TxVP M-8980	?	91	San Bernardino Co., CA
*Elgaria multicarinata* TxVP M-8578	?	55	Riverside Co., CA
*Elgaria multicarinata* TxVP M-8982	?	46	Riverside Co., CA
*Elgaria nana* SDNHM 17102	?	100	Islas de Los Coronados North Island, Mexico
*Elgaria nana* SDNHM 52886	?	95	Islas de Los Coronados North Island, Mexico
*Elgaria panamintina* MVZ 191076	Male	119	Inyo Co., CA
*Elgaria panamintina* MVZ 75918	Male	113	Inyo Co., CA
*Elgaria paucicarinata* SDNHM 45106	?	102	La Laguna, Sierra de La Laguna, Baja California Sur, Mexico
*Elgaria paucicarinata* SDNHM 45100	?	101	La Laguna, Sierra de La Laguna, Baja California Sur, Mexico
*Elgaria velazquezi* SDNHM 68677	Male	120	La Cumbre de San Pedro, Baja California Sur, Mexico
*Elgaria velazquezi* SDNHM 68678	Male	103	41.5 km NW of Santa Rosalia, Baja California Sur, Mexico
*Gerrhonotus infernalis* TxVP M-7129	?	?	Travis Co., TX
*Gerrhonotus infernalis* TxVP M-1723	?	?	Travis Co., TX
*Gerrhonotus infernalis* TxVP M-11412	?	?	Central TX
*Gerrhonotus infernalis* TxVP M-13440	?	165	Austin, TX
*Gerrhonotus infernalis* TNHC 18988	?	157	Austin, TX
*Gerrhonotus infernalis* TxVP M-12353	Male	155	Austin, TX
*Gerrhonotus infernalis* TxVP M-13442	?	150	Austin, TX
*Gerrhonotus infernalis* TxVP M-11414	?	?	Central TX
*Gerrhonotus infernalis* TxVP M-11411	?	?	Brewster Co., TX
*Gerrhonotus infernalis* TNHC 92262	Male	176	Bamberger Ranch, Blanco Co., TX
*Gerrhonotus infernalis* TxVP M-13441	?	144	Austin, TX
*Gerrhonotus liocephalus* TCWC 8585	?	135	Acahuizotla, Guerrero, Mexico
*Gerrhonotus liocephalus* TCWC 9896	?	77	Acahuizotla, Guerrero, Mexico
*Gerrhonotus lugoi* LACM 116254	Female	84	Coahuila, Mexico
*Gerrhonotus lugoi* CM 49012	Female	79	11 km SW of Cuatro Cienegas de Carranza, Coahuila, Mexico
*Gerrhonotus ophiurus* TCWC 35604	?	114	33.8 mi W Valles, San Luis Potosí, Mexico
*Gerrhonotus parvus* SRSU 5537	Female	72	Nuevo Leon, Mexico 1 km S Galeana
*Gerrhonotus parvus* SRSU 5538	Female	55	Nuevo Leon, Mexico, 3 km SE Galeana

**Table 2:** CT scanned specimens in our study with scanning details.

Specimen	Scanner	Date scanned	Power of the X-ray beam	Number of Slices	Voxel Size
*Elgaria cedrosensis* SDNHM 30296	NSI scanner	3/28/16	150 kV, 0.2 mA	1,930	11.3 μm
*Elgaria cedrosensis* SDNHM 27702	NSI scanner	3/29/16	150 kV, 0.2 mA	1,954	11.8 μm
*Elgaria coerulea* TNHC 14643	NSI scanner	5/5/19	140 kV, 0.14 mA	1,830	13.4 μm
*Elgaria coerulea* TNHC 58792	NSI scanner	5/5/19	140 kV, 0.14 mA	1,830	13.4 μm
*Elgaria kingii* SDNHM 27895	NSI scanner	3/30/16	150 kV, 0.2 mA	1,922	12.9 μm
*Elgaria kingii* SDNHM 24252	NSI scanner	3/28/16	150 kV, 0.2 mA	1,948	14.3 μm
*Elgaria multicarinata* TNHC 35666	NSI scanner	3/28/17	150 kV, 0.2 mA	1,735	18.6 μm
*Elgaria multicarinata* TNHC 4478	NSI scanner	3/28/17	150 kV, 0.2 mA	1,735	18.6 μm
*Elgaria nana* SDNHM 17102	NSI scanner	3/21/16	150 kV, 0.2 mA	1,938	15.4 μm
*Elgaria nana* SDNHM 52886	NSI scanner	3/21/16	150 kV, 0.2 mA	1,927	15.4 μm
*Elgaria panamintina* MVZ 191076	NSI scanner	9/16/15	150 kV, 0.2 mA	1,771	18.1 μm
*Elgaria panamintina* MVZ 75918	NSI scanner	9/16/15	150 kV, 0.2 mA	1,774	18.1 μm
*Elgaria paucicarinata* SDNHM 45106	NSI scanner	3/23/16	150 kV, 0.2 mA	1,989	13.3 μm
*Elgaria paucicarinata* SDNHM 45100	NSI scanner	3/21/16	150 kV, 0.2 mA	1,938	14.3 μm
*Elgaria velazquezi* SDNHM 68677	NSI scanner	3/21/16	150 kV, 0.2 mA	1,967	18.1 μm
*Elgaria velazquezi* SDNHM 68678	NSI scanner	3/25/16	150 kV, 0.2 mA	1,957	16.1 μm
*Gerrhonotus infernalis* TNHC 18988	NSI scanner	3/28/17	150 kV, 0.2 mA	1,731	25.8 μm
*Gerrhonotus infernalis* TNHC 92262	NSI scanner	3/28/17	150 kV, 0.2 mA	1,731	25.8 μm
*Gerrhonotus liocephalus* TCWC 8585	NSI scanner	5/23/19	130 kV, 0.14 mA	1,842	17.9 μm
*Gerrhonotus liocephalus* TCWC 9896	NSI scanner	5/22/19	130 kV, 0.14 mA	1,861	11.7 μm
*Gerrhonotus lugoi* LACM 116254	NSI scanner	4/25/16	150 kV, 0.11 mA	1,989	11.8 μm
*Gerrhonotus lugoi* CM 49012	NSI scanner	5/22/19	130 kV, 0.14 mA	1,861	11.7 μm
*Gerrhonotus ophiurus* TCWC 35604	NSI scanner	5/23/19	130 kV, 0.14 mA	1,842	17.9 μm
*Gerrhonotus parvus* SRSU 5537	NSI scanner	6/16/16	120 kV, 0.17 mA	1,777	9.62 μm
*Gerrhonotus parvus* SRSU 5538	NSI scanner	6/10/16	120 kV, 0.17 mA	1,410	9.61 μm

Measurements of CT specimens were conducted in Avizo 3D 8.1 in orthographic view, and all figures are also in orthographic view. Shrinkage of dry skeletal specimens may create contact between bones (McDowell, 1967). Therefore, when bones had a small space between them (likely connected by a small amount of soft tissue) in CT specimens, we considered those bones to be in contact (i.e., features 1, 30, and 40). A largely immovable sutural contact was not required for bone contacts to be scored as present. Snout-vent-length (SVL) measurements for alcohol preserved specimens were taken from photographs of the specimens positioned next to a ruler with 1 mm subdivisions. We calibrated the images based on the ruler in the photographs in ImageJ and drew and measured a line starting from the snout along the middle of the body to the vent (see Table 1 for measurements).

### Taxonomic framework

The genus *Gerrhonotus* was first described by Wiegmann (1828), who accommodated six species within the genus, including *coeruleus* (now *Elgaria coerulea*), *deppei* (now *Abronia deppii*), *imbricatus* (now *Barisia imbricata*), *liocephalus* (*Gerrhonotus liocephalus*), *rudicollis* (now *Barisia rudicollis*), and *taeniatus* (now *Abronia taeniata*). The genus *Elgaria* was erected by Gray (1838), who assigned to it two species, *Elgaria kingii* and *E. multicarinata*. The species *coerulea* and 12 other forms, including subspecies, were placed in *Elgaria* by Tihen (1949), but all of those taxa were placed in the genus *Gerrhonotus* by Stebbins (1958). That proposal placed species previously assigned to *Elgaria* in the subgenus *Gerrhonotus*, but classified *E. coerulea* in the subgenus *Barisia* (Stebbins, 1958). External scale characters were used by Waddick & Smith (1974) to support the classification of Tihen (1949) in which *Elgaria* and *Gerrhonotus* are treated as distinct genera. Although Criley (1968) identified no features of the skull to distinguish gerrhonotine genera and support the classification of either Tihen (1949) or Stebbins (1958), Good (1987) identified features of the skull that permitted generic differentiation that largely supported the generic classification by Tihen (1949).

There are up to eight currently recognized species of *Elgaria* (Table 3). *Elgaria nana* was considered a distinct species by Grismer (2001) because the size at which *E. nana* reaches sexual maturity is smaller than that of *E. multicarinata*, but some recent authors considered *E*. *nana* to be conspecific with *E. multicarinata* (Feldman & Spicer, 2006; Leavitt et al., 2017). The taxonomy of *E. nana* requires further investigation, but for our analysis we maintained *E. nana* as a separate species. *Elgaria cedrosensis*, previously a subspecies of *E*. *paucicarinata* (Grismer, 1988), was elevated to species status by Grismer & Hollingsworth (2001). Genetic data revealed that *E. cedrosensis* likely only occurs on Isla Cedros (from where the specimens in our sample were collected) and not on the mainland Baja California peninsula (Leavitt et al., 2017). A novel phylogenetic hypothesis was recently proposed in which *E. panamintina* is nested within *Elgaria multicarinata* as currently circumscribed, and there are distinct northern and southern lineages of *E. multicarinata* (Leavitt et al., 2017). *Elgaria multicarinata* is currently considered a single species. The two distinct lineages are *E. multicarinata webbii* for populations in the south and *E. multicarinata multicarinata* for those in the north. Locality data are provided for specimens of *E. multicarinata* in our study except for TxVP M- 8987, for which no associated locality data are available.

**Table 3:** The constituent species of *Gerrhonotus* and *Elgaria* as recognized in this study.

*Gerrhonotus farri* Bryson & Graham, 2010^#^^+^	*Elgaria cedrosensis* (Fitch, 1934a)
*Gerrhonotus infernalis* Baird, 1858	*Elgaria coerulea* 1828 (Wiegmann, 1828)
*Gerrhonotus lazcanoi* Banda-Leal, Nevárez-de los Reyes & Bryson, 2017^#^^+^	*Elgaria kingii* 1838 Gray, 1838
*Gerrhonotus liocephalus* Wiegmann, 1828	*Elgaria multicarinata* (Blainville, 1835)*
*Gerrhonotus lugoi* McCoy, 1970	*Elgaria nana* (Fitch, 1934a)
*Gerrhonotus mccoyi* García-Vázquez et al., 2018b^#^^+^	*Elgaria panamintina* (Stebbins, 1958)
*Gerrhonotus ophiurus* Cope, 1867	*Elgaria paucicarinata* (Fitch, 1934b)
*Gerrhonotus parvus* Knight & Scudday, 1985^#^	*Elgaria velazquezi* Grismer & Hollingsworth, 2001
*Gerrhonotus rhombifer* (Peters, 1876)^#^^+^	

**Note:**

# Taxon known from only one or a few specimens.

+ Taxon not included in this study.

* *Elgaria multicarinata* which was recently hypothesized to include two distinct clades, a northern clade and a southern one.

Our sample includes specimens from both clades. *Elgaria nana* and *Elgaria panamintina* probably are nested within what is currently recognized as *Elgaria multicarinata* (Leavitt et al., 2017).

There are 10 currently recognized species of *Gerrhonotus* (including *Gerrhonotus* (=*Coloptychon*) *rhombifer*) (Table 3). *Gerrhonotus parvus* was described by Knight & Scudday (1985) but was subsequently assigned to *Elgaria* and was thought to be closely related to *E. kingii* based on external morphology (Smith, 1986; Good, 1988b). Phylogenetic analysis of molecular data suggested that *G. parvus* is most closely related to species in *Gerrhonotus* (Conroy et al., 2005; Leavitt et al., 2017). The monophyly of *Gerrhonotus* and the inclusion of both *G. parvus* and *G. lugoi* within *Gerrhonotus* was questioned in a more recent analysis (García-Vázquez et al., 2018a). Those authors found that the phylogenetic position of *G. parvus* as sister to all *Gerrhonotus*, excluding *G. lugoi*, was not strongly supported in all analyses. Additionally, *G. lugoi* was recovered with weak support as either sister to *Barisia* or as an early diverging member of *Gerrhonotus* (García-Vázquez et al., 2018a). *Gerrhonotus lugoi* was previously included in the genus *Barisia* (Waddick & Smith, 1974; Smith, 1986). In our study, we treated *G. lugoi* and *G. parvus* as separate taxa within the genus *Gerrhonotus* pending further investigation. A paraphyletic *Gerrhonotus infernalis* also was previously inferred (García-Vázquez et al., 2018a). All specimens of *G. infernalis* included in our study are from Texas. The genus *Mesaspis* was recently found to be paraphyletic with respect to *Abronia*, and it was suggested that species previously placed in *Mesaspis* should now be placed in *Abronia* (Gutiérrez-Rodríguez et al., 2021). We follow the suggestion of Gutiérrez-Rodríguez et al. (2021).

### Morphological matrix

We provide descriptions of all examined morphological features in our “Results” section below. We also provide a matrix (see Table S1) that contains scorings for features that were counts and features that we discretized into distinct states. However, this matrix as presented is not intended for phylogenetic analysis, but rather as a convenient and now-familiar way to summarize morphological data. We use the term ‘morphological feature’ to emphasize this distinction because the term ‘character’ is now almost inextricably associated with morphological features that are assessed specifically for their utility for phylogenetic analysis. Although we discuss the systematic significance of some features, the features we evaluated herein are not explicitly framed for that purpose. Instead, our overarching goal was to document and report variation and to assess the impact of variation on the reliability of previous statements made in the literature, especially about the potential utility of features in diagnosing *Elgaria* and *Gerrhonotus*.

Our work builds on the foundation laid by those who previously worked on these groups. They did so in the face of limited availability and sample sizes of skeletal specimens of many species, and without the benefit of digital technologies such as X-ray computed tomography. The limited taxonomic sampling reflected availability of specimens at the time the authors were writing, when the skeletal system of rare taxa could only be studied through destructive sampling, or at a minimum the removal of skin and removal or alteration of tissues surrounding the skeleton. For taxa known only from a type specimen or a small number of specimens (e.g., *Gerrhonotus rhombifer*; *Gerrhonotus parvus*) such destructive sampling was not possible. As our own work unfolded over the last several years, our taxon sampling became less complete when new species were described (e.g., *Gerrhonotus lazcanoi* in 2017, *Gerrhonotus mccoyi* in 2018). In addition to those two species previous authors also did not have access to *E. velazquezi*, named in 2001 and included in our sample, or *Gerrhonotus farri*, which was named in 2010, is known only from the type specimen (Meiri et al., 2018), and is not included in our study.

When we review statements made by previous authors, we do so with the understanding that those statements that addressed diagnostic features of particular genera or higher taxa are to be interpreted as statements that *apply to the species of those genera that were available for study at the time*. The same is true for our own work. Although we have expanded the taxon sampling relative to the samples available to our predecessors, our coverage is not complete, and our sample size remains low for many species. Our statements must, therefore, be interpreted by readers and subsequent authors as applying only to the specimens we examined (see Table 1). This general issue of interpreting the literature in its historical context is exacerbated by the fact that the taxonomic arrangement of species into more inclusive taxa such as genera also changes through time. Although the extant taxa included within *Elgaria* appear now to be stable, the same is not true for *Gerrhonotus*, the taxonomic makeup and monophyly of which are not yet well established (García-Vázquez et al., 2018a). For those reasons we made an effort here to indicate particular specimens and/or species to which our comments apply, especially for those currently placed in *Gerrhonotus*.

## Results

We found considerable morphological variation among specimens for most cranial elements. The following section is organized first by bone; with morphological features for a particular bone organized chronologically by publication. We discuss skeletal features that we found to vary or that were previously reported to vary among *Elgaria* or *Gerrhonotus* and also evaluated features for which variation was *not* previously addressed. Features that begin with a number indicate ones that we summarized in our matrix (‘scored’) in discrete states. Features that begin with a letter represent ones that we did not score.

There were several reasons that we did not score features, including non-independent morphology resulting in identical scorings for multiple features, our inability to identify or comprehend previous descriptions made by other researchers, and ambiguous or inconsistent scoring resulting from the way in which a previously described feature was constructed or described. We also recognized continuous variation in some features, making qualitative character states arbitrary and/or difficult to create and score.

### Premaxilla

**Figure 1:**
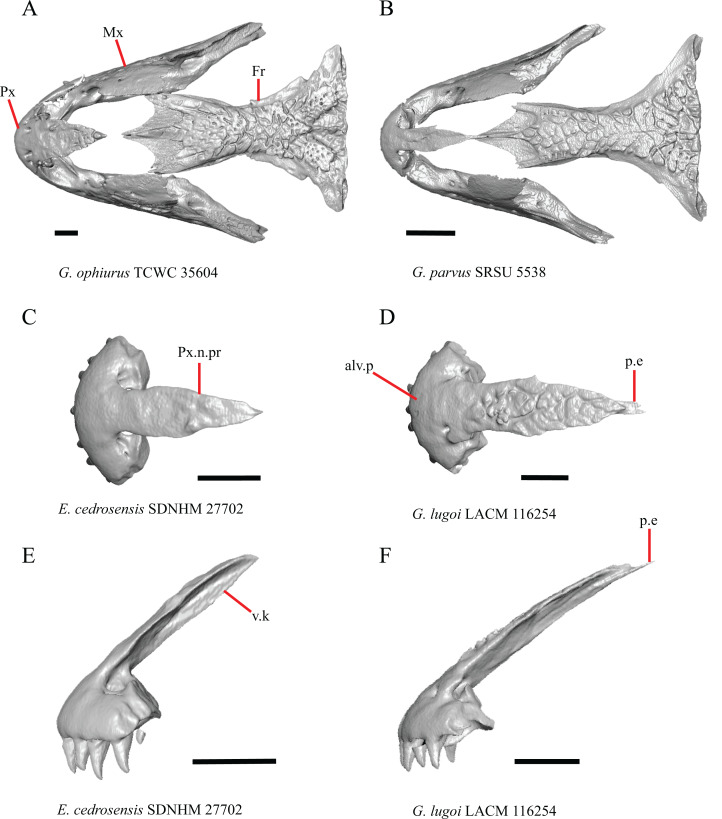
Premaxillae and some anterior skull bones of some species of *Elgaria* and *Gerrhonotus*. (A) Premaxilla, maxilla, and frontal of *G. ophiurus* TCWC 35604 in dorsal view. (B) Premaxilla, maxilla, and frontal of *G. parvus* SRSU 5538 in dorsal view. (C) Premaxilla of *E. cedrosensis* SDNHM 27702 in dorsal view. (D) Premaxilla of *G. lugoi* LACM 116254 in dorsal view. (E) Premaxilla of *E. cedrosensis* SDNHM 27702 in lateral view. (F) Premaxilla of *G. lugoi* LACM 116254 in lateral view. All scale bars equal 1 mm. alv.p, alveolar plate; Fr, frontal; Mx, maxilla; p.e, posterior extension; Px, premaxilla; Px.n.pr, premaxillary nasal process; v.k, ventral keel.

1. Contact between the premaxilla and the frontal with the nasals removed: 0=no contact, Fig. 1A; 1=contact, Fig. 1B (Tihen, 1949; modified from Good (1987), character 9).

Contact between the nasal process of the premaxilla and the frontal was reported in *Barisia*, *Abronia* (=*Mesaspis*) *gadovii*, and *Abronia* (Good, 1987). In these taxa, the nasal process of the premaxilla was reported to separate the nasals completely from one another. We found that when the nasals are removed, the nasal process of the premaxilla contacts the frontal in some specimens of *Gerrhonotus*, although the nasal process of the premaxilla does not separate the nasals completely from one another. It would be difficult to determine whether the premaxilla and frontal contact on a traditionally prepared skull in which the nasals overlie the anterior portion of the frontal and the posterior portion of the nasal process of the premaxilla. The nasal process and frontal do not contact in all *Gerrhonotus*, but in specimens that lack contact, the space separating the bones is relatively small. The premaxilla and frontal do not contact in specimens of *Elgaria*.

**Figure 2:**
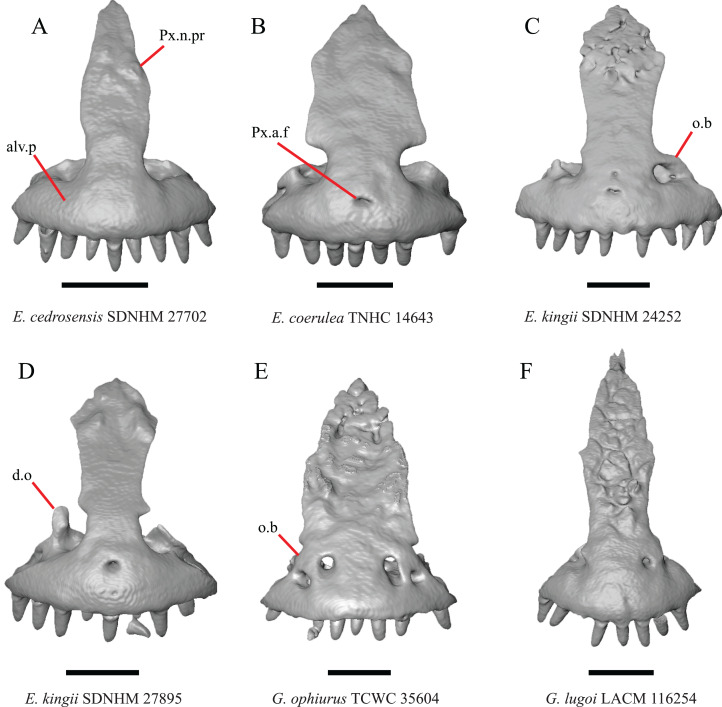
Premaxillae of some species of *Elgaria* and *Gerrhonotus*. (A) ****Premaxilla of *E. cedrosensis* SDNHM 27702 in anterior view. (B) Premaxilla of *E. coerulea* TNHC 14643 in anterior view. (C) Premaxilla of *E. kingii* SDNHM 24252 in anterior view. (D) Premaxilla of *E. kingii* SDNHM 27895 in anterior view. (E) Premaxilla of *G. ophiurus* TCWC 35604 in anterior view. (F) Premaxilla of *G. lugoi* LACM 116254 in anterior view. All scale bars equal 1 mm. alv.p, alveolar plate; d.o, dorsal ossification; o.b, ossified bridge; Px.a.f, anterior premaxillary foramen; Px.n.pr, premaxillary nasal process.

2. Lateral ossified connection between the nasal process and the alveolar plate of the premaxilla: 0=absent, Fig. 2A; 1=ossified projection(s) extend dorsally from the lateral portion of the alveolar plate of the premaxilla but do not connect to enclose the medial ethmoidal foramen, Fig. 2D; 2=ossified bridge encloses the medial ethmoidal foramen (foramen for ophthalmic branch of CN5 of Evans (2008)), Fig. 2E (modified from Good (1987), characters 1 and 2; Campbell & Frost, 1993; Scarpetta, 2018).

An ossified bridge was reported to occur in all gerrhonotines besides *Elgaria* and *Abronia* (Good, 1987). Ossified projections extending laterally from the nasal process but failing to connect with the alveolar plate were reported in *Abronia* (Good, 1987). We found that some *Elgaria* also possess ossified projections extending from the nasal process or have ossified projections that extend dorsally from the alveolar plate (e.g., *E*. *kingii* SDNHM 27895, Fig. 2D). We excluded the lateral projections from the discrete scoring of this feature due to continuous variation in distinctiveness of these projections. We scored a 1 if there were ossified projections extending dorsally from the lateral portion of the alveolar plate on one or both sides of the premaxilla, and we scored a 2 if there was a bridge on one or both sides of the premaxilla. Among *Elgaria*, a bridge is present on the left side of the premaxilla in *E*. *kingii* SDNHM 24252 (Fig. 2C), on the right side in *E*. *kingii* UF 74645 and *E*. *multicarinata* TxVP M- 8993, and on both sides of the premaxilla of *E*. *kingii* TxVP M- 8981. Asymmetry of the ossified bridge was also observed in *G. lugoi* LACM 116254 (Fig. 2F), which possesses the bridge on only the left side of the premaxilla. Specimens that have an ossified bridge on only one side of the premaxilla always possess non-connecting ossifications on the other side, supporting the homology of those features as was suggested by other authors (Campbell & Frost, 1993). Although an ossified bridge was reported to occur in *Gerrhonotus* (Good, 1987), both specimens of *G*. *parvus* and *G*. *lugoi* CM 49012 do not have a bridge nor ossified projections on either side of the premaxilla.

3. Midline foramen on the anterior surface of the alveolar plate of the premaxilla (anterior premaxillary foramen of Ledesma & Scarpetta, 2018): 0=absent, Fig. 2A; 1=present, Fig. 2B (Smith, 2009; Scarpetta, 2018).

We observed intraspecific variation in *E. kingii*, with one specimen (*E*. *kingii* CAS 266265) lacking a foramen, while all other specimens of that species have a foramen. A unique condition was observed in *E*. *kingii* SDNHM 24252, which has two foramina on the anterior surface (Fig. 2C). A foramen is absent from all specimens of *E*. *panamintina* and *E*. *cedrosensis*. All other species of *Elgaria* possess the foramen and most *Gerrhonotus* except for two specimens of *G*. *infernalis* (TxVP M- 11411, TxVP M- 13441) lack the foramen. However, in *G*. *infernalis* TxVP M- 11411, the foramen is minute and more ventrally located.

4. Number of premaxilla tooth positions (Conrad et al., 2011, character 406).

The presence of four bilateral tooth positions on the premaxilla was previously considered an unambiguous synapomorphy of Anguidae, including anguines, anniellines, gerrhonotines, and glyptosaurines (Conrad et al., 2011). However, we observed that same morphology in one specimen of the anguimorph *Xenosaurus grandis* (TxVP M- 8960), corroborating a finding by Bhullar (2011). Most specimens we examined possess four bilateral tooth positions and a central tooth position, for a total of nine premaxillary tooth positions. However, a few specimens (*E*. *panamintina* MVZ 75918, *E*. *multicarinata* TxVP M- 9004, and *E*. *coerulea* TNHC 14643) have eight tooth positions and several others (*E*. *kingii* SDNHM 24252, *E*. *multicarinata* TxVP M- 8988, and *G. infernalis* TxVP M- 13442) have ten tooth positions.

5. Morphology of the posterior end of the nasal process of the premaxilla: 0= posterior end tapers without a distinct posterior extension, Figs. 1C and 1E; 1=a thin posterior extension of the ventral keel of the premaxilla is present, Figs. 1D and 1F (new feature).

The shape of the posterior end of the nasal process of the premaxilla in specimens of *G. parvus* and *G*. *lugoi* is characterized by a thin extension of the ventral keel of the premaxilla that is not present on other specimens. All specimens that have a thin posterior extension of the premaxilla ventral keel also have the premaxilla and frontal in contact.

A. Width of the nasal process (Good, 1987, characters 7 and 8)

The nasal process was described by Good (1987) as parallel-sided between the nares in all gerrhonotines except for *Barisia*, in which it narrows posteriorly, and in *Gerrhonotus*, in which it narrows anteriorly in some specimens. We found intraspecific variation in the width of the nasal process in *Elgaria*, as did Good (1987). However, there were no specimens of *Gerrhonotus* that have a nasal process that significantly narrows anteriorly with the possible exception of *G*. *lugoi* LACM 116254 (Fig. 2F) and *G*. *parvus* SRSU 5538, which have a nasal process that is slightly widened midway along the process. However, several specimens of *Elgaria* also have a slight widening midway along the nasal process (e.g., *E. cedrosensis* SDNHM 27702, Fig 2A). The nasal process is widest relative to the anterior portion of the nasal process in *E*. *coerulea* TNHC 14643 (Fig. 2B), *E*. *coerulea* TNHC 58792, and *E*. *multicarinata* TNHC 35666. Interestingly, some specimens of *E*. *kingii* have a nasal process that is somewhat widened at the posterior end and narrows anteriorly (Fig. 2C); however, the nasal process is parallel-sided in *E*. *kingii* UF 74645 and is less distinctly widened at the posterior end in *E*. *kingii* TxVP M- 8981. We chose not to score this feature due to the continuous variation we observed among specimens, confounding creation of qualitative states.

### Maxilla

**Figure 3:**
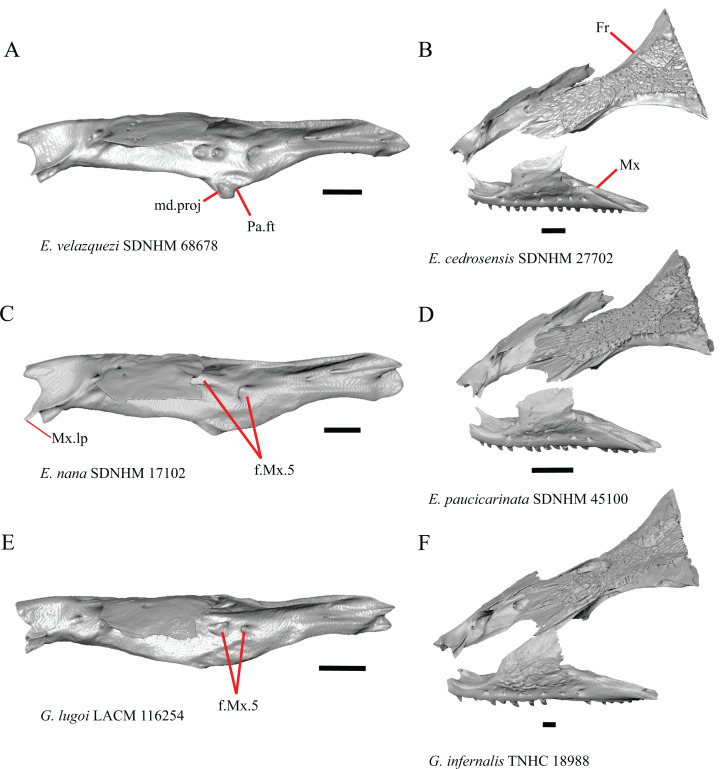
Maxillae and frontals of some species of *Elgaria* and *Gerrhonotus*. (A) Maxilla of *E. velazquezi* SDNHM 68678 in dorsal view. (B) Maxillae and frontal of *E. cedrosensis* SDNHM 27702 in dorsal view. (C) Maxilla of *E. nana* SDNHM 17102 in dorsal view. (D) Maxillae and frontal of *E. paucicarinata* SDNHM 45100 in dorsal view. (E) Maxilla of *G. lugoi* LACM 116254 in dorsal view. (F) Maxillae and frontal of *G. infernalis* TNHC 18988 in dorsal view. All scale bars equal 1 mm. Fr, frontal; f.Mx.5, maxillary trigeminal foramina; md.proj, medial projection; Mx, maxilla; Mx.lp, maxillary lappet; Pa.ft, palatine facet.

6. Contact between the maxilla and the frontal: 0=no contact, Fig. 3B; 1=contact, Fig. 3F (Tihen, 1949; Good, 1987, character 17).

Contact between the maxilla and the frontal was reported to occur in *Gerrhonotus* and *Abronia* (Good, 1987). We found contact between the maxilla and the frontal in specimens of *G. infernalis*, *G*. *ophiurus*, and *G*. *lugoi*, but not in specimens of *G*. *parvus* nor *G*. *liocephalus*. The maxilla and frontal do not contact in any specimens of *Elgaria*, but the maxilla comes closer to contacting the frontal in specimens of *E*. *paucicarinata* relative to other species of *Elgaria*. Absence of contact between the maxilla and frontal in *Elgaria* and *Barisia* was reported by Tihen (1949). Variation in this morphology was noted by Criley (1968), but he did not specify whether variation in maxilla-frontal contact was found in *Elgaria*, *Barisia*, or both genera.

7. Number of anterior openings of the superior alveolar canal at the base of anterior edge of the facial process (anterior inferior alveolar foramen of the maxilla of Oelrich, 1956; Smith, 2009) (Oelrich, 1956; Smith, 2009).

**Figure 4:**
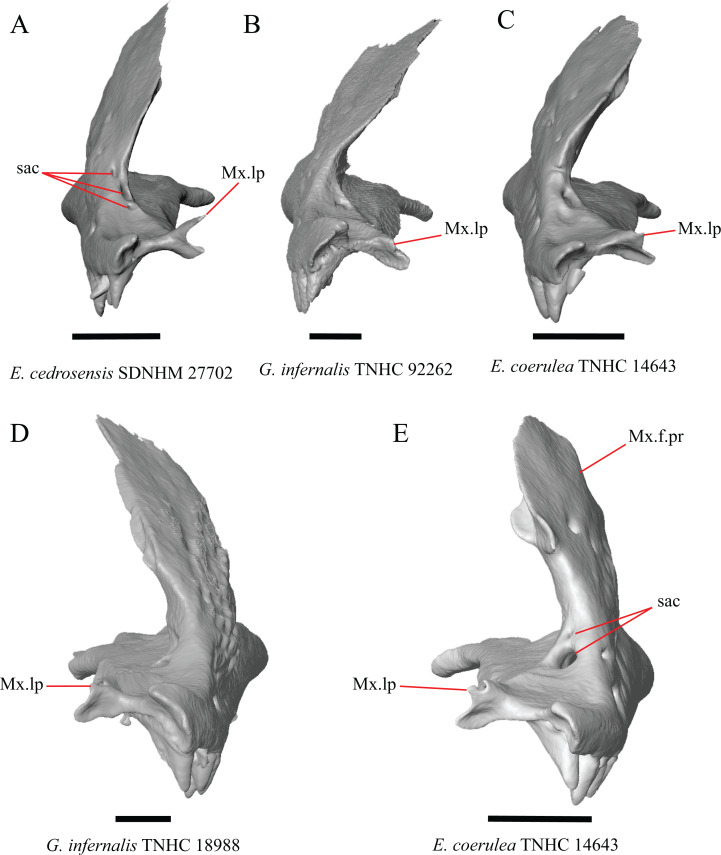
Maxillae of some species of *Elgaria* and *Gerrhonotus*. (A) Maxilla of *E. cedrosensis* SDNHM 27702 in anterior view. (B) Maxilla of *G. infernalis* TNHC 92262 in anterior view. (C) Maxilla of *E. coerulea* TNHC 14643 in anterior view. (D) Maxilla of *G. infernalis* SDNHM 18988 in anterior view. (E) Maxilla of *E. coerulea* TNHC 14643 in anterior view. All scale bars equal 1 mm. Mx.lp, maxillary lappet; Mx.f.pr, facial process of the maxilla; sac, opening of superior alveolar canal.

Several specimens of *Elgaria* and *Gerrhonotus* were found to have two openings for the superior alveolar canal and some were found to be bilaterally asymmetrical in the number. One specimen (*E. cedrosensis* SDNHM 27702, Fig. 4A) possesses three anterior openings for the superior alveolar canal on the right maxilla.

8. In a dorsal view, presence of a distinct medial projection at the anterior end of the palatine facet on the palatine process of the maxilla: 0=present, Fig. 3A; 1=absent, Fig. 3E (derived from Good, 1987, character 22)

*Gerrhonotus lugoi* is unique in our sample in that the maxillary shelf lacks a distinct medial projection where the maxilla articulates with the maxillary process of the palatine; however, the right maxilla of *G. lugoi* CM 49012 possesses a subtle projection. There is variation in the distinctiveness of a projection which ranges from being quite distinct (*Elgaria velazquezi* SDNHM 68678, Fig. 3A) to subtle (*E. nana* SDNHM 17102, Fig. 3C).

9. Number of maxillary tooth positions (Good, 1987, character 95).

A count of 21–24 maxillary tooth positions reportedly differentiates *Gerrhonotus* from other gerrhonotine genera, which were described as having 14 to 18 tooth positions (Good, 1987). We found that many specimens of *Elgaria* overlap with the count reported for *Gerrhonotus*. For example, *E. velazquezi* SDNHM 68677 has 23 maxillary tooth positions on each maxilla and *E. kingii* SDNHM 27895 has 21 tooth positions on the left maxilla and 22 on the right. Specimens of *G. parvus* have a maximum of 18 tooth positions and *G. lugoi* has a maximum of 19 tooth positions, both of which fall short of the number of tooth positions previously reported for *Gerrhonotus*. We hypothesize that the smaller adult body size of *G. parvus* and *G. lugoi* accounts for their reduced maxillary tooth position number relative to specimens of *G. infernalis* and large specimens of *Elgaria* that we examined. The number of teeth on the maxilla was shown to vary ontogenetically (indicated by head length) in *E. coerulea* (Good, 1995). The influence of body size on the number of teeth is corroborated by the fact that smaller specimens in our sample (e.g., *G. liocephalus* TCWC 9896) have fewer tooth positions than larger individuals of the same species. *Gerrhonotus infernalis* TxVP M- 7129 has the most tooth positions of any specimen with 26 tooth positions on the right maxilla and 25 on the left.

10. Number of labial nutrient foramina on the maxilla (Good, 1987).

**Figure 5:**
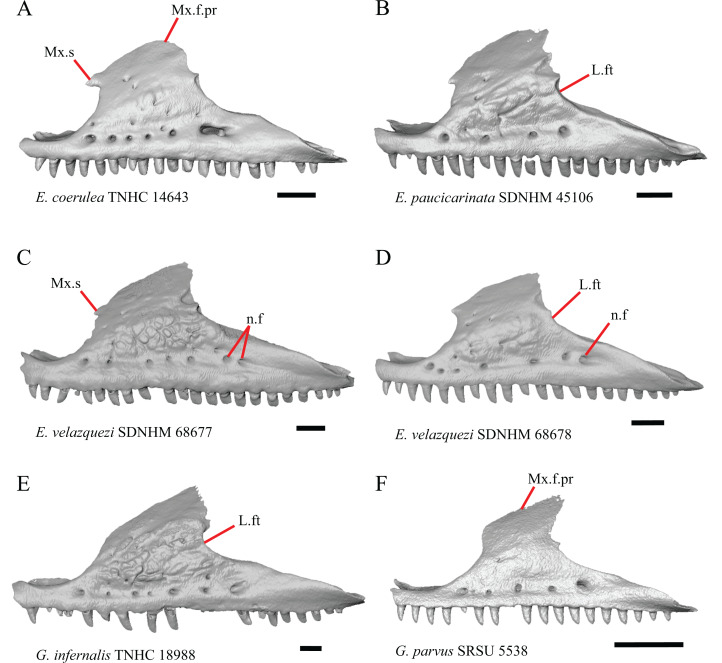
Maxillae of some species of *Elgaria* and *Gerrhonotus*. (A) Maxilla of *E. coerulea* TNHC 14643 in lateral view. (B) Maxilla of *E. paucicarinata* SDNHM 45106 in lateral view. (C) Maxilla of *E. velazquezi* SDNHM 68677 in lateral view. (D) Maxilla of *E. velazquezi* SDNHM 68678 in lateral view. (E) Maxilla of *G. infernalis* TNHC 18988 in lateral view. (F) Maxilla of *G. parvus* SRSU 5538 in lateral view. All scale bars equal 1 mm. L.ft, lacrimal facet; n.f, nutrient foramina; Mx.f.pr, facial process of the maxilla; Mx.s, maxilla spur.

The number of nutrient foramina on the maxilla varies intraspecifically among gerrhonotines, as was found previously (Good, 1987). In our sample, the number of foramina in a line running parallel to the tooth row ranges from four to eight. Additional foramina were occasionally present in variable positions on much of the lateral face of the maxilla (e.g., *E. velazquezi* SDNHM 68678, Fig. 5D). Multiple rows of foramina on the lateral surface of the facial process were observed in other anguimorphs (Bhullar, 2011).

11. Number of maxillary trigeminal foramina (Evans, 2008) (superior alveolar foramen of Smith (2009)) on the dorsal surface of the maxillary shelf (palatal shelf of Evans (2008)) and lateral to the palatine process (Smith, 2009).

The number of trigeminal foramina on the maxilla is two to three (Fig. 3A) with many specimens exhibiting bilateral asymmetry.

12. Spur on the anterior edge of the facial process of the maxilla: 0=absent, Fig. 5E; 1=present, Fig 5A (new feature).

Some specimens of *Elgaria* possess a spur on the anterior edge of the facial process, but specimens of *E. cedrosensis*, *E*. *coerulea* (except *E*. *coerulea* TNHC 14643), and *E. kingii* (except for *E. kingii* SDNHM 27895) lack a spur. The spur is variably present in *E. multicarinata* and *E. velazquezi* and presence may be bilaterally asymmetrical, such as in *E. multicarinata* TxVP M- 8992 and *E. velazquezi* SDNHM 67677. A spur is absent in all specimens of *Gerrhonotus* except for specimens of *G. liocephalus*, *G. lugoi*, G. *infernalis* TxVP M- 7129, and *G. ophiurus* TCWC 35604.

B. Shape of the overlap between the maxilla and the prefrontal (Good, 1987, character 16).

**Figure 6:**
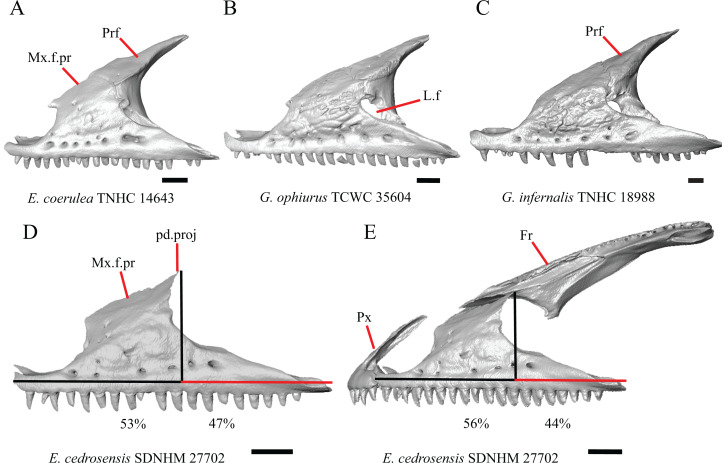
Maxillae, prefrontals, and frontal of some species of *Elgaria* and *Gerrhonotus*. (A) Maxilla and prefrontal of *E. coerulea* TNHC 14643 in lateral view. (B) ****Maxilla and prefrontal of *G. ophiurus* TCWC 35604 in lateral view. (C) Maxilla and prefrontal of *G. infernalis* TNHC 18988 in lateral view. (D) Maxilla of *E. cedrosensis* SDNHM 27702 in lateral view showing the location of the midpoint of the apex of the facial process relative to the total length of the maxilla. (E) Premaxilla, maxilla, and frontal of *E. cedrosensis* SDNHM 27702 in a view lateral to the skull but posterolateral to the maxilla showing the location of the midpoint of the apex of the facial process relative to the total length of the maxilla. All scale bars equal 1 mm. Fr, frontal; L.f, lacrimal foramen; pd.proj, posterodorsal projection; Prf, prefrontal; Mx.f.pr, facial process of the maxilla; Px, premaxilla.

The junction between the maxilla and the prefrontal was described as straight in *Gerrhonotus* (Fig. 6C), a lopsided ‘w’ shape in *Abronia*, or a ‘v’ shape (Fig. 6A) in all other gerrhonotines (Good, 1987). We did not score this feature because the ‘v’ shape contact can only be present in specimens which lack contact between the maxilla and the frontal (feature 6 of this study and character 17 of Good, 1987) (Figs. 6A and 6B).

C. Presence of sculpturing on the lateral surface of the maxilla (Conrad et al., 2011, character 8).

A rugose texture (dermal sculpturing) on the maxilla was reportedly absent in Anguidae, exclusive of *Diploglossus bilobatus* (Conrad et al., 2011). Additionally, dermal sculpturing was also reported on the maxilla of fossil *Diploglossus* from the Guadeloupe Islands (Bochaton et al., 2016a). We found some degree of sculpturing in most gerrhonotine specimens, albeit subtle in some specimens. There was continuous variation in the amount of sculpturing which prevented us from scoring this feature in discrete states, but dermal sculpturing is especially prominent in *E. multicarinata* (TNHC 35666, TxVP M- 8958, TxVP M- 8993), *E. velazquezi* SDNHM 67677 (Fig. 5C), and *G. infernalis* (TNHC 18988, TNHC 92262, TxVP M- 7129). Sculpturing on the maxilla was absent in most specimens of *E. coerulea*, two specimens of *E. kingii* (SDNHM 27895, CAS 266265), smaller specimens of *E. multicarinata* examined (TxVP M- 8980, TxVP M- 8578, TxVP M- 8982), and smaller specimens of *Gerrhonotus* (e.g., *G. parvus* SRSU 5538, Fig. 5F) suggesting that there is an ontogenetic component to the amount of sculpturing.

D. Location of the midpoint of the apex of the facial process of the maxilla (Conrad et al., 2011, character 28)

A midpoint of the apex of the facial process (nasal process of Conrad et al., 2011) located posterior to the longitudinal midpoint of the maxilla was reported as an unambiguous synapomorphy for *Elgaria* (Conrad et al., 2011). However, we found that the orientation in which we measured the maxilla affected whether the apex was anterior or posterior to the longitudinal midpoint of the maxilla. We measured the total length of the maxilla and the length from the anterior tip of the maxilla to the level of the midpoint of the apex of the facial process along a line parallel to the tooth row from a view directly lateral to the maxilla (Figs. 6D and 6E). With this method of measurement, the midpoint of the apex of the facial process is located just anterior to the longitudinal midpoint of the maxilla in both specimens of *E. velazquezi*, in *E. nana* SDNHM 52886, and in *E. multicarinata* TNHC 4478. In other specimens of *Elgaria* the midpoint of the apex of the facial process is located only slightly posterior to the longitudinal midpoint of the maxilla. The farthest posterior extent was seen in *E. panamintina* MVZ 191076 and *G. parvus* SRSU 5538, in which the midpoint/apex was located posteriorly at 55% of the total anterior-posterior length of the maxilla. We also measured the location of the midpoint of the apex of the facial process from a view lateral to the entire skull on several specimens so that the maxilla was oriented obliquely. This is the view that would likely be examined on an articulated, traditionally prepared skull. We found that the midpoint of the apex shifted about 2–3% more posteriorly with regard to the total anterior-posterior length of the maxilla. This is because the facial process is curved medially, making the location of the apex dependent on the orientation of the maxilla. We view this character as ambiguous, which results in inconsistent scoring. Because the location of the midpoint of the apex is always close to the longitudinal midpoint of the bone, having a midpoint of the apex that is just posterior to the midpoint is not a reliable diagnostic character of *Elgaria*. Furthermore, we found that some specimens of *Gerrhonotus* also have a midpoint of the apex of the facial process that is located posterior to the midpoint along the maxilla.

E. The inclination of the anterior edge of the facial process (Conrad et al., 2011, character 29).

The inclination of the anterior edge of the facial process, resulting from the relative degree of distinction between the ventral and posterior border of the naris, was used as a character in a large-scale phylogenetic analysis of squamates (Conrad et al., 2011). The condition reported for *Elgaria*, a weakly inclined anterior edge of the facial process (nasal process of Conrad et al., 2011), was recovered as an unambiguous synapomorphy of the genus (Conrad et al., 2011). However, we found that most of the specimens of *Elgaria* have an inclined anterior edge of the facial process that most closely resembles that of *Heloderma suspectum* as depicted by Conrad (2008, figure 26B), which was scored as having a steeply inclined facial process. We had difficulty scoring this character because of the ambiguity of the exemplar conditions provided by Conrad (2008) as well as the high degree of variation in the morphology of the anterior edge of the facial process. This resulted in specimens not easily being circumscribed into the different character states based on the character descriptions in their current form. *Elgaria velazquezi* SDNHM 68677, for example, has a morphology that is fully intermediate between a steep or shallowly inclined anterior edge of the facial process (Fig. 5C). *Gerrhonotus infernalis* TNHC 18988 (Fig. 5E) has a shallowly inclined anterior edge of the facial process while *G. infernalis* TNHC 92262 has a more distinct contrast between the anterior edge of the facial process and the dorsolateral edge of the premaxillary process. Specimens of *G. lugoi* and *G. parvus* have a more steeply sloped condition similar to that in most *Elgaria*. Like other authors (Simões et al., 2017), we were unable to score this character objectively, and do not recommend use in its current form in phylogenetic analyses of Gerrhonotinae.

F. Condition of the posterodorsal edge of the facial process of the maxilla from a lateral view (new feature).

There is significant variation in the shape of the posterior portion of the facial process among specimens of *Elgaria* and *Gerrhonotus*. In many specimens, the posterodorsal region of the facial process is rounded (e.g., *E*. *coerulea* TNHC 14643, Fig. 5A). The posterodorsal portion of the facial process is a broad posteriorly directed sheet in *G. parvus* SRSU 5538 (Fig. 5F). Other species have a distinct posterodorsal projection on the facial process. This projection is most distinct in specimens of *E. cedrosensis* (Fig. 6D), *E. nana* SDNHM 17102, and *G. infernalis* TxVP M- 13441. We did not score this feature in discrete states because the length of the posterodorsal projection is continuously variable and the distinctiveness may be contingent on the presence of notches on the posterior edge of the facial process (e.g., *E. paucicarinata* SDNHM 45106, Fig. 5B).

G. Notch in the posterior edge of the facial process of the maxilla where the lacrimal articulates (new feature).

Specimens of *G. infernalis*, except for *G. infernalis* TxVP M- 7129, have a notch in the posterior edge of facial process where the lacrimal articulates (Fig. 5E). This notch is also found in *G. ophiurus* TCWC 35604, on the right maxilla of *G. liocephalus* TCWC 8585, and a more subtle notch is present on the right maxilla of *E. kingii* TxVP M- 8981. Other specimens possess a small projection above the lacrimal articulation facet which creates a smaller notch (e.g., *E. paucicarinata* SDNHM 45106, Fig. 5B), have a thin lamina of bone where a notch would be present otherwise (e.g., *E. kingii* SDNHM 27895), or have no notch (e.g., *E. coerulea* TNHC 14643, Fig. 5A). We did not score this feature in distinct states because of continuous variation in the distinctiveness of a notch and because we found many ways in which a notch is formed, none of which are mutually exclusive.

H. Length of a medially projecting lappet on the maxilla (new feature).

There is significant variation in the morphology of a medially projecting lappet on the maxilla. The lappet ranges from being elongated (e.g., *E. cedrosensis* SDNHM 27702, Fig. 4A), short (e.g., *E*. *coerulea* TNHC 14643, Fig. 4C), or minute (e.g., *G. infernalis* TNHC 92262, Fig. 4B). We observed substantial variation in length of the lappet among specimens of both *Elgaria* and *Gerrhonotus*, but we note that the lappet is shortest in two specimens of *G. infernalis* (TNHC 18988, TNHC 92262) and *G. liocephalus* TCWC 9896. The lappet may also be incompletely or completely pierced by a foramen (e.g., left lappet of *G. infernalis* TNHC 18988, Fig. 4D, and the right lappet of *E*. *coerulea* TNHC 14643, Fig. 4E). We did not score this feature because a continuous spectrum of variation in length prevented us from reliably separating specimens into discrete qualitative states.

### Nasal

**Figure 7:**
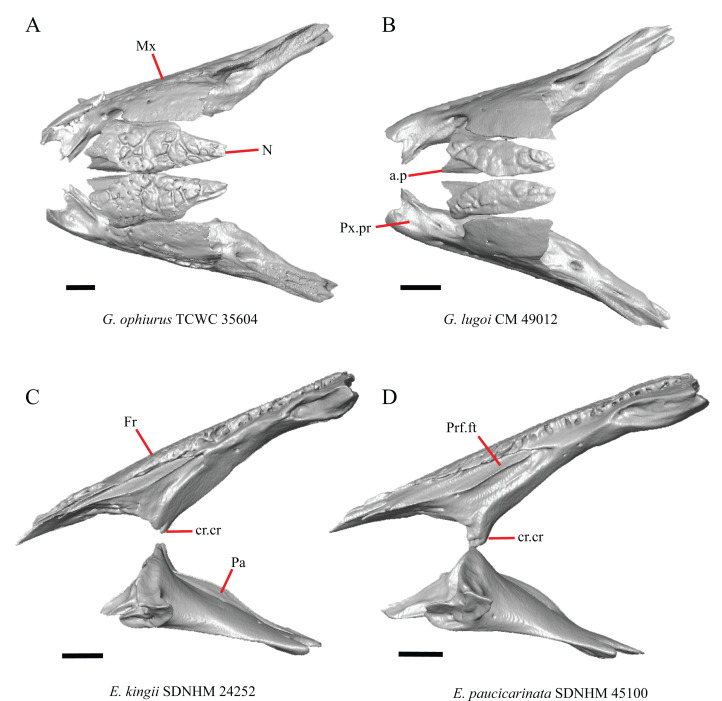
Maxillae, palatines, and some roofing bones of some species of *Elgaria* and *Gerrhonotus*. (A) Maxillae and nasals of *G. ophiurus* TCWC 35604 in dorsal view. (B) Maxillae and nasals of *G. lugoi* CM 49012 in dorsal view. (C) Frontal and palatines of *E. kingii* SDNHM 24252 in lateral view. (D) Frontal and palatines of *E. paucicarinata* SDNHM 45100 in lateral view. All scale bars equal 1 mm. a.p, anterior process; cr.cr, crista cranii; Fr, frontal; Mx, maxilla; N, nasal; Pa, palatine; Prf.ft, prefrontal facet; Px.pr, premaxillary process.

13. Closeness of the nasals at their anterior-posterior midpoint: 0=little to no separation at midpoint, Fig. 7A; 1=marked separation between the nasals near the midpoint, Fig. 7B (modified from Good, 1987, character 9).

Complete separation of the nasals from one another was reported in *Barisia*, *Abronia* (=*Mesaspis*) *gadovii*, and *Abronia* (Good, 1987). We observed a large separation between the nasals near their anterior-posterior midpoint only in specimens of *G. lugoi* (Fig. 7B).

14. Position of the anterior nasal process in dorsal view relative to the anteromedial inflection of the premaxillary process of the maxilla: 0=close to the anteromedial inflection of the premaxillary process of the maxilla, Fig. 7A; 1=far from the anteromedial inflection of the premaxillary process of the maxilla, Fig. 7B (similar to Gauthier et al. (2012), character 29).

Specimens of *G. lugoi* are unique in that the anterior process on the nasal is far posterior to the anteromedial inflection of the premaxillary process of the maxilla.

### Frontal

15. The position of the ventral tips of the crista cranii relative to the dorsal apex of the palatine: 0=the ventral tips of the crista cranii are dorsal to the palatine dorsal apex, Fig. 7C; 1=the ventral tips of the crista cranii extend ventral to or level to the dorsal apex of the palatine, Fig. 7D (modified from Conrad et al. (2011), character 67).

Contact between the cristae cranii and the palatines was reported as a synapomorphy of Anguidae (Conrad, 2008), as a synapomorphy of Gerrhonotinae + Diploglossinae (Gauthier, 1982), and as an unambiguous synapomorphy of gerrhonotines excluding *Elgaria* (Conrad et al., 2011). In the CT scans, the frontal and the palatine may not directly contact each other as they often do in dry skulls, so we modified this feature to instead describe the relative positions of the frontal and palatine. We found that the crista cranii extend ventrally below the dorsal apex of the palatine in many specimens of *Elgaria* and *Gerrhonotus* and some specimens were bilaterally asymmetric (e.g., *E. cedrosensis* SDNHM 27702). It was more recently claimed that this feature could not be scored qualitatively because of the continuous range of the ventral extent of the cristae cranii (Simões et al., 2017). A clear distinction can be made between the character states within our sample. Shrinkage caused by skeletal preparation of specimens may also influence the position of the crista cranii relative to the palatine. This would make comparisons between dry skeletal data and CT data problematic; however, the wide range in this morphology in both skeletal and CT specimens suggests that observed differences are not solely tied to specimen preparation.

I. Width of the interorbital region of the frontal (Estes, de Queiroz & Gauthier, 1988, character 7; Gauthier, 1982, characters 21 and 91; Conrad et al., 2011, character 58).

**Figure 8:**
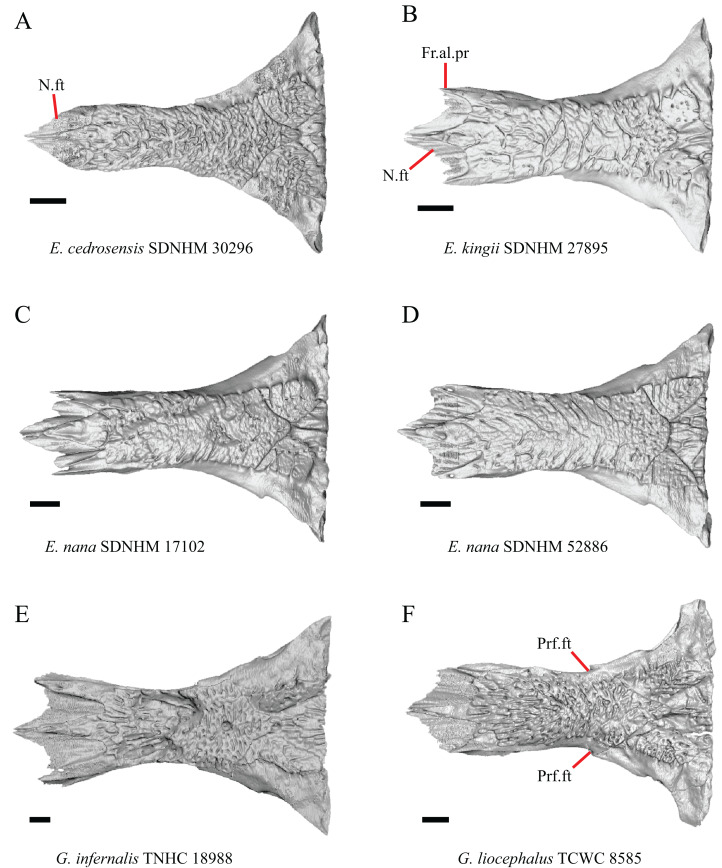
Frontals of some species of *Elgaria* and *Gerrhonotus*. (A) Frontal of *E. cedrosensis* SDNHM 30296 in dorsal view. (B) Frontal of *E. kingii* SDNHM 27895 in dorsal view. (C) Frontal of *E. nana* SDNHM 17102 in dorsal view. (D) Frontal of *E. nana* SDNHM 52886 in dorsal view. (E) Frontal of *G. infernalis* TNHC 18988 in dorsal view. (F) Frontal of *G. liocephalus* TCWC 8585 in dorsal view. All scale bars equal 1 mm. Fr.al.pr, anterolateral process of the frontal; N.ft, nasal facets; Prf.ft, prefrontal facet.

The frontal of gerrhonotine lizards was reported previously to have constricted interorbital margins (Meszoely, 1970; Estes, de Queiroz & Gauthier, 1988; Gauthier, 1982; Good, 1988a). Other authors reported linear and parallel interorbital margins in gerrhonotines (Conrad et al., 2011). These conflicting reports coincide with the variation discovered within our sample. We did not score specimens in discrete qualitative states because specimens exhibit a continuous range of variation from having an interorbital region that is distinctly narrower than the anterior region (e.g., specimens of *G. infernalis*, Fig. 8E), an interorbital region is only slightly narrower (e.g., *E. nana* SDNHM 52886, Fig. 8D), and an interorbital width is the same as the anterior width (e.g., *E. nana* SDNHM 17102, Fig. 8C). The width of the interorbital region reportedly varies ontogenetically in many lizards (Evans, 2008). In juvenile specimens of *Elgaria* the interorbital region appears constricted.

J. Condition of the anterolateral processes on the frontal (Evans, 2008).

The anterolateral processes on the frontal are relatively indistinct in *E. cedrosensis* SDNHM 30296 (Fig. 8A) and on the left side of *E. cedrosensis* SDNHM 27702. We did not score this feature in discrete qualitative states because there is a continuous range in the distinctiveness of those processes in our sample. It was previously noted that the processes may be variably developed in gerrhonotines (Evans, 2008).

K. Length of nasal facets of the frontal (new feature).

The nasal facets on the frontal in CT scans of *E. kingii* (SDNHM 28795, SDNHM 24252) appear somewhat shortened relative to the nasal facets of other specimens (Fig. 8B). However, there is not a clear distinction between short or long nasal facets in our sample and the shape and length of the nasal facet varies continuously within our sample.

L. Condition of the lateral edge of the frontal (new feature).

When viewed dorsally, the frontal of some specimens has a lateral margin with a notch in which the posterior tip of the orbital process of the prefrontal articulates (e.g., *G. liocephalus* TCWC 8585, Fig. 8F). In some specimens, the presence of a notch is bilaterally asymmetrical. We did not score this feature because the distinctiveness of a notch varies continuously and because co-ossified osteoderms may affect whether a notch is visible.

### Parietal

**Figure 9:**
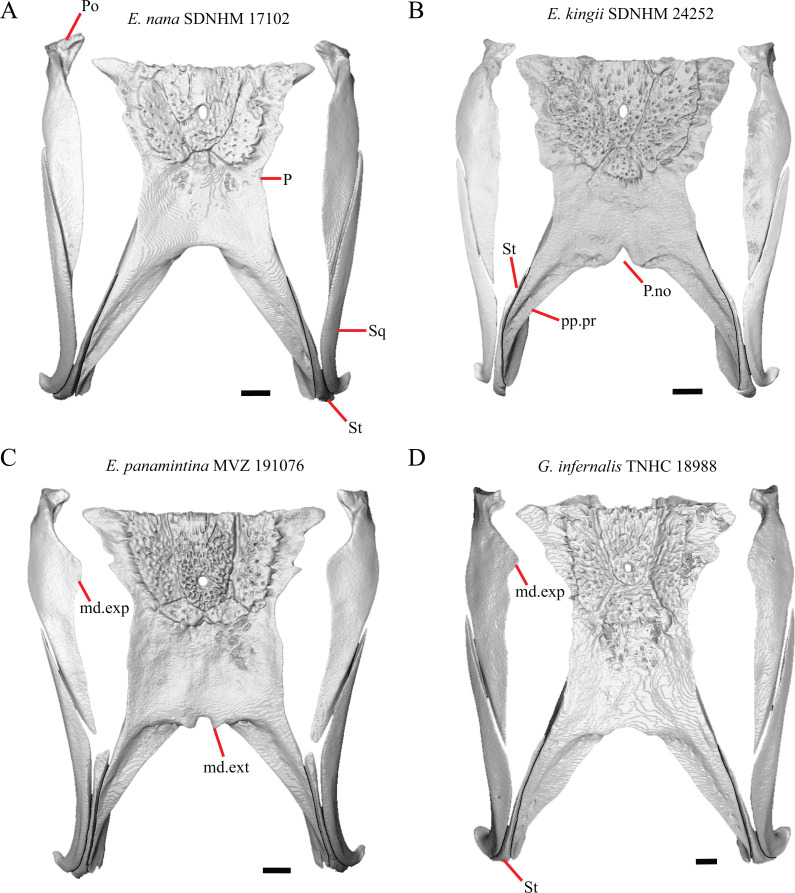
Parietals, supratemporals, and temporal bar bones of some species of *Elgaria* and *Gerrhonotus*. (A) ****Parietal, postorbitals, squamosals, and supratemporals of *E. nana* SDNHM 17102 in dorsal view. (B) Parietal, postorbitals, squamosals, and supratemporals of *E. kingii* SDNHM 24252 in dorsal view. (C) Parietal, postorbitals, squamosals, and supratemporals of *E. panamintina* MVZ 191076 in dorsal view. (D) Parietal, postorbitals, squamosals, and supratemporals of *G. infernalis* TNHC 18988 in dorsal view. All scale bars equal 1 mm. md.exp, medial expansion; md.ext, medial extension; P, parietal; P.no, parietal notch; Po, postorbital; pp.pr, postparietal process; Sq, squamosal; St, supratemporal.

16. The condition of the posterior edge of the parietal between the postparietal processes in dorsal or ventral view: 0=no notch, Fig. 9A; 1=notch present, Fig. 9B (Good, 1987, character 43).

**Figure 10:**
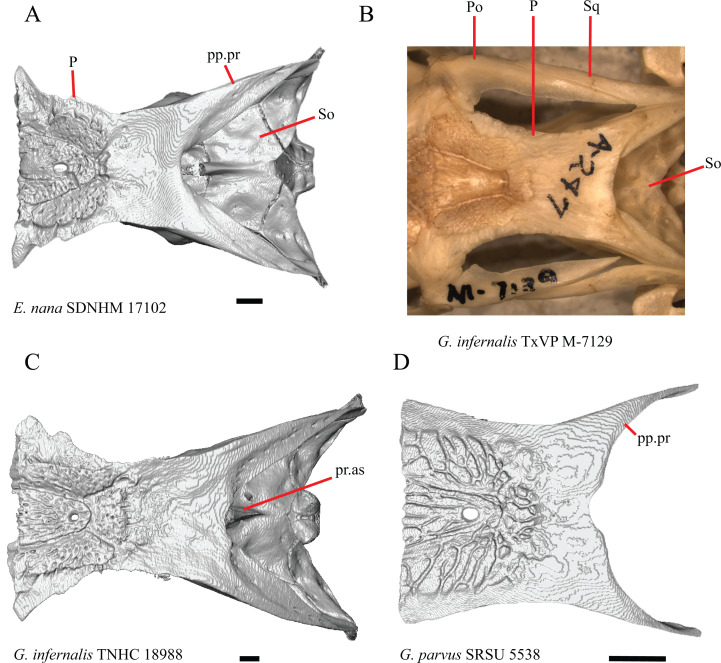
Parietals, braincases, and temporal bar bones of some species of *Elgaria* and *Gerrhonotus*. (A) Parietal and braincase of *E. nana* SDNHM 17102 in dorsal view. (B) Posterior portion of the skull of *G. infernalis* TxVP M-7129 in dorsal view. (C) Parietal and braincase of *G. infernalis* TNHC 18988 in dorsal view. (D) Parietal of *G. parvus* SRSU 5538 in dorsal view. All scale bars equal 1 mm. P, parietal; Po, postorbital; pp.pr, postparietal process; pr.as, processus ascendens; So, supraoccipital; Sq, squamosal.

A notched posterior edge of the parietal was reported for all gerrhonotines besides *Abronia deppii*, *Abronia oaxacae*, and *Abronia mixteca*, which were reported to have a rounded posterior edge (Good, 1987). We found that a notch is absent in many specimens of *Elgaria* (e.g., *E. nana* SDNHM 17102, Fig. 9A) and *Gerrhonotus* (e.g., *G. infernalis* TNHC 18988, Fig. 9D). When a notch is present it may be small and narrow (e.g., *E. kingii* SDNHM 24252, Fig. 9B) or large and rounded (e.g., *G. parvus* SRSU 5538, Fig. 10D). Additionally, we found that a notch may be present between posteriorly-facing medial extensions of the parietal (e.g., *E. panamintina* MVZ 191076, Fig. 9C) similar to that depicted by Evans (2008) in her figure 1.59 in showing a *Gerrhosaurus* sp.

17. Posterior extension of the parietal relative to the anteromedial end of the supraoccipital in dorsal view: 0=parietal does not overlap supraoccipital, Fig. 10A; 1=parietal overlaps the supraoccipital and obscures it from view dorsally, Fig. 10B (Good, 1987, character 42).

It was previously reported that the parietal extends posterior to the anterior end of the braincase in *Gerrhonotus* (Good, 1987). We interpreted this as the parietal extending posteriorly relative to the anterior end of the supraoccipital, because the parietal overlaps parts of the sphenoid and the alar process of the prootic in all specimens. We found that in most specimens of *Gerrhonotus* the parietal does not overlap the anterior end of the supraoccipital; only in some specimens of *G. infernalis* (TxVP M- 7129, TxVP M- 11411, TxVP M- 11412) is overlap present. In other specimens of *G. infernalis* (TNHC 18988, TxVP M- 7525, TxVP M- 12353) the parietal comes close to overlapping the anterior end of the supraoccipital (Fig. 10C), but that condition is similar to that observed in several specimens of *Elgaria*. The parietal does overlap the anterior end of the supraoccipital in two specimens of *E. multicarinata* (TxVP M- 8974, TxVP M- 8975). The extent to which the parietal is expanded posteriorly reportedly varies ontogenetically in lacertid lizards, with the parietal of juvenile specimens failing to overlap the braincase, while the parietal of adults covers the braincase (Barahona & Barbadillo, 1998).

**Figure 11:**
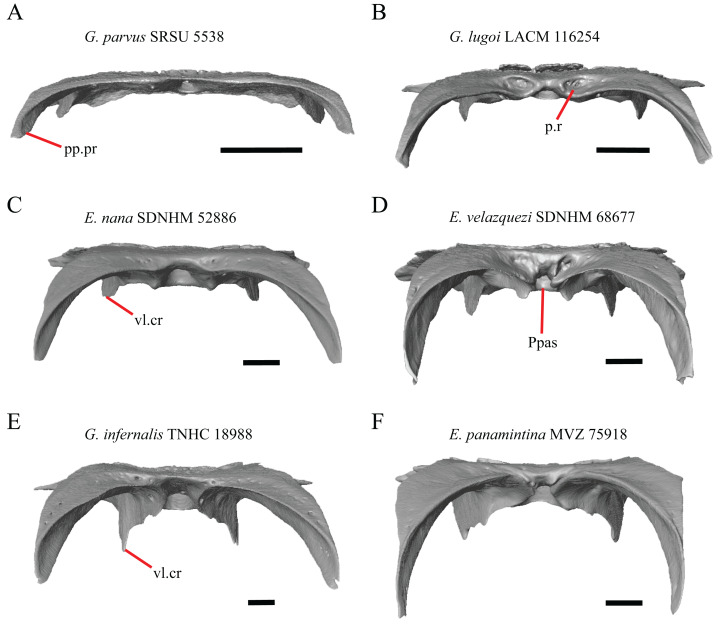
Parietals of some species of *Elgaria* and *Gerrhonotus*. (A) Parietal of *G. parvus* SRSU 5538 in posterior view. (B) Parietal of *G. lugoi* LACM 116254 in posterior view. (C) Parietal of *E. nana* SDNHM 52886 in posterior view. (D) Parietal of *E. velazquezi* SDNHM 68677 in posterior view. (E) Parietal of *G. infernalis* TNHC 18988 in posterior view. (F) Parietal of *E. panamintina* MVZ 75918 in posterior view. All scale bars equal 1 mm. Ppas, pit for the processus ascendens; pp.pr, postparietal process; p.r, posterior recess; vl.cr, ventrolateral crest.

18. Bilateral concave recess located on the posterior facing surface of the parietal between the postparietal processes: 0=absent or shallow, Fig. 11A; 1=present and deep, Fig. 11B (new feature).

A deep bilateral recess on the posterior edge of the parietal between the postparietal processes is present in specimens of *E. cedrosensis*, *E. velazquezi* SDNHM 68677 (Fig. 11D), *E*. *coerulea* CAS 14509, *G. liocephalus* TCWC 9896, *G. lugoi* LACM 116254 (Fig. 11B), and some specimens of *E. multicarinata* (TxVP M- 9007, although only on the right side in TxVP M- 8975). On the right side of *G. infernalis* TNHC 92296 there is a recess that is not defined ventrally like in other specimens. This feature may vary ontogenetically, because in *E. multicarinata* only specimens with a snout-vent-length over 140 mm have a deep recess.

M. Shape of the parietal table in dorsal view (Good, 1987, character 41).

The parietal table of *Abronia* (=*Mesaspis*) *moreletii* is reportedly broadened compared to its length (Good, 1987). Most specimens of *Elgaria* and *Gerrhonotus* have a parietal table that is trapezoidal in shape, but we found that some specimens have a parietal with anterolateral and posterolateral edges that are similar in lateral extent, giving the parietal a square-shaped appearance (e.g., *G. parvus* SRSU 5538, Fig. 10D). We did not assign discrete qualitative states to specimens because of a continuous spectrum of variation that may be due to ontogenetic variability. The shape of the parietal table was shown to vary ontogenetically in *E. multicarinata* (Bhullar, 2012), and juvenile specimens of *Elgaria* in our sample have a square-shaped parietal table.

N. Condition of the proximal medial edge of the postparietal processes (modified from Good, 1987, character 43).

The edges on either side of the posterior notch in the parietal were reported to “twist sharply downwards in *M*. [*Mesaspis*] *gadovii*…” (Good, 1987:289). Based on the description and illustrations of *Abronia* (=*Mesaspis*) *gadovii* provided by Good (1987) we interpret this as being the same as having a proximal medial edge of the postparietal processes that is steeply and ventromedially slanted. Specimens of *Elgaria* and *Gerrhonotus* exhibit a continuous range of morphological variation in the feature, including having a steeply slanted medial edge of the postparietal processes (e.g., specimens of *E. velazquezi* and *E. cedrosensis*, Fig. 11D) to having a flat medial edge (e.g., *G. parvus* SRSU 5538, Fig. 11A).

O. Border of the pit for the processus ascendens on the ventral surface of the parietal (Villa & Delfino, 2019).

The morphology of ridges that laterally border the pit for the processus ascendens on the ventral surface of the parietal varies intra- and interspecifically. Specimens exhibit a continuous range of variation in morphology of the ridge which ranges from being developed into a prominent crest that merge with the ventrolateral crests anteriorly (e.g., most specimens of *G. infernalis*, *E. panamintina* MVZ 75918, *E. velazquezi* SDNHM 68677, and *E. multicarinata* TNHC 35666) (Figs. 11E and 11F), absent (e.g., specimens of *G. parvus* and juvenile specimens of *Elgaria*) (Fig. 11A), or having an intermediate morphology (e.g., *E. nana* SDNHM 52886, Fig. 11C). The condition of the ridges appeared to vary with size, and larger specimens of *G. infernalis* possess a prominent crest while smaller species like *G. parvus* and *G. lugoi* do not have a ridge or have a ridge with only a minimal ventral extent.

### Prefrontal

**Figure 12:**
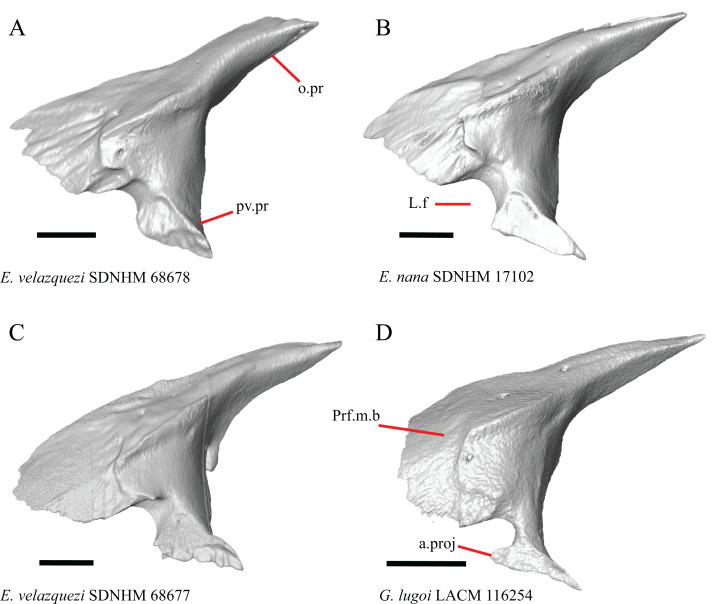
Prefrontals of some species of *Elgaria* and *Gerrhonotus*. (A) Prefrontal of *E. velazquezi* SDNHM 68678 in lateral view. (B) Prefrontal of *E. nana* SDNHM 17102 in lateral view. (C) Prefrontal of *E. velazquezi* SDNHM 68677 in lateral view. (D) Prefrontal of *G. lugoi* LACM 116254 in lateral view. All scale bars equal 1 mm. a.proj, anterior projection; L.f, lacrimal foramen; o.pr, orbital process; Prf.m.b, prefrontal main body; pv.pr, posteroventral process of the prefrontal.

19. Condition of the anterior edge of the posteroventral process of the prefrontal from a lateral view: 0= anterior projection absent, Figs. 12A and 12B; 1=anterior projection present, Fig. 12D (new feature).

Several specimens of *Elgaria* and *Gerrhonotus* have an anterior projection on the posteroventral process of the prefrontal. The process may extend far anteriorly (e.g., *G. lugoi* LACM 116254, Fig. 12D), or it may be bilaterally asymmetrical (e.g., *E. kingii* SDNHM 27895). The anterior edge of the posteroventral process on the left prefrontal of *E. velazquezi* SDNHM 68677 (Fig. 12C) has an anterior projection that is a small flange of bone compared to other *Elgaria* that have the anterior projection.

### Lacrimal

**Figure 13:**
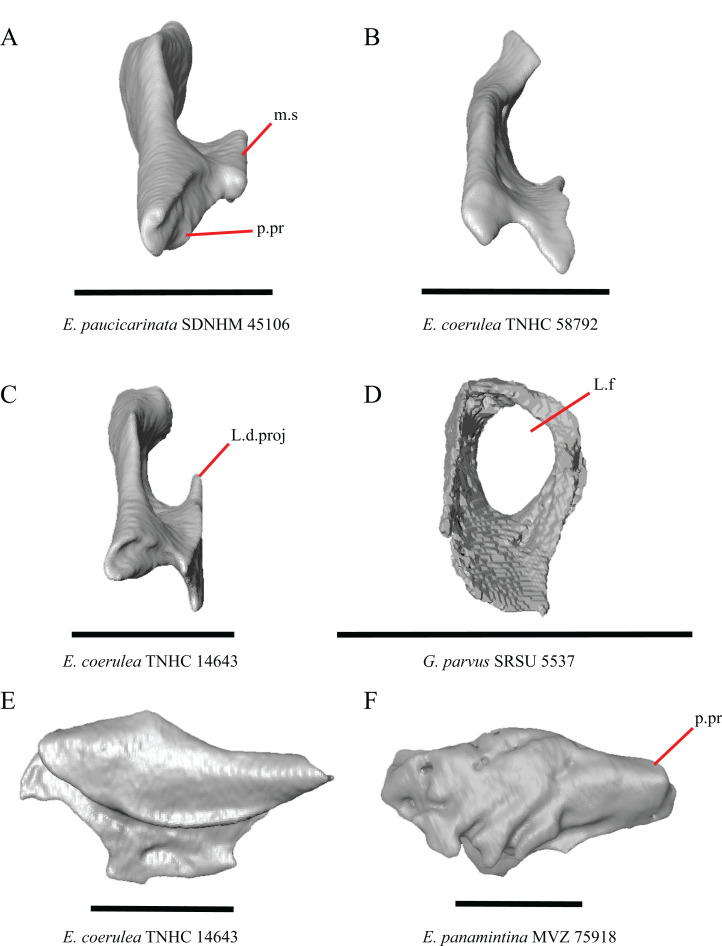
Lacrimals of some species of *Elgaria* and *Gerrhonotus*. (A) Lacrimal of *E. paucicarinata* SDNHM 45106 in posterior view. (B) Lacrimal of *E. coerulea* TNHC 58792 in posterior view. (C) Lacrimal of *E. coerulea* TNHC 14643 in posterior view. (D) Lacrimal of *G. parvus* SRSU 5537 in posterior view. (E) Lacrimal of *E. coerulea* TNHC 14643 in lateral view. (F) Lacrimal of *E. panamintina* MVZ 75918 in lateral view. All scale bars equal 1 mm. ****m.s, medial shelf; L.d.proj, lacrimal dorsal projection; L.f, lacrimal foramen; p.pr, posterior process. **** ****

20. Condition of a dorsal projection on the medial shelf of the lacrimal: 0=absent, Figs. 13A and 13B; 1=projection extends dorsally so that the lacrimal composes part of the medial border of the lacrimal foramen, Fig. 13C; 2=projection connects with the main body of the lacrimal so that the lacrimal fully encloses the lacrimal foramen, Fig. 13D (modified from Conrad et al., 2011, character 370).

A projection on the medial shelf of the lacrimal contributing to the medial border of the lacrimal foramen is absent in specimens of *E. paucicarinata* (Fig. 13A), *E. kingii* UF 74645, some specimens of *E. multicarinata* (TxVP M- 9005, TxVP M- 8990), *G. lugoi* LACM 116254, some specimens of *G. infernalis* (TxVP M- 13440, on the right side of TxVP M- 11411), and specimens of *G. liocephalus* and *G. ophiurus*. *Gerrhonotus parvus* SRSU 5537 is unique in possessing a lacrimal that fully encloses the lacrimal foramen (Fig. 13D).

21. Lateral sculpturing on the lacrimal: 0=absent, Fig. 13E; 1=present, Fig. 13F (new feature).

Lateral sculpturing (rugose texture) is present on the lacrimal of *E. panamintina* MVZ 75918 (Fig. 13F), *E. multicarinata* TxVP M- 8975, most specimens of *G. infernalis* (TNHC 18988, TxVP M- 7129, TxVP M- 1723, TxVP M- 11412, TxVP M- 11414, TxVP M- 11411), and *G. ophiurus* TCWC 35604.

**Figure 14:**
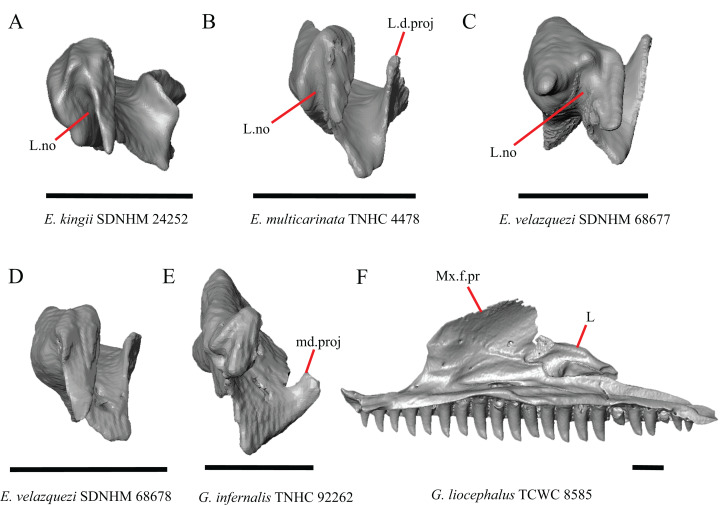
Lacrimals and maxilla of some species of *Elgaria* and *Gerrhonotus*. (A) Lacrimal of *E. kingii* SDNHM 24252 in anterior view. (B) Lacrimal of *E. multicarinata* TNHC 4478 in anterior view. (C) Lacrimal of *E. velazquezi* SDNHM 68677 in anterior view. (D) Lacrimal of *E. velazquezi* SDNHM 68678 in anterior view. (E) Lacrimal of *G. infernalis* TNHC 92262 in anterior view. (F) Maxilla and lacrimal of *G. liocephalus* TCWC 8585 in medial view. All scale bars equal 1 mm. ****L, lacrimal; L.d.proj, lacrimal dorsal projection; L.no, lacrimal notch; md.proj, medial projection; Mx.f.pr, facial process of the maxilla.

22. Condition of a medial projection on the medial shelf of the lacrimal that articulates with the anterior surface of the posteroventral process of the prefrontal; 0=absent, Fig. 14B;1=present, Fig. 14E (new feature).

In several specimens of *G. infernalis* there is a small medial projection at the anterior end of the medial shelf of the lacrimal that articulates with the anterior surface of the posteroventral process of the prefrontal. This feature is present but less distinct in *E. kingii* SDNHM 24252 (Fig. 14A).

P. Length of the posterior end of the lacrimal (new feature).

**Figure 15:**
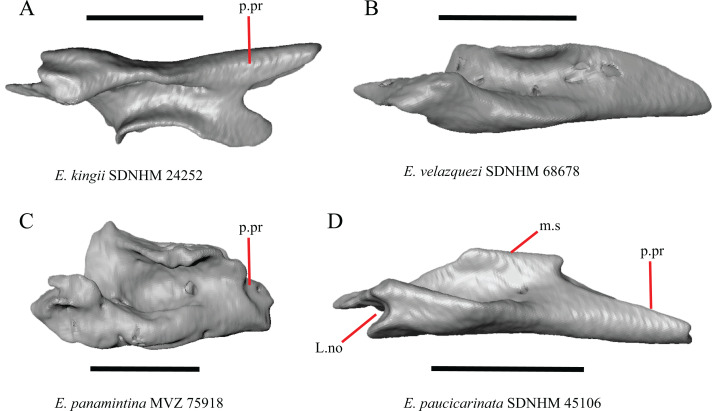
Lacrimals of some species of *Elgaria*. (A) ****Lacrimal of *E. kingii* SDNHM 24252 in dorsal view. (B) Lacrimal of *E. velazquezi* SDNHM 68678 in dorsal view. (C) Lacrimal of *E. panamintina* MVZ 75918 in dorsal view. (D) Lacrimal of *E. paucicarinata* SDNHM 45106 in dorsal view. All scale bars equal 1 mm. ****m.s, medial shelf; L.no, lacrimal notch; p.pr, posterior process.

There is substantial variation in the overall shape of the lacrimal. The posterior end of the lacrimal appears shortest in specimens of *E. panamintina* (Fig. 15C), *G. parvus*, *G. infernalis* TNHC 18988, and in some specimens of *E. multicarinata* (TNHC 35666, TxVP M- 9005, TxVP M- 8990). We observed continuous range in length and chose not to discretize this feature into distinct qualitative states.

Q. Condition of a notch between a posterior extension of the medial shelf of the lacrimal and the posterior process of the lacrimal (new feature).

A posterior projection extending from the medial shelf of the lacrimal creates a notch on the posterior end of the lacrimal in several specimens of *Elgaria* and *Gerrhonotus*. The distinctiveness of this notch ranges from being quite distinct (e.g., *E. kingii* SDNHM 24252, Fig. 15A), relatively indistinct (e.g., *E. paucicarinata* SDNHM 45106, Fig. 15D), completely absent (e.g., *E. velazquezi* SDNHM 68678, Fig. 15B), or bilaterally asymmetric (e.g., *E. multicarinata* TNHC 35666). We did not separate these morphologies into discrete qualitative states because we found continuous variation in the distinctiveness of a notch.

R. Condition of a notch on the anterior end of the lacrimal (new feature).

In some specimens of *Elgaria* and *Gerrhonotus* the anterior end of the lacrimal has a notch where the bone articulates with the maxilla. The morphology of the notch ranges from being distinct (Figs. 14A–14C and 15D), to less distinct (e.g., *E. multicarinata* TNHC 4478, Fig. 14B), to absent (e.g., *E. velazquezi* SDNHM 68678, Fig. 14D). In some specimens, the notch is indistinct but there is an elongate projection on the anterior end of the lacrimal that articulates with the medial surface of the maxilla (e.g., *G. liocephalus* TCWC 8585, Fig. 14F). We observed continuous range in the distinctiveness of a notch and chose not to discretize this feature into distinct qualitative states.

### Jugal

**Figure 16:**
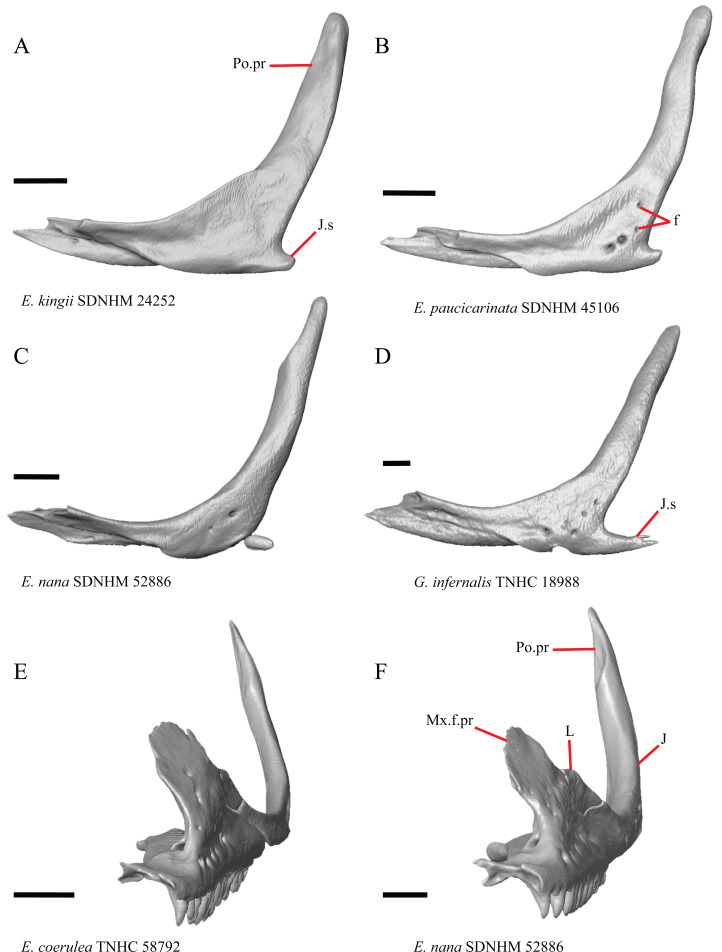
Lacrimals, jugals, and maxillae of some species of *Elgaria* and *Gerrhonotus*. (A) Jugal of *E. kingii* SDNHM 24252 in lateral view. (B) Jugal of *E. paucicarinata* SDNHM 45106 in lateral view. (C) Jugal of *E. nana* SDNHM 52886 in lateral view. (D) Jugal of *G. infernalis* TNHC 18988 in lateral view. (E) Maxilla, lacrimal, and jugal of *E. coerulea* TNHC 58792 in anterolateral view. (F) Maxilla, lacrimal, and jugal of *E. nana* SDNHM 52886 in anterolateral view. All scale bars equal 1 mm. ****f, foramina; J, jugal; J.s, jugal spur; L, lacrimal; Mx.f.pr, facial process of the maxilla; Po.pr, postorbital process.

23. Presence of a jugal spur (quadratojugal process): 0=absent, Fig. 16C; 1=present, Fig. 16A (Gauthier, Estes & de Queiroz, 1988, character 11).

The right jugal of *E. multicarinata* TxVP M- 8993 and left jugals of *E. panamintina* MVZ 191076 and *E. nana* SDNHM 52886 lack an ossified posterior jugal spur. In *E. nana* SDNHM 52886, a jugal spur is absent on the left jugal, but a free-floating ossification resembling the jugal spur is present (Fig. 16C). The jugal spur is longest in some specimens of *Gerrhonotus* (e.g., *G. infernalis* TNHC 18988, Fig. 16D)

24. Number of foramina on the lateral surface of the jugal (Smith, 2009).

A single foramen on the lateral surface of the jugal reportedly occurs in *Elgaria*, while multiple foramina were reported for *Abronia* (Smith, 2009). We found that among specimens of *Elgaria*, the number of foramina on the lateral surface ranges from zero (e.g., right jugal of *E. kingii* SDNHM 24252, Fig. 16A) to four (e.g., *E. paucicarinata* SDNHM 45106, Fig. 16B). Many specimens exhibit bilateral asymmetry in the number of lateral foramina (e.g., *E. kingii* SDNHM 24252). There are four to five foramina on the lateral surface of the jugals in several specimens of *G. infernalis* (TNHC 18988, TNHC 92262) and on the right jugal of *G. parvus* SRSU 5538.

S. Lateral extension of the jugal-lacrimal articulation to overhang the maxilla (Good, 1987, character 66).

Lateral overhang (outward bending of Good (1987)) of the jugal and lacrimal where the two bones articulate dorsal to the maxilla was reported in *Elgaria*, *Gerrhonotus*, and *Barisia* (Good, 1987). In most specimens within our sample, there is some degree of lateral overhang of the lacrimal and the jugal dorsal to the maxilla (Fig. 16F). Specimens are variable on a continuous spectrum, making it difficult to discretize this feature into qualitative states; however, we note that the overhang is subtle or indistinct in many specimens (e.g., *E*. *coerulea* TNHC 58792, Fig. 16E).

### Postfrontal

T. Overlap between the postfrontal and the postorbital (new feature).

**Figure 17:**
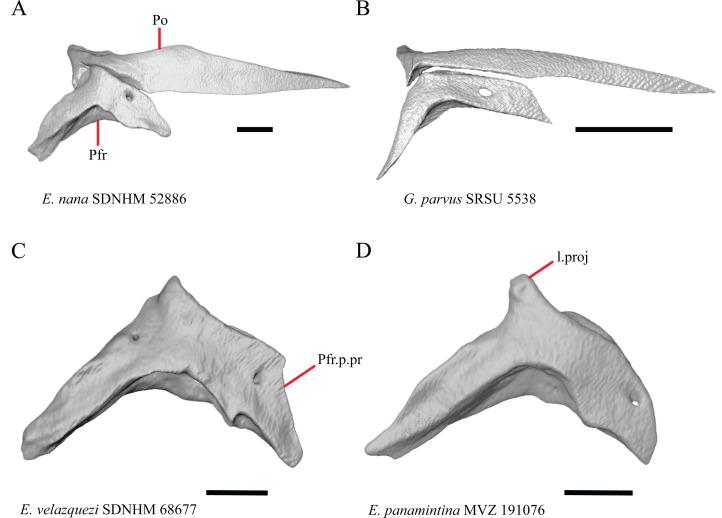
Postfrontals and postorbitals of some species of *Elgaria* and *Gerrhonotus*. (A) Postfrontal and postorbital of *E. nana* SDNHM 52886 in dorsal view. (B) Postfrontal and postorbital of *G. parvus* SRSU 5538 in dorsal view. (C) Postfrontal of *E. velazquezi* SDNHM 68677 in dorsal view. (D) Postfrontal of *E. panamintina* MVZ 191076 in dorsal view. All scale bars equal 1 mm. l.proj, lateral projection; ****Po, postorbital; Pfr, postfrontal; Pfr.p.pr, postfrontal posterior process.

In many specimens, the lateral portion of the postfrontal distinctly overlies the postorbital (Fig. 17A). However, in some specimens including *E*. *coerulea* UF 152969, specimens of *G. parvus*, specimens of *G. lugoi*, and some *G. infernalis* (TxVP M- 7129, TxVP M- 1732, TxVP M- 11412) there is minimal overlap between the postfrontal and the postorbital (Fig. 17B). The amount of overlap varies continuously among specimens, so we did not score this feature in discrete qualitative states.

U. Condition of the lateral edge of the inflection point on the postfrontal (new feature).

The shape of the lateral edge of the inflection point on the postfrontal varies continuously among specimens. The lateral edge ranges from being smooth (e.g., *G. parvus* SRSU 5538, Fig. 17B), to forming a corner (e.g., *E. velazquezi* SDNHM 68677, Fig. 17C), or a distinct, elongated projection (e.g., *E. panamintina* MVZ 191076, Fig. 17D).

### Postorbital

**Figure 18:**
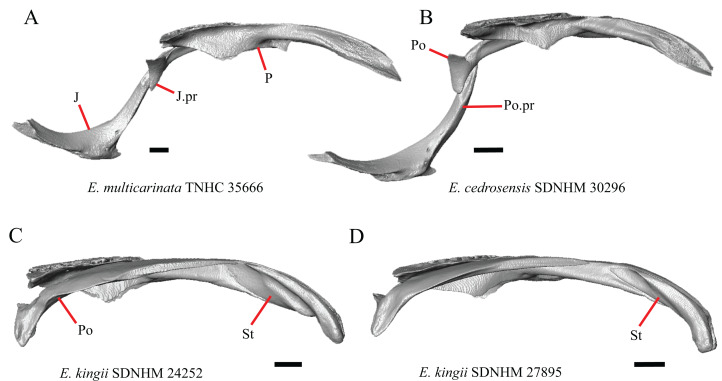
Jugals, parietals, supratemporals, and postorbitals of some species of *Elgaria*. (A) Jugal, postorbital, and parietal of *E. multicarinata* TNHC 35666 in medial view (parietal in lateral view). (B) Jugal, postorbital, and parietal of *E. cedrosensis* SDNHM 30296 in medial view (parietal in lateral view). (C) Postorbital, parietal, and supratemporal of *E. kingii* SDNHM 24252 in lateral view. (D) Postorbital, parietal, and supratemporal of *E. kingii* SDNHM 27895 in lateral view. All scale bars equal 1 mm. ****J, jugal; J. pr, jugal process; Po, postorbital; Po.pr, postorbital process; St, supratemporal.

25. Position of the jugal process of the postorbital relative to the postorbital process of the jugal at the point of articulation: 0=jugal process of the postorbital positioned mostly medial or posterior to the postorbital process of the jugal, Fig. 18A; 1=jugal process of the postorbital lies mostly anterior to the postorbital process of the jugal, Fig. 18B (Good, 1987, character 44).

The jugal process of the postorbital was reported to lie anterior to the jugal only in *Elgaria* (Good, 1987). We found that the jugal process of the postorbital lies anterior to the jugal only in specimens of *E. cedrosensis* (Fig. 18B), *E. nana*, in *E. velazquezi* SDNHM 68678, and in *E. multicarinata* TxVP M- 9004. In *E. multicarinata* TNHC 35666 and *G. infernalis* TxVP M- 1723 the jugal process of the postorbital is positioned posterior to the jugal. The variability in the position of the jugal process of the postorbital likely results from the kinetic nature of the contact between the postorbital and jugal (see character 79 of Gauthier et al. (2012)), leading us to conclude that in its current form this character is not useful for diagnosing *Elgaria*.

26. Position of the posterior tip of postorbital relative to the anterior tip of the supratemporal: 0= posterior tip of postorbital anterior to anterior tip of the supratemporal, Fig. 18D; 1= posterior tip of postorbital and anterior tip of the supratemporal at the same anterior-posterior level or posterior tip of postorbital posterior to anterior tip of the supratemporal, Fig. 18C (similar to Conrad et al. (2011), character 96)

It was reported that having a postorbital extending more than 75% of the length of the supratemporal fenestra (upper temporal fenestra of Evans (2008)) is an unambiguous synapomorphy of *Elgaria* (Conrad et al., 2011). Previous authors noted issues in the construction of this character (Simões et al., 2017). We note that there is also the problem of consistently determining the anterior-posterior length of the supratemporal fenestra because of variation in the length of the fenestra due to variation in the width of postorbital at the anterior end of the supratemporal fenestra, and variation in the length of the fenestra due to the anterior extent and orientation of the supratemporal. We chose to examine the posterior extension of the postorbital relative to the anterior tip of the supratemporal because we were able to score specimens in discrete states. The postorbital extends posterior to the anterior tip of the supratemporal in *E. panamintina* MVZ 75918, *E. kingii* SDNHM 24252 (Fig. 18C), *E. paucicarinata* SDNHM 45106, in several specimens of *E. multicarinata* (CAS 54241, TxVP M- 9005, TxVP M- 9007), and some specimens of *E*. *coerulea* (UF 152969, TxVP M- 8965, TxVP M- 8965) and all specimens we examined of *Gerrhonotus* except *G. lugoi* (LACM 116254, CM 49012) and *G. parvus* (SRSU 5538, SRSU 5537). Both the length of the postorbital and the supratemporal influenced our scoring of the feature and we note that the length of the supratemporal was documented to vary ontogenetically in *E*. *coerulea* (Good, 1995) and generally in lizards (Evans, 2008).

27. Medial expansion of the postorbital at the anterior end of the supratemporal fenestra; 0=relatively unexpanded, Fig. 9A; 1=distinctly expanded medially into the supratemporal fenestra, Fig. 9C (new feature).

The anterior end of the postorbital where it forms a border for the supratemporal fenestra is relatively unexpanded in most specimens (e.g., *E. nana* SDNHM 17102, Fig. 9A). In several specimens of *Elgaria* and *Gerrhonotus* the postorbital is distinctly expanded medially on both postorbitals (e.g., specimens of *E. panamintina*, Fig. 9C), and in others it is expanded only on one side (e.g., *G. infernalis* TNHC 18988, Fig. 9D).

### Quadrate

V. Anteromedial expansion of the pterygoid lamina on the quadrate dorsal to the pterygoid facet (new feature).

**Figure 19:**
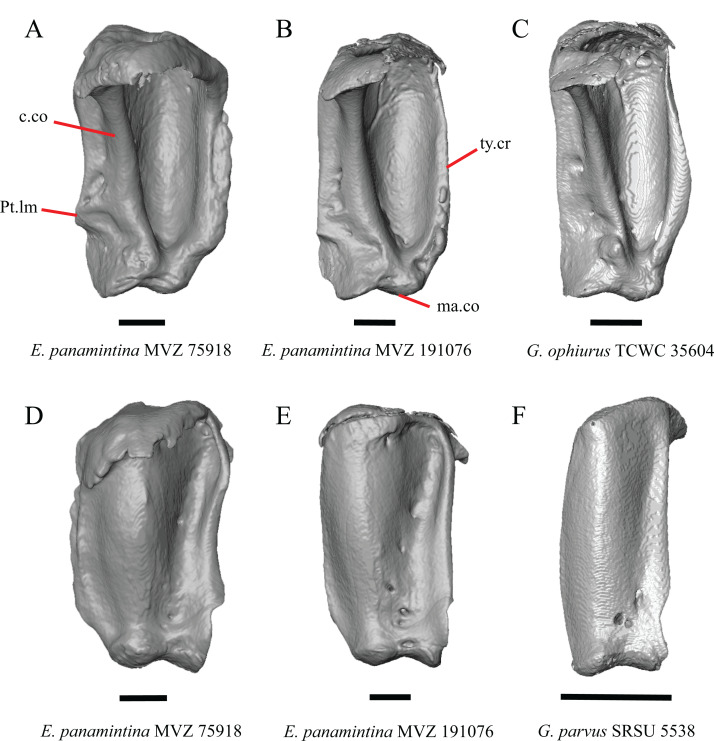
Quadrates of some species of *Elgaria* and *Gerrhonotus*. (A) ****Quadrate of *E. panamintina* MVZ 75918 in posterior view. (B) Quadrate of *E. panamintina* MVZ 191076 in posterior view. (C) Quadrate of *G. ophiurus* TCWC 35604 in posterior view. (D) Quadrate of *E. panamintina* MVZ 75918 in anterior view. (E) Quadrate of *E. panamintina* MVZ 191076 in anterior view. (F) ****Quadrate of *G. parvus* SRSU 5538 in anterior view. All scale bars equal 1 mm. ****c.co, cephalic condyle; ma.co, mandibular condyle; Pt.lm, pterygoid lamina; ty.cr, tympanic crest.

The shape of the medial edge of the quadrate varies intraspecifically in specimens of *Elgaria* and *Gerrhonotus*. The portion of the quadrate pterygoid lamina dorsal to the pterygoid facet ranges from being distinctly medially expanded (e.g., *E. panamintina* MVZ 75918, Fig. 19A), to slightly expanded (e.g., *E. panamintina* MVZ 191076, Fig. 19B), to not expanded medially (e.g., *G. ophiurus* TCWC 35604, Fig. 19C). The continuous range of variation between morphologies precluded us from scoring this feature in discrete qualitative states.

W. Condition of the anterior surface of the quadrate (new feature).

We found a continuous range of variation in the shape of the concave medial portion of the anterior surface of the quadrate (Figs. 19D, 19E, and 19F). The concave medial portion is relatively shallow in specimens of *G. parvus* (Fig. 19F) and *E. multicarinata* TxVP M- 8988. Ontogenetic changes in the shape of the quadrate were reported in lacertid lizards (Barahona & Barbadillo, 1998) and in *Anolis* (Bochaton et al., 2017). Juvenile specimens of *Elgaria* (e.g., *E. multicarinata* TxVP M- 8982) have a shallow concave medial portion of the quadrate.

### Pterygoid

**Figure 20:**
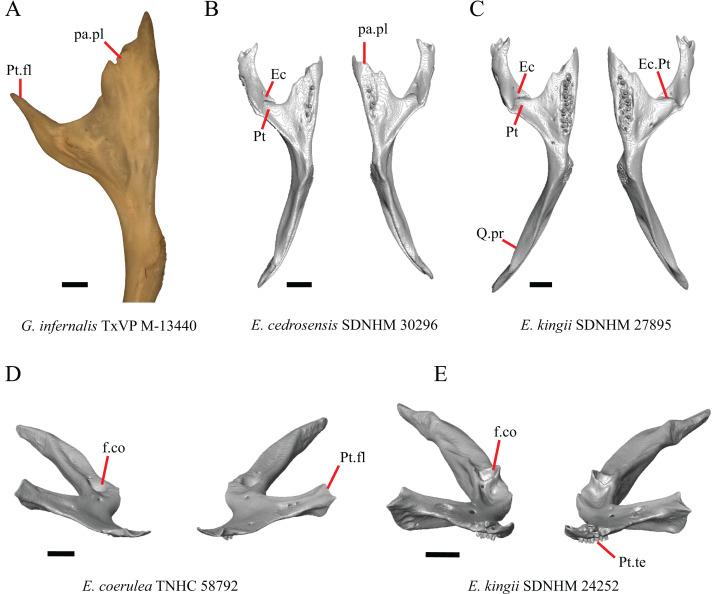
Pterygoids and ectopterygoids of some species of *Elgaria* and *Gerrhonotus*. (A) ****Anterior portion of the pterygoid of *G. infernalis* TxVP M-13440 in ventral view. (B) Ectopterygoids and pterygoids of *E. cedrosensis* SDNHM 30296 in ventral view. (C) Ectopterygoids and pterygoids of *E. kingii *SDNHM 27895 in ventral view. (D) Pterygoids of *E. coerulea* TNHC 58792 in anterior view. (E) Pterygoids of *E. kingii* SDNHM 24252 in anterior view. All scale bars equal 1 mm. ****Ec, ectopterygoid; Ec.Pt, ectopterygoid and pterygoid contact, f.co, fossa columellae; pa.pl, palatal plate; Pt, pterygoid; Pt.fl, pterygoid flange; Pt.te, pterygoid teeth; Q.pr, quadrate process.

28. Pterygoid teeth: 0=no teeth, Fig 20A; 1=small number of tooth positions present in a single row or a small patch, Fig. 20B; 2=many tooth positions present in a large patch, Fig. 20C (Tihen, 1949; modified from Good, 1987, character 91 and 92).

Pterygoid teeth were reported to occur in *Gerrhonotus* and *Elgaria*, albeit reduced in number in *E*. *coerulea* (Good, 1987) and some *Gerrhonotus* (Criley, 1968). Most specimens have a large number of pterygoid teeth arranged in a large patch; however, a reduced number of pterygoid teeth arranged in a single row or small patch is present in some specimens of *E. multicarinata, E. cedrosensis*, *G. parvus*, *G. infernalis*, and *G. ophiurus*. Juvenile specimens of *E. multicarinata* (TxVP M- 8982, TxVP M- 8578) possess a single row of pterygoid teeth, but a juvenile *E. kingii* (TxVP M- 8582) has a large patch of teeth. An unusual condition was observed in *E. panamintina* MVZ 75918, in which pterygoid teeth are absent, but a rugose texture and empty tooth sockets are present on the ventral surface of the palatal plate (see figure 10A of Ledesma & Scarpetta (2018)). *Gerrhonotus infernalis* TxVP M- 13440 is the only specimen in which pterygoid teeth appear to be completely absent (Fig. 20A). Pterygoid teeth were also reported to be absent in one specimen of *E*. *coerulea* (Stebbins, 1958). High variability in the presence and number of pterygoid teeth was previously documented in *Podarcis* (Skawiński, Borczyk & Turniak, 2017), and ontogenetic variation in the number of rows of pterygoid teeth was reported in *Iguana iguana* (Bochaton et al., 2016b). An increased sample size of *Elgaria* and *Gerrhonotus* could capture a greater range of variation in this feature for these genera.

**Figure 21:**
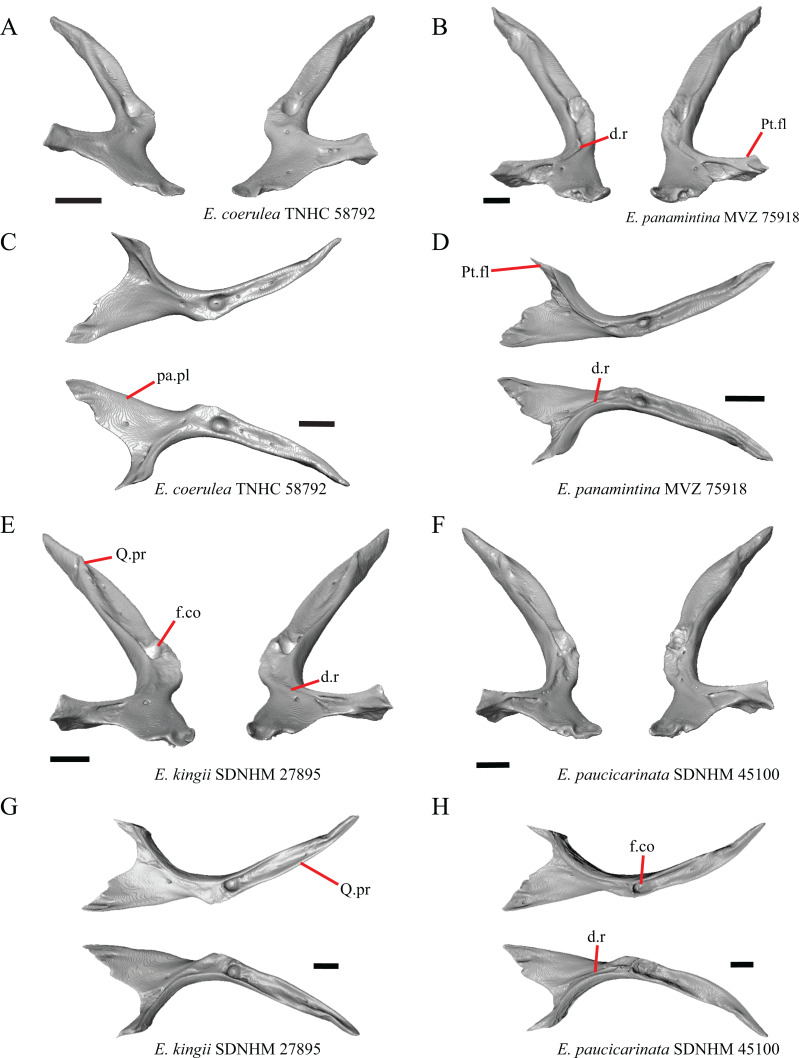
Pterygoids of some species of *Elgaria* and *Gerrhonotus*. (A) Pterygoids of *E. coerulea* TNHC 58792 in anterodorsal view. (B) Pterygoids of *E. panamintina* MVZ 75918 in anterodorsal view. (C) Pterygoids of *E. coerulea* TNHC 58792 in dorsal view. (D) Pterygoids of *E. panamintina* MVZ 75918 in dorsal view. (E) Pterygoids of *E. kingii* SDNHM 27895 in anterodorsal view. (F) Pterygoids of *E. paucicarinata* SDNHM 45100 in anterodorsal view. (G) Pterygoids of *E. kingii* SDNHM 27895 in dorsal view. (H) Pterygoids of *E. paucicarinata* SDNHM 45100 in dorsal view. All scale bars equal 1 mm. ****d.r, dorsal ridge; f.co, fossa columellae, pa.pl, palatal plate; Pt.fl, pterygoid flange; Q.pr, quadrate process.

29. Condition of a dorsal ridge on the pterygoid, distinct from the lateral-most edge of the pterygoid and the flattened palatal plate, beginning anterior to the fossa columellae and running along the lateral edge of the palatal plate to the ectopterygoid facet on the pterygoid flange: 0=absent, Figs. 20D, 21A, and 21C; 1=present, Figs. 21B and 21D (new feature).

Some specimens of *Elgaria* possess a dorsal ridge that is distinct from the lateral edge of the pterygoid, which serves as the insertion point for the superficial pseudotemporal muscle (Villa & Delfino, 2019), and the palatal plate. This ridge is most distinct in specimens of *E. panamintina*, *E. paucicarinata* (but only the left pterygoid of *E. paucicarinata* SDNHM 45100, Figs. 21F and 21H), *E. velazquezi* SDNHM 68678, the left pterygoid of *E. kingii* SDNHM 27895, and in *G. liocephalus* TCWC 8585. A raised surface on the posterior surface of the palatal plate is present on some specimens (e.g., the right pterygoid of *E. kingii* SDNHM 27895, Figs. 21E and 21G) but it is short and does not extend anteriorly to the ectopterygoid facet on the pterygoid flange.

X. Condition of the border of the fossa columella on the pterygoid (new feature).

The border of the fossa columella varies continuously among specimens. The border ranges from being developed into tall prominent ridges surrounding the fossa (e.g., *E. kingii* SDNHM 24252, Fig. 20E) to being close to level with the surrounding area on the pterygoid (e.g., *E. coerulea* TNHC 58792, Fig. 20D).

### Epipterygoid

**Figure 22:**
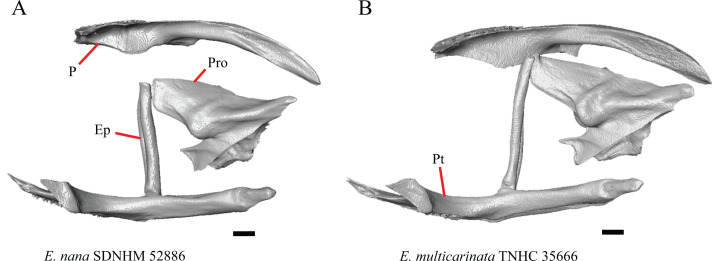
Pterygoids, epipterygoids, parietals, and prootics of some species of *Elgaria*. (A) Parietal, epipterygoids, pterygoids, and prootic of *E. nana* SDNHM 52886 in lateral view. (B) Parietal, epipterygoids, pterygoids, and prootic of *E. multicarinata* TNHC 35666 in lateral view. All scale bars equal 1 mm. ****Ep, epipterygoid; P, parietal; Pro, prootic; Pt, pterygoid.

30. Epipterygoid contact with parietal: 0=Epipterygoid does not contact the parietal, Fig. 22A; 1= Epipterygoid contacts the parietal, Fig. 22B (adapted from McDowell & Bogert (1954)).

Contact between the epipterygoid and the parietal is only seen in the three CT scanned specimens *E. multicarinata* TNHC 35666 (Fig. 22B), *E. kingii* UF 74645, and *G. parvus* SRSU 5537. However, eight dry skeletons of *Elgaria* have contact between the bones. This discrepancy may be a result of shrinkage during the skeletonization process in which bones are pulled together by the drying of tissue. It may also be a result of ontogenetic variation, with older and younger specimens having an epipterygoid that is closer or farther away from the parietal, respectively (Evans, 2008).

### Ectopterygoid

31. Shape of the contact between the ectopterygoid and the pterygoid from a ventral view: 0=straight, fig. 2B of Good (1987); 1=curved (u-shaped), Fig. 20B (Good, 1987, character 37).

A curved, “u-shaped” (Good, 1987: 289), contact between the ectopterygoid and the pterygoid was reported in *Elgaria* and *Gerrhonotus* (Good, 1987). We found that the shape of the contact between the ectopterygoid and pterygoid was curved in all specimens; however, we note that the condition of *G. infernalis* TxVP M- 1723 and the left side of *E. kingii* SDNHM 27895 (Fig. 20C) closely resembles the straight condition illustrated by Good (1987) for species belonging to other gerrhonotine genera. Furthermore, exemplars of the straight condition illustrated by Good (1987) were not all depicted as having strictly straight contact, and some were depicted as being bilaterally asymmetrical (e.g., figs. 2A and 3B of Good, 1987).

Y. Length of a lateral ‘spur’ on the ectopterygoid where the bone meets the maxilla. (Good, 1987, character 38).

**Figure 23:**
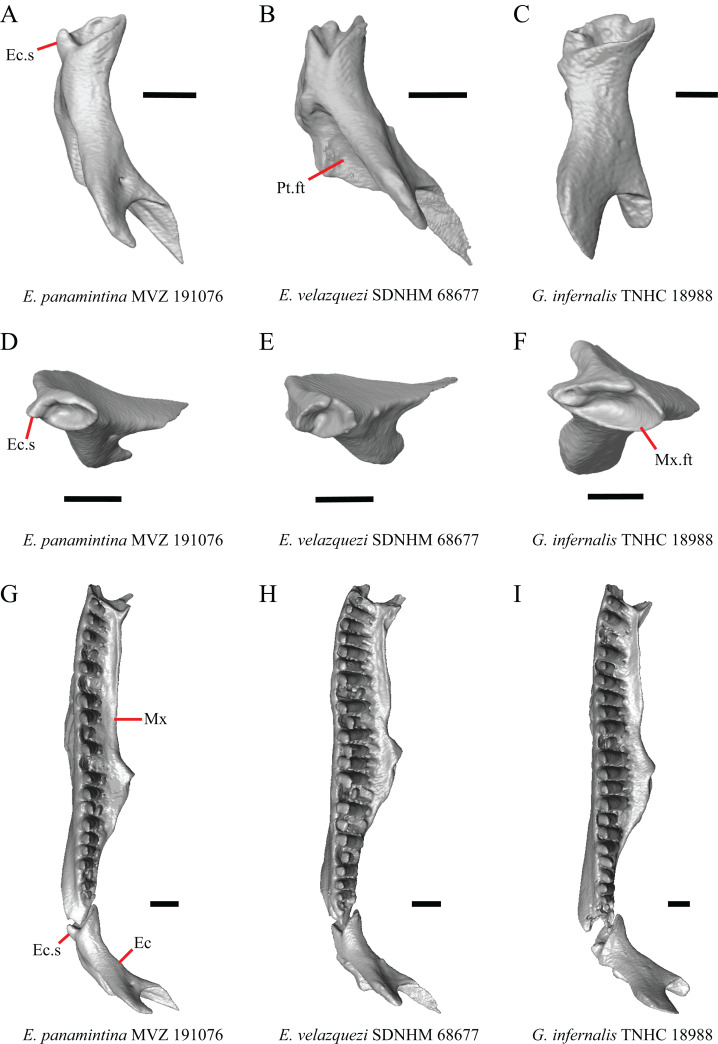
Maxillae and ectopterygoids of some species of *Elgaria* and *Gerrhonotus*. (A) Ectopterygoid of *E. panamintina* MVZ 191076 in ventral view. (B) Ectopterygoid of *E. velazquezi* SDNHM 68677 in ventral view. (C) Ectopterygoid of *G. infernalis* TNHC 18988 in ventral view. (D) Ectopterygoid of *E. panamintina* MVZ 191076 in anterior view. (E) Ectopterygoid of *E. velazquezi* SDNHM 68677 in anterior view. (F) Ectopterygoid of *G. infernalis* TNHC 18988 in anterior view. (G) Ectopterygoid and maxilla of *E. panamintina* MVZ 191076 in ventral view. (H) Ectopterygoid and maxilla of *E. velazquezi* SDNHM 68677 in ventral view. (I) Ectopterygoid and maxilla of *G. infernalis* TNHC 18988 in ventral view (The maxilla in this specimen may be slightly broken or have bone degradation resulting in a notched posterior end). All scale bars equal 1 mm. ****Ec, ectopterygoid; Ec.s, Mx, maxilla; Mx.ft, maxillary facet; Pt.ft, pterygoid facet.

An elongate lateral ‘spur’ on the ectopterygoid was reported to differentiate *Elgaria* from other gerrhonotines (Good, 1987). We found that the presence of a spur is related to whether the bone is viewed in articulation or in isolation. A ‘spur’ may be a distinct projection in the isolated ectopterygoid, as in *E. panamintina* MVZ 191076 (Figs. 23A, 23D, and 23G). In many specimens, there is no distinct projection or ‘spur’ on the isolated ectopterygoid; however, when observed in ventral view on an articulated skull, the posterior orbital process of the maxilla fits into a notch on the anterior end of the ectopterygoid making the portion of the ectopterygoid just lateral to this notch resembles a distinct projection or ‘spur’ (e.g., *E. velazquezi* SDNHM 68677, Figs. 23B, 23E, and 23H). The lateral portion of this notch is most indistinct in some specimens of *G. infernalis* (TxVP M- 11411, THNC 18988) (Figs. 23C, 23F, and 23I) and *E. velazquezi* SDNHM 68678. Juvenile specimens of *E. multicarinata* also do not have a distinct lateral portion of the ventral notch (e.g., *E. multicarinata* TxVP M- 8982). We chose not to score this feature in discrete states because we could not make a clear qualitative distinction among specimens in the shape and distinctiveness of the notch or spur. However, we note that the notch on the ectopterygoid in many specimens of *Gerrhonotus* is not unlike that present in most specimens of *Elgaria*.

### Septomaxilla

**Figure 24:**
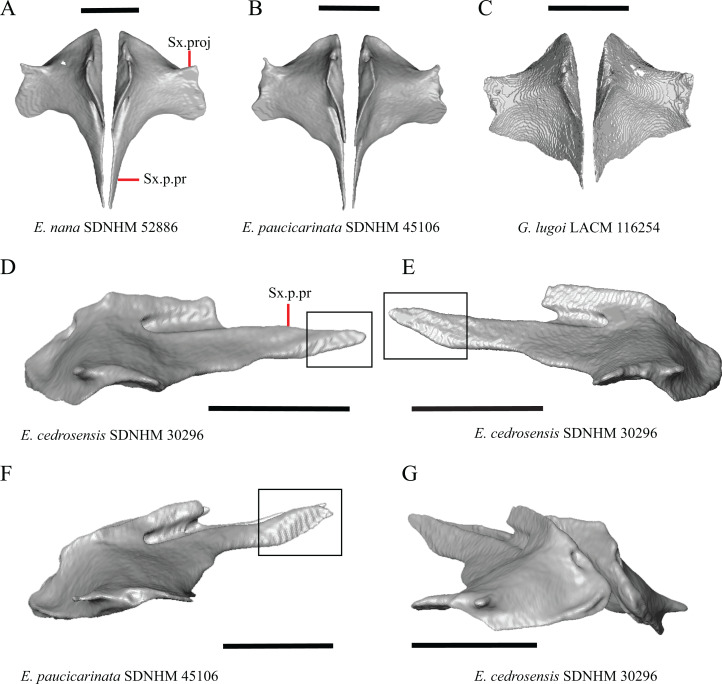
Septomaxillae of some species of *Elgaria* and *Gerrhonotus*. (A) Septomaxillae of *E. nana* SDNHM 52886 in dorsal view. (B) Septomaxillae of *E. paucicarinata* SDNHM 45106 in dorsal view. (C) Septomaxillae of *G. lugoi* LACM 116254 in dorsal view. (D) Septomaxilla of *E. cedrosensis* SDNHM 30296 in lateral view. (E) Septomaxilla of *E. cedrosensis* SDNHM 30296 in lateral view. (F) Septomaxillae of *E. paucicarinata* SDNHM 45106 in lateral view. (G) Septomaxillae of *E. cedrosensis* SDNHM 30296 in anterolateral view. All scale bars equal 1 mm. ****Sx. proj, anterolateral projection of septomaxilla; Sx.p.pr, posterior process of the septomaxilla. Black boxes demarcate the posterior end of the posterior process septomaxilla.

32. Length of the posterior process (septal process of Criley, 1968; medial process of Evans (2008)) of the septomaxilla: 0=relatively elongate, Fig. 24A; 1=relatively short, Fig. 24C (Good, 1987, character 62).

The posterior process of the septomaxilla is shortened in *E. coerulea* TNHC 58792 and in specimens of *G. lugoi* relative to other specimens of *Elgaria* and *Gerrhonotus*.

33. Shape of the posterior process (septal process of Criley, 1968) of the septomaxilla: 0=straight, Fig. 24D; 1=curved dorsally at the posterior end, Figs. 24E and 24F (Good, 1987, character 64).

A dorsally curved posterior process of the septomaxilla was reported previously to diagnose *Elgaria* (Good, 1987). We found that the posterior process is straight in many specimens of *Elgaria*, including specimens of *E. cedrosensis* (only the left septomaxilla of SDNHM 30296, Fig. 24D), *E. kingii* (SDNHM 24252, the left septomaxilla of SDNHM 27895), *E. paucicarinata* SDNHM 45100, *E*. *coerulea* (CAS 14509, UF 152969), and some specimens of *E. multicarinata* (TNHC 35666, TNHC 4478, TxVP M- 8975). Furthermore, a dorsally curved posterior process of the septomaxilla is present on the left septomaxilla of *G. lugoi* LACM 116254 and the right septomaxilla of *G. infernalis* TxVP M- 11414. Care should be employed when scoring this feature in anterolateral view on an articulated skull (i.e., viewed through the naris) because a non-linear ventral border of the posterior process of the septomaxilla may give the impression that the process curves farther dorsally than it appears in lateral view (e.g., *E. cedrosensis* SDNHM 30296, Fig 24G).

34. Condition of a distinct anterolateral projection (spur) on the septomaxilla: 0=absent; 1=present, Fig. 24A (Good, 1987, character 60).

An anterolateral ‘spur’ on the septomaxilla was reported in *Elgaria* and *Gerrhonotus* and was hypothesized to be homologous to a flange on the septomaxilla present in other anguids (Good, 1987). All specimens have some type of anterolateral projection; however, the morphology of such a projection varies considerably among specimens. The projection may be thin and elongated (e.g., *E. paucicarinata* SDNHM 45106, Fig. 24B) or it may be broad (e.g., *G. lugoi* LACM 116254, Fig. 24C). The morphology of the projection may also be bilaterally asymmetrical (e.g., *E. velazquezi* SDNHM 68677). It is possible that the shape of the spur varies ontogenetically, and Evans (2008) noted that in lizards generally flanges and processes on the septomaxilla result from an increased ossification through ontogeny.

### Vomer

**Figure 25:**
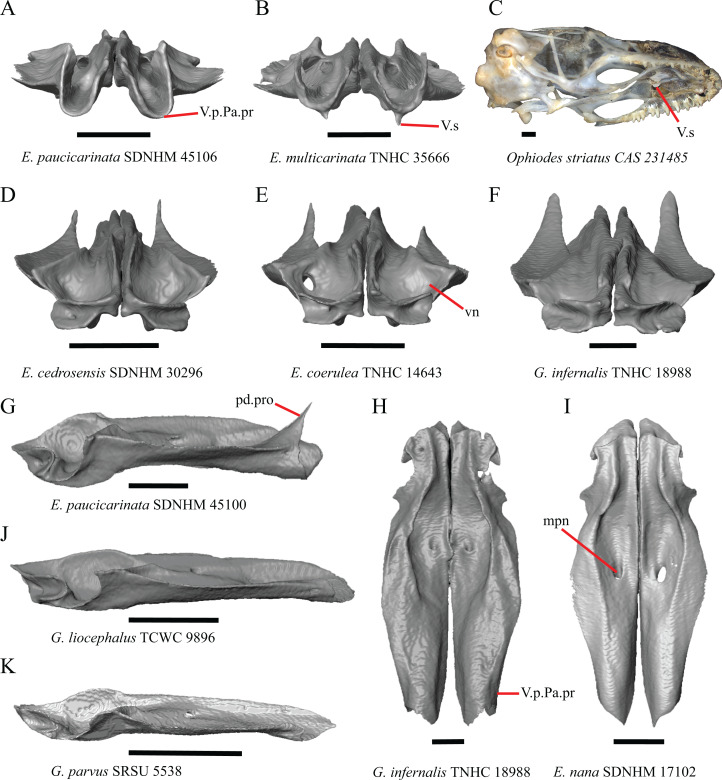
Vomers of some species of *Elgaria* and *Gerrhonotus* and the skull of *Ophiodes striatus*. (A) ****Vomers of *E. paucicarinata* SDNHM 45106 in posterior view. (B) Vomers of *E. multicarinata* TNHC 35666 in posterior view. (C) Skull of *Ophiodes striatus* CAS 231485 in ventrolateral view. (D) Vomers of *E. cedrosensis* SDNHM 30296 in anterior view. (E) Vomers of *E. coerulea* TNHC 14643 in anterior view. (F) Vomers of *G. infernalis* TNHC 18988 in anterior view. (G) Vomers of *E. paucicarinata* SDNHM 45100 in lateral view. (H) Vomers of *G. infernalis* TNHC 18988 in ventral view. (I) Vomers of *E. nana* SDNHM 17102 in ventral view. (J) Vomers of *G. liocephalus* TCWC 9896 in lateral view. (K) Vomers of *G. parvus* SRSU 5538 in lateral view. All scale bars equal 1 mm. mpn, foramen for the medial palatine nerve; pd.pro, posterodorsal lamina; vn, vomeronasal region; V.p.Pa.pr, posterior palatine process of vomer; V.s, vomer spur.

35. Angle that the foramen for the medial palatine nerve penetrates the vomer: 0=penetrates at an anteriorly inclined angle, Fig. 25H; 1=penetrates vertically through the bone, Fig. 25I (Good, 1987, character 28).

All gerrhonotines besides *Mesaspis* (now synonymized with *Abronia*) reportedly possess a medial palatine nerve that pierces the vomer via an anteriorly angled foramen (Good, 1987). We found that in most examined specimens of *Elgaria* and *Gerrhonotus*, the foramen pierces the bone at an inclined angle. The foramen on the left vomer of *G. ophiurus* TCWC 35604 and the left vomer of *E. nana* SDNHM 17102 (Fig. 25I) pierces the bone vertically. In *E. multicarinata* TNHC 35666, the left foramen is not inclined and instead pierces the bone horizontally and empties anteriorly into a large hole in the vomer that we did not observe in any other specimen (Fig. 25B). The foramen on the right vomer of *E. kingii* SDNHM 27895 penetrates closer to vertical relative to its contralateral element.

36. Presence of spur on the posteroventral surface of the vomer: 0=absent, Fig. 25A; 1=present, Fig. 25B (new feature).

The vomers of *E. multicarinata* TNHC 35666 are unusual with respect to other gerrhonotines in that they have a small spur on the ventral surface of the palatine processes. This morphology is similar to bony spurs reported in *Diploglossus* and *Ophiodes* (Evans, 2008; e.g., *Ophiodes striatus* CAS 231485, Fig. 25C).

37. Condition of a lamina or projection on the lateral edge of the posterior palatine process of the vomer: 0=short or absent, Fig. 25K; 1=tall or long, Fig. 25G (new feature).

The morphology of a lamina on the dorsolateral surface of the posterior palatine process of the vomer is variable among specimens. Most specimens of *Elgaria* possess a tall lamina that may extend into a posterodorally pointed projection (e.g., *E. paucicarinata* SDNHM 45100, Fig. 25G) or may not have a pointed projection (e.g., *E*. *coerulea* and *E. nana*). A tall lamina without a pointed projection is also present in *G. liocephalus* TCWC 9896 (Fig. 25J) and *G. lugoi* CM 49012. Specimens of *G. parvus* are unique in having a short lamina (Fig. 25K).

38. Presence of a large foramen near the posterolateral margin of the vomeronasal concavity: 0=absent, Fig. 25D; 1=present, Fig. 25E (new feature).

We found that there is a large foramen near the posterolateral margin of the vomeronasal concavity in *E. kingii* SDNHM 27895 and on the right vomer of *E*. *coerulea* TNHC 14643 (Fig. 25E).

Z. Length of the palatine process of the vomer (Good, 1987, character 25).

The palatine process of the vomer of *Gerrhonotus* was described as being elongated relative to other gerrhonotines (Good, 1987). While the lengths of the vomers of specimens of *G. infernalis* and possibly *G. lugoi* appear somewhat different than those of specimens of *Elgaria* and *G. parvus*, when we isolated the vomers, we did not observe an unambiguous qualitative distinction between a short and a long palatine process (Figs. 25H and 25I). Future studies using quantitative methods like geometric morphometrics may discover distinct differences related to this morphology.

AA. Condition of the posterolateral border of the vomeronasal concavity on the vomer (new feature).

The posterolateral border of the vomeronasal concavity is characterized by a steep and distinct ridge that separates the nasal and vomeronasal regions of the vomer in most specimens of *Elgaria* (e.g., *E. cedrosensis* SDNHM 30296, Fig. 25D). In some specimens of *G. infernalis* (TNHC 18988, TNHC 92262) and *G. lugoi* LACM 116254 this ridge is somewhat shorter resulting in a shallowly inclined posterolateral border of the vomeronasal concavity and a less distinct separation from the nasal region (Fig. 25F). We did not score this character because all specimens have a ridge and variation is continuous which results in specimens not easily being separated into distinct qualitative states.

### Palatine

**Figure 26:**
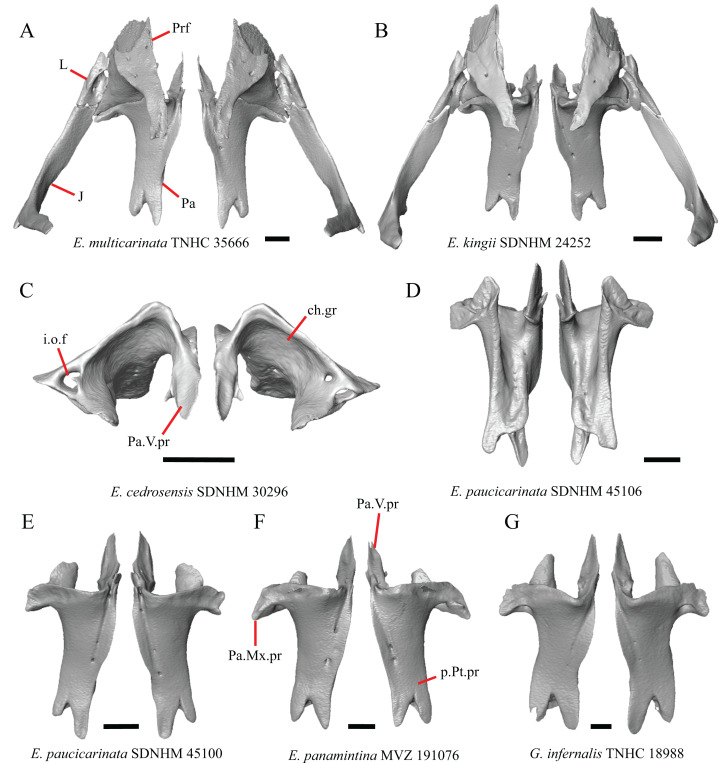
Prefrontals, lacrimals, palatines, and jugals of some species of *Elgaria* and *Gerrhonotus*. (A) ****Prefrontals, lacrimals, palatines, and jugals of *E. multicarinata* TNHC 35666 in dorsal view. (B) Prefrontals, lacrimals, palatines, and jugals of *E. kingii* SDNHM 24252 in dorsal view. (C) Palatines of *E. cedrosensis* SDNHM 30296 in anterior view. (D) Palatines of *E. paucicarinata* SDNHM 45106 in ventral view. (E) Palatines of *E. paucicarinata* SDNHM 45100 in dorsal view. (F) Palatines of *E. panamintina* MVZ 191076 in dorsal view. (G) Palatines of *G. infernalis* TNHC 18988 in dorsal view. All scale bars equal 1 mm. ch.gr, choanal groove; ****i.o.f, infraorbital foramen; J, jugal; L, lacrimal; Pa, palatine; Pa.Mx.pr, maxillary process of the palatine; p.Pt.pr, posterior pterygoid process; Prf, prefrontal; Pa.V.pr, vomerine process of the palatine.

39. Presence of palatine teeth: 0=absent, Fig. 26D; 1=present (Fitch, 1938).

Species of *Elgaria* (then classified in the genus *Gerrhonotus*) were reported by Fitch (1938) to possess palatine teeth. No specimen in our sample has palatine teeth and it is likely that the author intended to reference teeth on the pterygoid instead.

40. Contact between the palatine and the jugal: 0=present, Fig. 26A; 1=absent, Fig. 26B (Good, 1987, character 35).

The prefrontal was reported previously to exclude contact between the palatine and jugal in *Gerrhonotus* and *Mesaspis* (now synonymized with *Abronia*) (Good, 1987). The lacrimal excludes contact between the palatine and the jugal in *E*. *kingii* SDNHM 24252 (Fig. 26B) and the left side of *E*. *coerulea* TNHC 58792, a condition unique among our sample of gerrhonotines. Among *Elgaria*, the palatine and jugal contact each other only in *E. panamintina* and in most specimens of *E. multicarinata* and *E*. *coerulea*. Among specimens of *Gerrhonotus*, the palatine and jugal are in contact in *G. infernalis* TNHC 92262, *G. parvus* SRSU 5538, and on the right side of *G. parvus* SRSU 5537 (the condition on the left side could not be determined due to bone deterioration). In other specimens of *G. infernalis* (TxVP M- 7129, TxVP M- 11411) the palatine contacts the jugal on one side but not on the other.

41. Presence of a separate foramen on the lateral surface of the choanal groove proximal to the infraorbital foramen: 0=absent (left palatine of *E. cedrosensis* SDNHM 30296), Fig. 26C; 1=present (right palatine of *E. cedrosensis* SDNHM 30296), Fig. 26C (Gauthier et al., 2012, character 244).

There is a separate foramen on the lateral surface of the choanal groove that opens posteriorly into the infraorbital foramen on the left palatine of *E. cedrosensis* SDNHM 30296 (Fig. 26C), the left palatine of *E. paucicarinata* SDNHM 45100, and the right palatine of *E. multicarinata* TxVP M- 8987. The presence of two anterior openings for the infraorbital canal was previously reported in other lizards (e.g., *Cordylus mossambicus*) (Gauthier et al., 2012); however, the presence of two anterior openings has not been previously reported in gerrhonotines.

42. Presence of a flange of bone proximal to the anterior opening of the infraorbital foramen: 0=absent (right palatine of *E. cedrosensis* SDNHM 30296), Fig. 26C; 1=present (left palatine of *E. cedrosensis* SDNHM 30296), Fig. 26C (new feature).

In *E*. *coerulea* TNHC 58792, the right palatine of *E. cedrosensis* SDNHM 30296 (Fig. 26C), and *G. infernalis* TxVP M- 13441, there is a small flange of bone near the anterior opening of the infraorbital foramen. It is possible that the presence of a flange may be related to the presence of two anterior openings for the infraorbital canal because *E. cedrosensis* SDNHM 30296 has a flange on the right palatine and a foramen on the left palatine; however, on all other specimens either a flange or two openings for the infraorbital canal are present.

AB. Shape of the maxillary process of the palatine (modified from Good, 1987, character 32).

The maxillary process of the palatine was described as robust in *Gerrhonotus*, *Elgaria*, and *Barisia* relative to *Mesaspis* (now synonymized with *Abronia*) and *Abronia* (Good, 1987). We found considerable intra- and interspecific variation in the shape of the maxillary process among *Elgaria* and *Gerrhonotus*. The maxillary process extends farthest posteriorly and is posteriorly pointed in specimens of *E. panamintina* (Fig. 26F), *G. liocephalus*, most specimens of *E. multicarinata* except for *E. multicarinata* (TxVP M- 8993, TxVP M- 8987), and most specimens of *G. infernalis* except for *G. infernalis* (TxVP M- 7129, TNHC 18988, Fig. 26G). In other species, the shape of the process varies intraspecifically and is somewhat shorter and blunter (Fig. 26E). Variation in whether the maxillary process possesses a more laterally or posteriorly oriented tip may influence whether the process is interpreted as short or long in articulated specimens. The morphology of the maxillary process of the palatine may also influence whether the palatine and jugal contact one another (our feature 40). Most specimens with an elongate maxillary process also possess contact between the palatine and the jugal. This is exemplified by *G. infernalis* TxVP M- 7129, which has contact on the left side where the maxillary process is somewhat longer but not on the right side where the process is shorter. An elongated maxillary process may, therefore, result in the contact with the jugal; however, this is contradicted by *E. multicarinata* (TxVP M- 9005, TxVP M- 9007) and *G. infernalis* TxVP M- 12353, in which an elongated maxillary process of the palatine does not contact the jugal. We did not score this character because a continuous spectrum of variation in the shape and length of the maxillary process of the palatine among specimens did not allow for consistent assignment to distinct states.

AC. Condition of a dorsomedial flange on the vomerine process of the palatine (Good, 1987, character 33).

A “pronounced dorsomedial flange, present on the dorsal surface of the vomerine process…” was reported in *Gerrhonotus*, *Elgaria*, and *Barisia* (Good, 1987:289). We were unable to determine which flange Good (1987) referred to because there were multiple features on the dorsomedial surface of the vomerine process of the palatine that could have been referenced. When the palatine is viewed anteriorly, the anterior end of an upturned medial edge of the palatine has the appearance of a dorsomedial flange (see figure 17 of Ledesma & Scarpetta (2018)). This is similar to examining an articulated skull in anterior view through the naris. When we viewed the palatine in isolation, we found that there are also small projections on the medial surface of the vomerine process in some specimens that are variable in size and position (e.g., *E. kingii* SDNHM 24252, Fig. 26B). We chose not to score this feature because of our uncertainty as to which structure was intended by Good (1987).

AD. Posterior extension of the lateral edge of the posterior palatine process (Good, 1987, character 34).

It was reported that in ventral view, “…the lateral edge of the pterygoid process [=posterior palatine process] projects much farther posteriorly in all other genera than in *Gerrhonotus*…” (Good, 1987:289). This morphology was likely scored by Good (1987) with the pterygoid and palatine in articulation. We did not observe a difference in the posterior extent of the lateral edge of the posterior palatine process between specimens of *Elgaria* relative to specimens of *Gerrhonotus*. We instead found that the nature of articulation between the palatine and pterygoid is variable, likely because of the kinetic nature of the articulation between the two bones (Frazzetta, 1983). When we examined the palatine in isolation, we found a large amount of intraspecific variation and bilateral asymmetry in the lengths of the two projections on the posterior palatine process. The medial projection ranges from projecting far posteriorly relative to the lateral projection (e.g., specimens of *E. paucicarinata*, Fig. 26E) to being relatively equal in posterior extent to the lateral projection (e.g., specimens of *E. panamintina*, Fig. 26F). Some specimens exhibit bilateral asymmetry in this feature (e.g., *G. infernalis* TNHC 18988, Fig. 26G). There was a continuous spectrum of variation in the lengths of the projections on the posterior palatine process which prevented us from scoring this feature in discrete qualitative states.

### Orbitosphenoid

**Figure 27:**
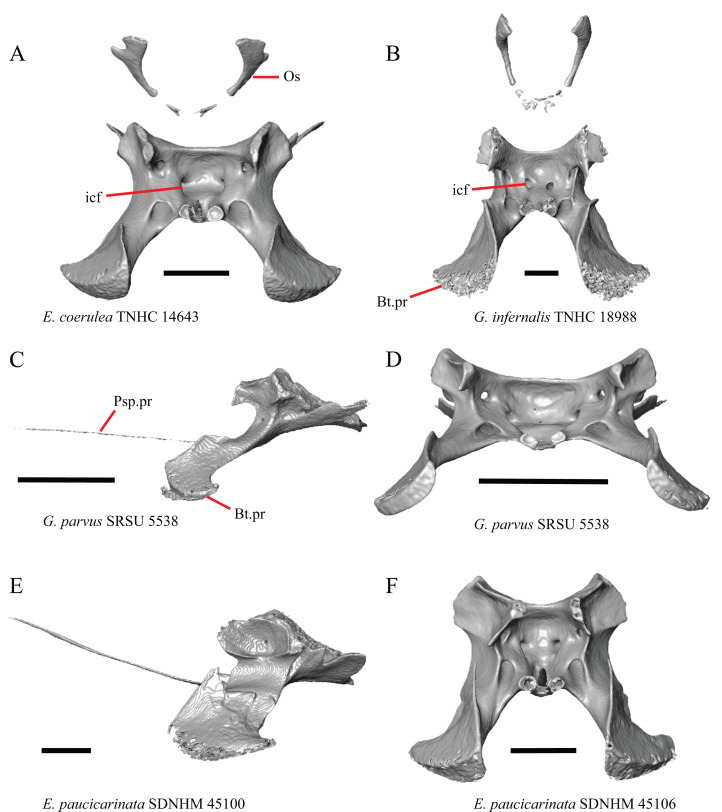
Sphenoids and orbitosphenoids of some species of *Elgaria* and *Gerrhonotus*. (A) Sphenoid and orbitosphenoids of *E. coerulea* TNHC 14643 in anterior view. (B) Sphenoid and orbitosphenoids of *G. infernalis* TNHC 18988 in anterior view. (C) Sphenoid of *G. parvus* SRSU 5538 in lateral view. (D) Sphenoid of *G. parvus* SRSU 5538 in anterior view. (E) Sphenoid of *E. paucicarinata* SDNHM 45100 in lateral view. (F) Sphenoid of *E. paucicarinata* SDNHM 45106 in anterior view. All scale bars equal 1 mm. Bt.pr, basipterygoid process; icf, internal carotid foramen; Os, orbitosphenoid; Psp.pr, parasphenoid process.

43. Morphology of the head of the orbitosphenoid: 0=not bifurcated, Fig. 27B; 1=bifurcated, Fig. 27A (new feature).

The head of the orbitosphenoid is bifurcated in specimens of *E. panamintina*, *E. kingii* SDNHM 24252, *E. paucicarinata* SDNHM 45106, and *E. coerulea* TNHC 14643 (Fig. 27A). Although the morphology of the orbitosphenoid changes with ontogeny in iguanines (de Queiroz, 1987), the morphology of the orbitosphenoid was reported to be independent of ontogeny in polychrotids (Torres-Carvajal, 2003). Further investigation is needed to evaluate patterns of ontogenetic variation in the shape of the orbitosphenoid in gerrhonotines.

### Supraoccipital

**Figure 28:**
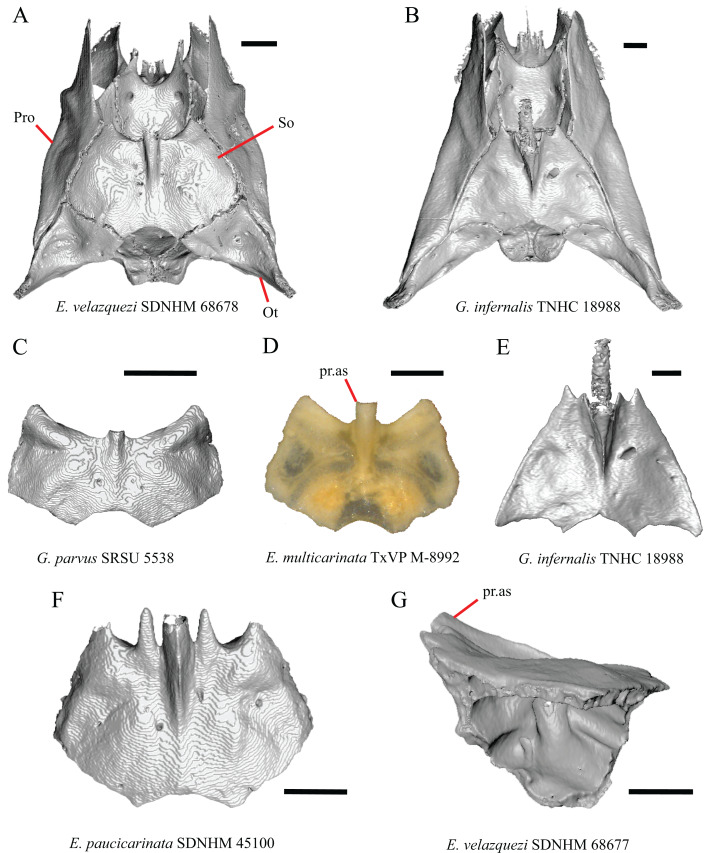
Braincases and supraoccipitals of some species of *Elgaria* and *Gerrhonotus*. (A) Braincase of *E. velazquezi* SDNHM 68678 in dorsal view. (B) ****Braincase of *G. infernalis* TNHC 18988 in dorsal view. (C) Supraoccipital of *G. parvus* SRSU 5538 in dorsal view. (D) Supraoccipital of *E. multicarinata* TxVP M-8992 in dorsal view. (E) Supraoccipital of *G. infernalis* TNHC 18988 in dorsal view. (F) Supraoccipital of *E. paucicarinata* SDNHM 45100 in dorsal view. (G) Supraoccipital of *E. velazquezi* SDNHM 68677 in lateral view. ****All scale bars equal 1 mm. Ot, otooccipital; pr.as, ascending process; Pro, prootic. **** ****

44. Position of the posterolateral tip of the dorsal surface of the supraoccipital where it articulates with the prootic and otooccipital: 0=posterolateral tip is positioned anterior relative to the posterior-most extent of the supraoccipital, Figs. 28A, 28C, and 28D; 1=the posterolateral tip is positioned level or nearly level to the posterior-most extent of the supraoccipital where the bone forms a portion of the bordering of the foramen magnum, Figs. 28B and 28E (new feature).

The lateral corner of the supraoccipital where it contacts the otooccipital and the prootic is positioned level or nearly level to the posterior-most extent of the supraoccipital in examined specimens of *G. infernalis* (except for TxVP M- 13442). In all other species the posterolateral tip is located more anteriorly, although in *E. multicarinata* TxVP M- 8993 the posterolateral tip is located slightly closer to the posterior-most extent of the supraoccipital compared to other *Elgaria*.

AE. Width of supraoccipital and shape of anterior margin in dorsal view (Good, 1987, character 71).

The supraoccipital in *Abronia* (=*Mesaspis*) *moreletii* was reported to be wider and shorter than the supraoccipital of other gerrhonotines (Good, 1987). We found that many specimens of *Elgaria* and *Gerrhonotus* possess a morphology similar to that description, in which the supraoccipital is much wider than it is long (e.g., *G. parvus* SRSU 5538, Fig. 28C). The shape of the supraoccipital was reported to vary ontogenetically in other anguimorphs (Bever, Bell & Maisano, 2005) and we found that the shape of the supraoccipital is wide relative to its anteroposterior length in juvenile specimens of *Elgaria* and small specimens of *Gerrhonotus*. This provides evidence that the relative width-to-length of the supraoccipital varies ontogenetically and may help explain the continuous spectrum of variation observed in our sample. Additionally, we noticed that the anterolateral end of the supraoccipital projects far anteriorly relative to the base of the ascending process in several specimens with a wide and short supraoccipital (e.g., *G. parvus* and *E. multicarinata* TxVP M- 12129). This morphology may be correlated to how wide the bone is perceived to be relative to its length; however, in *E. multicarinata* TxVP M- 8992, which has a supraoccipital that is not especially wide relative to its length, the anterolateral part of the supraoccipital projects far anteriorly relative to the base of the ascending process (Fig. 28D). *Elgaria paucicarinata* SDNHM 45100 is unusual in that long projections are present on either side of the ascending process (Fig. 28F).

AF. Angle between the ascending process (processus ascendens of Evans, 2008) and the main body of the supraoccipital (Good, 1987, character 72).

*Gerrhonotus* was reported to possess an ascending process (medial ascendant process of Good, 1987) that “…at its anterior end makes a sharper angle with the main body of the element…” (Good, 1987:291). We had difficulty consistently examining the angle between the process and the main body of the bone for several reasons. First, because the dorsal surface of the supraoccipital is not flat, it is difficult to determine a horizontal plane by which to measure the angle consistently. Second, the inclination of the dorsal surface of the supraoccipital varies among specimens. Some specimens (e.g., *E. velazquezi* SDNHM 68677 and *G. infernalis* TNHC 18988) have an inclined anterior end of the supraoccipital (Fig. 28G), which confounds a comparison between the angle between the ascending process and the main body of the supraoccipital between all specimens. Nonetheless, we did not observe a distinct qualitative difference in the angle of the ascending process between specimens of *Gerrhonotus* and *Elgaria*.

### Sphenoid

45. In anterior view, direction of the anterior opening for internal carotid foramen: 0=opening faces anteromedially, Figs. 27A and 27F; 1=opening faces anteriorly, Fig. 27B (new feature).

In all examined specimens of *Elgaria*, the anterior openings for the internal carotid foramina face anteromedially. In most specimens of *G. infernalis*, the anterior openings for the internal carotid foramina face anteriorly. However, the opening for the right internal carotid foramen on *G. infernalis* TNHC 18988 (Fig. 27B) and left internal carotid foramen in *G. infernalis* TxVP M- 13442 and *G. infernalis* TxVP M- 13441 are oriented somewhat anteromedially. In *G. infernalis* TxVP M- 13440, the left anterior opening for the internal carotid foramen is much larger than the right opening. In other species of *Gerrhonotus* the internal carotid foramina face anteromedially.

AG. Anterior extent of the basipterygoid processes of the sphenoid relative to the main body of the bone (new feature).

In several specimens of *Gerrhonotus* and *Elgaria*, the basipterygoid processes extend far anteriorly (e.g., *E*. *coerulea* TNHC 58792 and *G. parvus* SRSU 5538) (Fig. 27C) compared to other specimens (Fig. 27E). We observed a continuous range of variation in the anterior extent of the basipterygoid processes among specimens and we choose not to score this feature in discrete qualitative states. Some specimens that have basipterygoid processes that extend relatively far anteriorly (e.g., *G. parvus* SRSU 5538) also have a sphenoid that is somewhat wider in anterior view (Fig. 27D) suggesting a correlation between the two features. However, in *E. multicarinata* TxVP M- 8990 and *E*. *coerulea* (TxVP M- 9008, TxVP M- 8965) the basipterygoid processes extend far anteriorly, but the sphenoid does not appear wide in anterior view. In one juvenile specimen (*E. multicarinata* TxVP M- 8982), the basipterygoid processes extend far anteriorly and the sphenoid is not widened in anterior view, but in another juvenile specimen (*E. multicarinata* TxVP M- 8578), the basipterygoid processes do not extend far anteriorly but the sphenoid is relatively wide in anterior view. The shape of the sphenoid was shown to vary ontogenetically in *Shinisaurus* (Bever, Bell & Maisano, 2005). An increased sample of juvenile gerrhonotines is necessary to shed further light on patterns of ontogenetic variation in sphenoid morphology.

### Prootic

**Figure 29:**
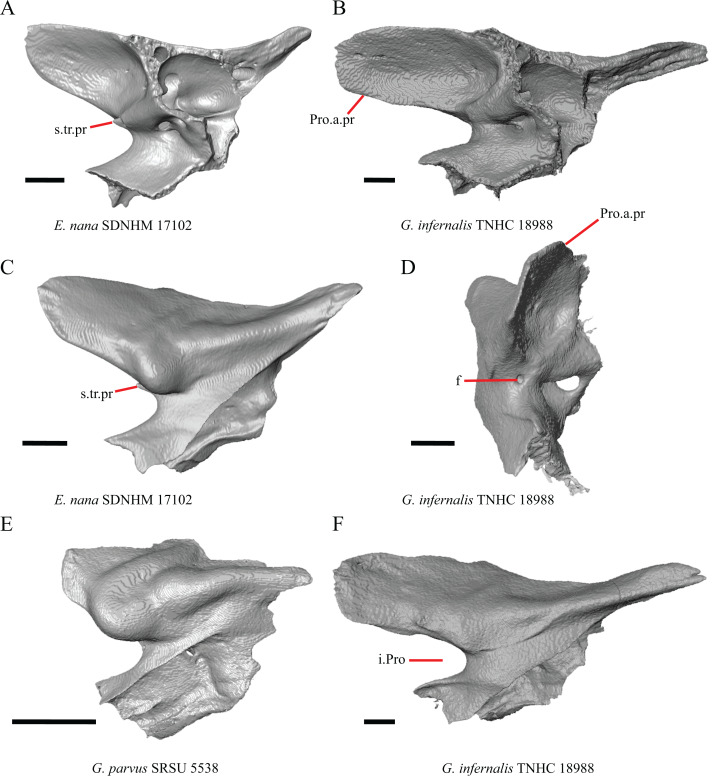
Prootics of some species of *Elgaria* and *Gerrhonotus*. (A) Prootic of *E. nana* SDNHM 17102 in medial view. (B) Prootic of *G. infernalis* TNHC 18988 in medial view. (C) Prootic of *E. nana* SDNHM 17102 in lateral view. (D) Prootic of *G. infernalis* TNHC 18988 in anterior view. (E) Prootic of *G. parvus* SRSU 5538 in lateral view. (F) Prootic of *G. infernalis* TNHC 18988 in lateral view. All scale bars equal 1 mm. f, foramen; i.Pro, incisura prootica; Pro.a.pr, prootic alar process; s.tr.pr, supratrigeminal process.

46. Supratrigeminal process on the prootic: 0=absent, Fig. 29B; 1=present, Figs. 29A (Estes, de Queiroz & Gauthier, 1988, character 50; Evans, 2008).

The supratrigeminal process was reported to divide the incisura prootica in *Gerrhonotus* and some *Elgaria* (Evans, 2008). We found that a supratrigeminal process is present on all *Elgaria* except for *E. multicarinata* TxVP M- 8980 and the right prootic of *E. multicarinata* TNHC 35666. The process is not visible in lateral view in many specimens. A small supratrigeminal process is present in specimens of *G. parvus*, *G. lugoi* LACM 116254, and *G. liocephalus* TCWC 8585. The process is absent in *G. ophiurus* and *G. infernalis* except for the presence of a small supratrigeminal process in *G. infernalis* TxVP M- 7129. Additionally, on the right prootic of *G. infernalis* TNHC 18988 and on both sides of *G. liocephalus* TCWC 9896, a foramen is present in the same location where a supratrigeminal process would be (Fig. 29D).

**Figure 30:**
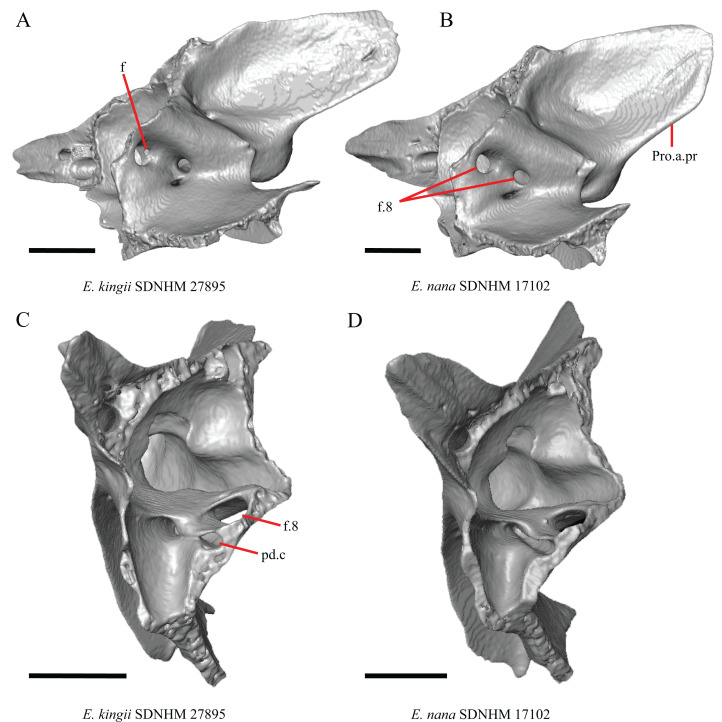
Prootics of some species of *Elgaria*. (A) ****Prootic of *E. kingii* SDNHM 27895 in ventromedial view. (B) Prootic of *E. nana* SDNHM 17102 in ventromedial view. (C) Prootic of *E. kingii* SDNHM 27895 in posterior view. (D) Prootic of *E. nana* SDNHM 17102 in posterior view. All scale bars equal 1 mm. f, foramen; f.8, foramen for vestibulocochlear nerve; pd.c, canal for the perilymphatic duct; Pro.a.pr, prootic alar process.

47. Presence of an additional separate foramen in the acoustic recess: 0=absent, Fig. 30B; 1=present, Fig. 30A (new feature).

In many specimens of *Elgaria* there is a small foramen located near the posterior acoustic foramen that opens posteriorly into the cavum capsularis. This foramen is present on only one side in some specimens (e.g., *E. kingii* SDNHM 24252) and is not fully enclosed by bone in others (e.g., left prootic of *E. cedrosensis* SDNHM 30296). Two anterior acoustic foramina were reported in *Ctenosaura pectinata* (Oelrich, 1956). However, it is not clear whether the foramen observed in *Elgaria* represents a second anterior acoustic foramen, because it is located near to and may merge with the posterior acoustic foramen, as seen in *E. cedrosensis* SDNHM 30296.

48. Perilymphatic duct on the prootic: 0=closed, Fig. 30C; 1=open, Fig. 30D (new feature).

An enclosed canal for the perilymphatic duct was reported on the prootic of *E. panamintina* (Ledesma & Scarpetta, 2018). We found a canal for the perilymphatic duct in many other specimens as well, but the canal is not completely enclosed in all specimens (e.g., *E. nana* SDNHM 17102, and *E. multicarinata* TxVP M- 8992, TxVP M- 8991).

AH. Condition of the alar process of the prootic (Good, 1987).

Variation in the shape of the alar process was previously reported in gerrhonotines (Good, 1987). Our data corroborate those observations. Many specimens have long alar processes (e.g., *G. infernalis* TNHC 18988, Fig. 29F), but several specimens (e.g., *G. parvus*, Fig. 29E) have a relatively short alar process of the prootic. We observed continuous variation in the length of the alar process. The length of the alar process of the prootic was shown to vary through ontogeny in *E. multicarinata* (Bhullar, 2012) and in other anguimorphs (Bever, Bell & Maisano, 2005). One juvenile specimen (*E. multicarinata* TxVP M- 8578) also has a short alar process.

### Otooccipital

**Figure 31:**
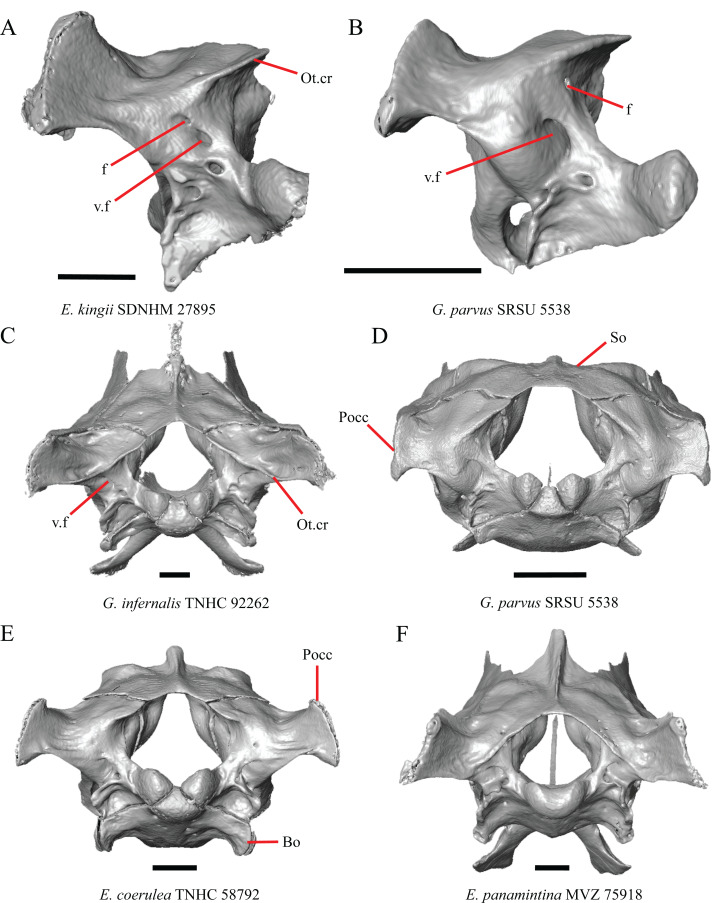
Otooccipitals and braincases of some species of *Elgaria* and *Gerrhonotus*. (A) ****Otooccipital of *E. kingii* SDNHM 27895 in posterior view. (B) Otooccipital of *G. parvus* SRSU 5538 in posterior view. (C) Braincase of *G. infernalis* TNHC 92262 in posterior view. (D) Braincase of *G. parvus* SRSU 5538 in posterior view. (E) Braincase of *E. coerulea* TNHC 58792 in posterior view. (F) Braincase of *E. panamintina* MVZ 75918 in posterior view. All scale bars equal 1 mm. Bo, basioccipital; f, foramen; Ot.cr, otooccipital crest; Pocc, paroccipital process; So, supraoccipital; v.f, vagus foramen.

49. Presence of a foramen dorsal to the vagus foramen: 0=absent, Fig. 31C; 1=present, Fig. 31A (new feature).

*Elgaria kingii* SDNHM 27895 and *G. parvus* SRSU 5538 (Fig. 31B) have the unusual condition of having a foramen on the left otooccipital that opens dorsal to the vagus foramen and empties into an enclosed hollow chamber in the otooccipital located medial to the posterior semicircular canal (Fig. 31A).

50. Extent of a crest on the posterior edge of the supraoccipital extending onto the posterior surface of the otooccipital: 0= crest does not extend to the ventral margin of the paroccipital processes, Fig. 31E; 1= crest reaches the ventral margin of the paroccipital processes, Fig. 31C (new feature).

There is variation in the length of a crest extending from the posterior edge of the supraoccipital onto the posterior surface of the otooccipital. This crest extends to the ventral edge of the paroccipital process in some specimens of *G*. *infernalis* (e.g., *G. infernalis* TNHC 18988, TNHC 92262). Among *Elgaria*, the crest is longest in *E. panamintina* MVZ 75918 (Fig. 31F) and some specimens of *E. multicarinata*. The crest is somewhat shorter in other specimens of *Elgaria* (e.g., *E*. *coerulea* TNHC 58792, Fig. 31E). There is a continuous spectrum of variation in the length of the crest in *Elgaria* which is likely a result of some degree of ontogenetic variation because juvenile specimens of *Elgaria* all have a short crest. Although *E. cedrosensis* SDNHM 30296 lacks a continuous crest running from the supraoccipital to the ventral margin of the paroccipital processes, that specimen does have a short crest near the ventral margin of the paroccipital processes that continuous as a small lateral projection.

AI. Length of the paroccipital processes (Bhullar, 2011).

The length of the paroccipital process is variable among specimens of *Elgaria* and *Gerrhonotus*. The paroccipital process is shortest in *G. parvus* SRSU 5538 (Fig. 31D) and longest in *E. paucicarinata* SDNHM 45106 and some specimens of *G. infernalis* (Fig. 31C). We chose to not score this feature as discrete qualitative states due to continuous variation in length.

### Dentary

**Figure 32:**
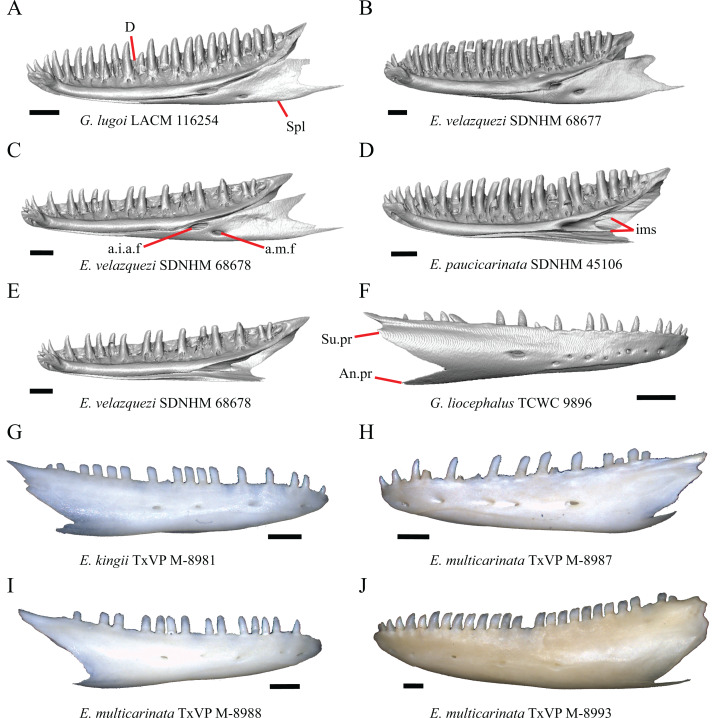
Dentaries of some species of *Elgaria* and *Gerrhonotus*. (A) Dentary and splenial of *G. lugoi* LACM 116254 in medial view. (B) Dentary and splenial of *E. velazquezi* SDNHM 68677 in medial view. (C) Dentary and splenial of *E. velazquezi* SDNHM 68678 in medial view. (D) Dentary of *E. paucicarinata* SDNHM 45106 in medial view. (E) Dentary of *E. velazquezi* SDNHM 68678 in medial view. (F) Dentary of *G. liocephalus* TCWC 9896 in lateral view. (G) ****Dentary of *E. kingii* TxVP M-8981 in lateral view. (H) Dentary of *E. multicarinata* TxVP M-8987 in lateral view. (I) Dentary of *E. multicarinata* TxVP M-8988 in lateral view. (J) Dentary of *E. multicarinata* TxVP M-8993 in lateral view. All scale bars equal 1 mm. a.i.a.f, anterior inferior alveolar foramen; a.m.f, anterior mylohyoid foramen; An.pr, angular process; D, dentary; ims, intramandibular septum; Spl, splenial; Su.pr, surangular process.

51. Contribution of the dentary to the anterior inferior alveolar foramen: 0=dentary does not contribute to the anterior and dorsal border, Fig. 32C; 1=dentary contributes to the dorsal and anterior border, Fig. 32A (Estes, 1964; Estes, de Queiroz & Gauthier, 1988; Conrad et al., 2011, character 183).

It was reported previously that the dentary contributes to both the dorsal and anterior bordering of the anterior inferior alveolar foramen in Anguidae (Estes, de Queiroz & Gauthier, 1988). However, it was also reported that in all gerrhonotines except for *Elgaria* the dentary contributes only to the dorsal margin of the anterior inferior alveolar foramen (Conrad et al., 2011). We found that in most specimens the dentary contributes to both the anterior and dorsal margin of the anterior inferior alveolar foramen. Interestingly, the dentary does not contribute to the anterior inferior alveolar foramen in *E. velazquezi* SDNHM 68678 (Fig. 32C), in some specimens of *E. kingii* (SDNHM 27895, on the left side of SDNHM 24252), and on the left side of *E. multicarinata* TxVP M- 8990 because the anterior inferior alveolar foramen is enclosed entirely within the splenial. In those specimens, the dentary is lacking a posterior-facing spine that usually forms the anterior and a small portion of the ventral margin of the anterior inferior alveolar foramen. An elongate projection of the splenial dorsal to the anterior inferior alveolar foramen excludes the dentary from contributing to the dorsal border of the foramen in *E. velazquezi* SDNHM 68677 (Fig. 32B), *E*. *coerulea* TNHC 58792, two specimens of *E. multicarinata* (TNHC 35666, left side of TxVP M- 8990), on the right side of *E. kingii* SDNHM 24252, and in *G. infernalis* TxVP M- 11411.

52. Number of tooth positions on the dentary (Good, 1987, character 95).

*Gerrhonotus* was described as unique compared to other gerrhonotine genera in having between 27-30 tooth positions on the dentary, compared to the 18–23 tooth positions reported for other genera (Good, 1987). We found that large specimens of *Elgaria* have 19–26 tooth positions on the dentary and specimens of *G. infernalis* have 25–28 tooth positions. Specimens of *G. parvus* and *G. lugoi* have 21–23 tooth positions and *G. liocephalus* and *G. ophiurus* have between 20 and 26 tooth positions.

53. Number of labial nutrient foramina on the dentary (Evans, 2008).

We found intraspecific variation and bilateral asymmetry in the number of nutrient foramina on the lateral surface of the dentary with specimens ranging from having four to nine foramina (*G. liocephalus* TCWC 9896, Fig. 32F).

54. Presence of two posteriorly oriented projections of the intramandibular septum: 0=absent, Fig. 32E; 1=present, Fig. 32D (new feature).

In *E. paucicarinata* SDNHM 45106 (Fig. 32D) and on the left dentaries of *E. paucicarinata* SDNHM 45100, *G. liocephalus* TCWC 8585, and *Gerrhonotus ophiurus* TCWC 35604 there are two posteriorly oriented projections of the intramandibular septum. This condition was not observed in any other specimens.

AJ. Condition of the surangular process on the dentary (Conrad et al., 2011, character 185).

*Elgaria* reportedly lacks a surangular process (identified as the coronoid process of the dentary in Ledesma & Scarpetta, 2018) on the dentary (Conrad et al., 2011). We found continuous variation in the distinctiveness of a surangular process in *Elgaria*, which made it difficult to easily separate into discrete states (Figs. 32F–32J). Specimens ranged from having no distinct surangular process, the condition in most specimens of *Elgaria* and *Gerrhonotus* (e.g., *E. multicarinata* TxVP M- 8988, Fig. 32I), to having a distinct posteriorly projecting surangular process (e.g., *E. velazquezi* SDNHM 68677, *E. multicarinata* TxVP M- 8987, Fig. 32H, *E. panamintina* MVZ 191076, and *G. liocephalus* TCWC 9896, Fig. 32F) with some specimens being bilaterally asymmetric (e.g., *E. panamintina* MVZ 191076 and *E. velazquezi* SDNHM 68677).

### Coronoid

**Figure 33:**
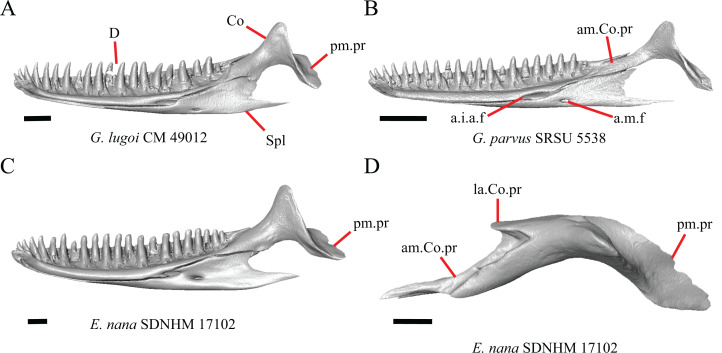
Dentaries, splenials, and coronoids of some species of *Elgaria* and *Gerrhonotus*. (A) Dentary, splenial, and coronoid of *G. lugoi* CM 49012 in medial view. (B) Dentary, splenial, and coronoid of *G. parvus* SRSU 5538 in medial view. (C) Dentary, splenial, and coronoid of *E. nana* SDNHM 17102 in medial view. (D) Coronoid of *E. nana* SDNHM 17102 in dorsal view. All scale bars equal 1 mm. a.i.a.f, anterior inferior alveolar foramen; am.Co.pr, anteromedial process of the coronoid; a.m.f, anterior mylohyoid foramen; D, dentary; Co, coronoid; la.Co.pr, lateral coronoid process; pm.pr, posteromedial process; Spl, splenial.

55. Extension of the visible portion of the anteromedial process of the coronoid relative to the last tooth position on the dentary when in articulation with the splenial; 0=anteromedial process is posterior relative to the last tooth position, Fig. 33B; 1=anteromedial process extends anterior to the last tooth position, Fig. 33C (modified from Good (1987), character 86).

*Elgaria* and *Gerrhonotus* reportedly possess an anteromedial process of the coronoid that projects anterior relative to the posterior margin of the posterior-most tooth position on the dentary (Good, 1987). We modified this character because the original description did not specify whether the character was scored for the entire anteromedial process of the coronoid or only the part visible when in articulation with the splenial. We inferred that the latter was more likely because the entire anteromedial process of the coronoid projects anteriorly much farther than the last tooth position of the dentary in all specimens. We found that the visible portion of the anteromedial process of the coronoid fails to extend anteriorly past the last tooth position on the dentary when in articulation with the splenial in *G. parvus* SRSU 5538 (Fig. 33B), *G. lugoi* CM 49012 (Fig. 33A), and the left side of *E. nana* SDNHM 52886. The condition in *G. parvus* SRSU 5537 cannot be determined due to the deteriorated condition of the bones.

56. Presence of a lateral process of the coronoid: 0=absent; 1=present, Fig. 33D (Conrad et al., 2011, character 193).

All gerrhonotines besides *Elgaria* were reported to lack a lateral process of the coronoid (coronoid labial flange of Conrad et al., 2011). We found that a lateral process is present in all specimens of *Elgaria* and *Gerrhonotus*.

**Figure 34:**
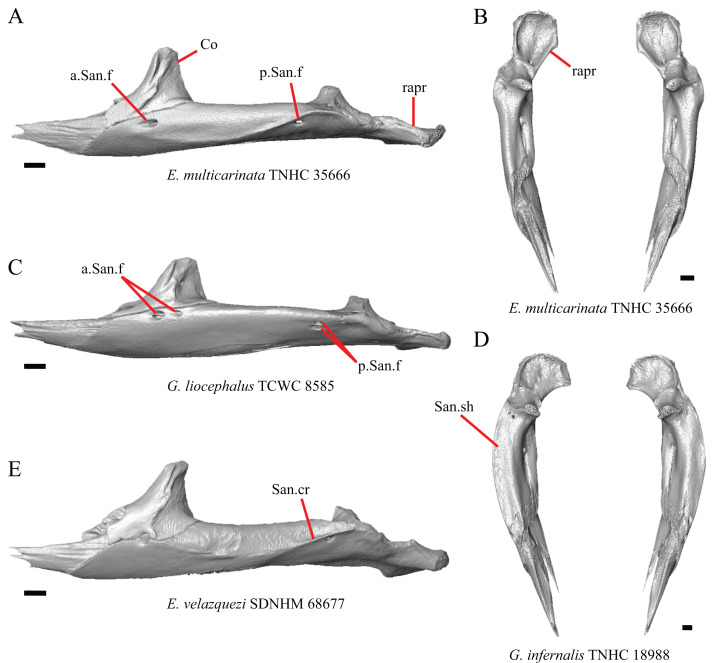
Coronoids and surangular and articular complexes of some species of *Elgaria* and *Gerrhonotus*. (A) Coronoid and surangular and articular complex ****of *E. multicarinata* TNHC 35666 in lateral view. (B) Coronoid and surangular and articular complex ****of *E. multicarinata* TNHC 35666 in dorsal view. (C) Coronoid and surangular and articular complex ****of *G. liocephalus* TCWC 8585 in lateral view. (D) Coronoid and surangular and articular complex ****of *G. infernalis* TNHC 18988 in dorsal view. (E) Coronoid and surangular and articular complex ****of *E. velazquezi* SDNHM 68677 in lateral view. All scale bars equal 1 mm. a.San.f, anterior surangular foramen; Co, coronoid; p.San.f, posterior surangular foramen; rapr, retroarticular process; San.cr, surangular crest; San.sh, surangular shelf.

57. Position of the coronoid relative to the border of the anterior surangular foramen: 0=coronoid is relatively far from the anterior surangular foramen, Fig. 34A; 1=coronoid is proximal to the anterior surangular foramen with no notch on the coronoid, Fig. 34C; 2=coronoid is proximal to the anterior surangular foramen with corresponding notch on the coronoid, Fig. 34E (modified from Conrad et al. (2011), character 173; Bhullar, 2011, character 205).

A coronoid contribution to the external border of the anterior surangular foramen was reported as an unambiguous synapomorphy of the least inclusive clade containing *Parophisaurus pawneensis*, *Paragerrhonotus ricardensis*, Gerrhonotinae, and Glyptosaurinae clade (Conrad et al., 2011). It was difficult to determine what constituted a contribution to the anterior surangular foramen, so we modified the states to describe the relative position of the coronoid to the anterior surangular foramen. We found that the anterior surangular foramen is located proximal to the coronoid in all specimens except for *E. multicarinata* TNHC 35666. Additionally, we found that the coronoid possesses a distinct notch corresponding to the dorsal border of the anterior surangular foramen on one or both sides in many specimens of *Elgaria* and some *Gerrhonotus* (e.g., *E. velazquezi* SDNHM 68677).

AK. Angle of the posteromedial coronoid process with respect to the horizontal axis of the mandible (Good, 1987, character 84).

**Figure 35:**
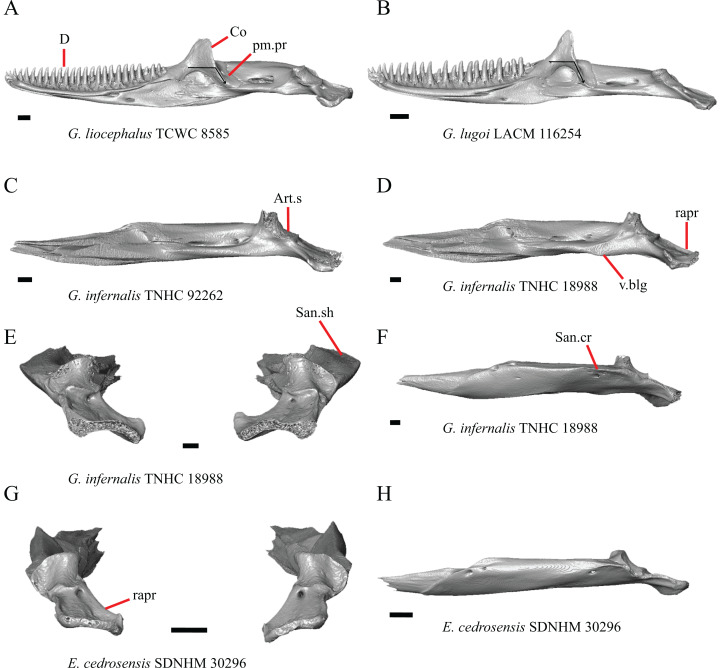
Mandibles and surangular and articular complexes of some species of *Elgaria* and *Gerrhonotus*. (A) ****Mandible of *G. liocephalus* TCWC 8585 in medial view. (B) Mandible of *G. lugoi* LACM 116254 in medial view. (C) Surangular and articular complex of *G. infernalis* TNHC 92262 in medial view. (D) Surangular and articular complex of *G. infernalis* TNHC 18988 in medial view. (E) Surangular and articular complex of *G. infernalis* TNHC 18988 in posterior view. (F) Surangular and articular complex of *G. infernalis* TNHC 18988 in medial view. (G) Surangular and articular complex of *E. cedrosensis* SDNHM 30296 in posterior view. (H) Surangular and articular complex of *E. cedrosensis* SDNHM 30296 in lateral view. All scale bars equal 1 mm. Art.s, articular surface for the quadrate; Co, coronoid; D, dentary; pm.pr, posteromedial process; rapr, retroarticular process; San.cr, surangular crest; San.sh, surangular shelf; v.blg, ventral bulge. Black arrows indicate the orientation of the coronoid posteromedial process.

A more ventrally directed posteromedial coronoid process (posteroventral process of Good, 1987) was reported in *Gerrhonotus* (Good, 1987). The orientation of the posteromedial coronoid process in specimens varies on a continuous spectrum, but the posteromedial coronoid process is oriented most ventrally in *G. lugoi* LACM 116254 (Fig. 35B). Some specimens of *Elgaria* also have a posteromedial coronoid process that is oriented somewhat ventrally (e.g., the right coronoid of *E. multicarinata* TxVP M- 9005). All other specimens of *Gerrhonotus* more closely resembled the condition typical of *Elgaria* in having a more posteriorly-facing posteromedial coronoid process (Fig. 35A).

### Surangular and articular complex

58. Ventral bulging of the prearticular: 0=absent, Fig. 35C; 1=present, Fig. 35D (Good, 1987, character 80).

*Gerrhonotus* was reported to have a ventral bulging on the prearticular anterior to the retroarticular process (Good, 1987). What we interpret as a ventral bulging is present in about half of the specimens of *G. infernalis* but not in other species of *Gerrhonotus*. *Elgaria multicarinata* TxVP M- 8993 also has a ventral bulging, which suggests that this feature may be correlated with larger size, since that specimen is the largest specimen of *Elgaria* that we examined. The notion that size is related to the presence of a ventral bulge is further supported by the fact that a similar ventral expansion was found in large individuals of *Lacerta viridis* (see figure 52 of Villa & Delfino, 2019).

59. Condition of the dorsal surface of the surangular shelf, anterior to the articular surface for the quadrate: 0=raised and curved, Fig. 35G; 1=relatively flat, Fig. 35E (similar to Good, 1987, character 82).

The dorsal surface of the surangular is relatively flat in most specimens of *G. infernalis* (Fig. 35E), *G. liocephalus* TCWC 9896, and *G. ophiurus* TCWC 35604. In *G. infernalis* (TxVP M- 13440, TxVP M- 13442) and on the left side of *G. liocephalus* TCWC 8585 the dorsal surface of the surangular is more raised and curved similar to that of *Elgaria*.

60. Fusion of the surangular and articular: 0=fused; 1=not fused (Conrad et al., 2011, character 166).

Lack of fusion between the surangular and articular (including the prearticular) was reported as an unambiguous synapomorphy of anguines, anniellines, gerrhonotines, and glyptosaurines (Conrad et al., 2011), but one researcher reported fusion of the articular and surangular in some gerrhonotines (Criley, 1968). Other authors noted that the bones are fused in most anguid genera (McDowell & Bogert, 1954) or reported that they were not fused (Rieppel, 1980). The surangular and articular are unfused in several specimens of *Elgaria* and *Gerrhonotus*. Juvenile specimens of *Elgaria* and some smaller specimens of *Gerrhonotus* have an unfused surangular and articular suggesting ontogenetic variation in the amount of fusion. This variation is consistent with previously reported intraspecific variation in fusion in *E. kingii* (Meszoely, 1970) and bilateral asymmetry of fusion in *E. multicarinata* (Evan, 2008). The articular and prearticular are fused in all specimens.

61. Number of anterior surangular foramina (modified from Conrad et al., 2011, character 172).

A distinct anterior surangular foramen was recovered as an unambiguous synapomorphy of *Elgaria* and was coded as absent in *G. liocephalus* (Conrad et al., 2011). We found that a distinct surangular foramen is present in all specimens of *Elgaria* and *Gerrhonotus*. In fact, there are two distinct anterior surangular foramina on one or both surangulars in several specimens of *Elgaria* and *Gerrhonotus* (e.g., *G. liocephalus* TCWC 8585, Fig. 34C). The presence of two anterior surangular foramina that pierce the coronoid was reported previously in *Xenosaurus platyceps* (Bhullar, 2011).

62. Number of posterior surangular foramina on the surangular: 0=single foramen, Fig. 34A; 1=two foramina, Fig. 34C (new feature).

*Gerrhonotus liocephalus* TCWC 8585 is unique in having two posterior surangular foramina on the left surangular (Fig. 34C).

AL. Width of the surangular shelf anterior to the articular surface (Good, 1987, character 82).

*Gerrhonotus* was reported to have a relatively broader and overall, more robust surangular (Good, 1987). We observed that the surangular in specimens of *G. infernalis* and *G. ophiurus* TCWC 3560 was broadest among specimens we examined; that may be related to the fact that in those specimens the surangular crest runs anteroposteriorly along the dorsolateral edge of the bone (e.g., *G. infernalis* TNHC 18988, Figs. 34D and 35F). Other specimens of *Gerrhonotus* do not appear as broad laterally and also do not have as distinct a crest along the dorsolateral edge of the bone (Fig. 34C). Specimens of *Elgaria* have a somewhat less broad surangular and have a surangular crest that either slants ventrally along the anterior portion of the bone or becomes indistinct anteriorly (e.g., *E. cedrosensis* SDNHM 30296, Fig. 35H). The width of the surangular shelf and distinctiveness of the surangular crest vary continuously among specimens.

AM. “…expansion of the dorsal edge at the posterior end of the surangular (just above the condylar facet)…” (Good, 1987:292, character 83).

An expansion on the dorsal surface of the surangular was reported to occur in all gerrhonotines except for *Gerrhonotus* (Good, 1987). We were unable to identify the feature referenced in this character, partly because our specimens did not differ in that way at that region of the surangular.

AN. Curvature of the posterior end of the surangular and articular complex (new feature).

The surangular and articular complex, including the retroarticular process, tends to be most strongly curved in *G. infernalis* (Fig. 34D) relative to other species (Fig. 34B); however, specimens vary along a continuous spectrum.

### Splenial

AO. Position of the anterior inferior alveolar foramen relative to the anterior mylohyoid foramen (Good, 1987, character 89).

It was reported that the anterior inferior alveolar foramen was farther from the anterior mylohyoid foramen in *Gerrhonotus* relative to other gerrhonotines (Good, 1987). When we looked at specimens of *Elgaria* and *Gerrhonotus* we did not observe clear qualitative differences in the distance between the foramina in these genera (Figs. 32A–32C and 33A–33C) and observed continuous variation in this feature.

### Osteoderms

**Figure 36:**
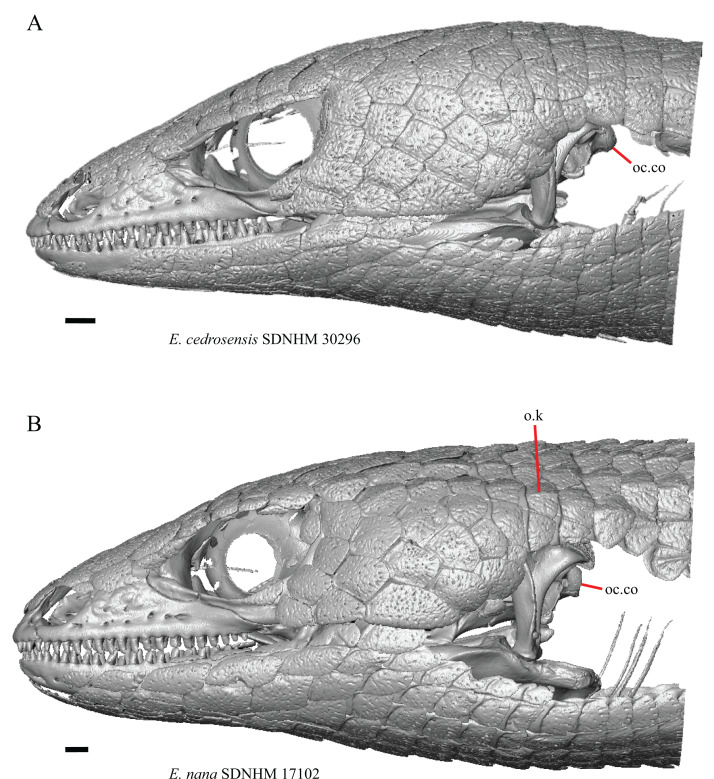
Skulls and osteoderms of some species of *Elgaria*. (A) Skull and osteoderms of *E. cedrosensis* SDNHM 30296 in lateral view. (B) Skull and osteoderms of *E. nana* SDNHM 17102 in lateral view. All scale bars equal 1 mm. oc.co, occipital condyle; o.k, osteoderm keel.

63. Condition of cranial dorsal osteoderms anterior to the occipital condyle: 0=no keeled osteoderms, Fig. 36A; 1=some osteoderms keeled, Fig. 36B (new feature).

Only *E. nana* and *E. multicarinata* have keeled cranial osteoderms (Fig. 36B).

## Discussion

**Figure 37:**
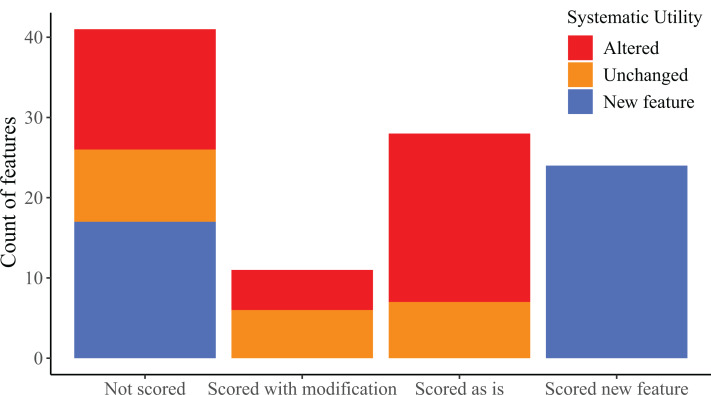
Summary of scoring and systematic utility of morphological features in this study.

We evaluated a total of 104 cranial features, including 38 features not previously discussed in gerrhonotines that we found to vary within our sample of specimens (Fig. 37). We discovered substantial variation in previously described osteological features. Much of that variation was previously undocumented, which speaks to the need for more in-depth investigations into osteological variation in squamate clades. Furthermore, we found that most purported systematically informative skeletal features for *Elgaria* and *Gerrhonotus* are subject to intra- and interspecific variation, which alters the diagnostic utility of those features.

For many features, specimens varied on a continuous spectrum. This made it difficult to discretize that variation into meaningful and/or objective categories, especially qualitative categories. For features originally described with states involving the presence or absence of some morphology, larger sample sizes sometimes revealed many intermediate variations of the ‘presence’ of a given structure which made it especially difficult to discretize features into objective categories (e.g., features F, G, and Q). For several features that vary on a continuous spectrum (e.g., features A, F, and I), future investigations using linear or geometric morphometric techniques (e.g., Rej & Mead, 2017; Gray et al., 2017) may provide a more objective means of evaluating those features quantitatively.

We also found that for some features, the orientation of skeletal elements influences scorings (e.g., features 33 and D). It is therefore imperative for researchers to be cognizant of how scorings may be impacted by the way they are viewing a feature. Furthermore, we discovered that for some previously described features it was unspecified whether the feature was originally scored on an isolated element or in articulation with multiple elements (e.g., feature 55). Additionally, we were unable to confidently identify some previously described features (e.g., features AC and AM). Our findings emphasize the need for researchers to effectively describe and communicate the way they are conceptualizing a feature in their descriptions. Explicit figures illustrating the described feature are essential in this regard; a distinct advantage of CT scanning is that the digital models can readily be manipulated for orientation, cross-sectional anatomy, or other desired aspects that greatly facilitate the construction of informative figures.

Several features that were used as characters in phylogenetic analyses of squamate or anguimorph relationships (e.g., features 15, 23, 41, C, D, E, I) and for which a given character state was reported to be an apomorphy of Gerrhonotinae, *Elgaria*, or *Gerrhonotus* have decreased utility when a larger sample of specimens and taxa are examined. That is because character matrices framed for eliciting higher-level relationships are generally different from those used to infer lower-level relationships (e.g., Augé & Guével, 2018; Marsh et al., 2019) and because taxon sampling and number of specimens examined in large-scale analyses are not as dense at lower taxonomic levels (i.e., a single species per genus; Gauthier et al., 2012). We identified many new features that are potential apomorphies of different gerrhonotine taxa (see discussion below). The study of variation is a part of the primary process of morphological discovery, but investigations into morphological variation often only assess previously described features or only report on variation that is seen as being systematically informative. We found a substantial amount of intraspecific variation in our sampled gerrhonotine genera, including evidence for ontogenetic variation in many features (e.g., features 9, 17, 18, 26, 52, 58, 60, C, I, M, O, W, AE, AG, AH, and AI). Our results demonstrate that continued study of many different types of variation at lower taxonomic resolution is valuable for understanding broader patterns of morphological variation to inform systematics and fossil identifications.

For our study we made observations on articulated and disarticulated dry skeletal specimens as well as CT-scanned alcohol-preserved specimens. CT data facilitated a unique opportunity to examine both articulated and disarticulated cranial elements on a single specimen, which allowed us to make exceptionally detailed observations of morphological variation. The use of CT allows us to report many morphological features that were not previously discussed in gerrhonotines (e.g., features 5, 11, 12, 18, 19, 22, 27, 29, 36, 37, 41, 43, 44, 45, 50, F, G, H, L, Q, R, V, W, X, and AA), and some features that were not documented or were poorly discussed in squamates in general (e.g., features 42, 43, 47, and 48). Many of these features would previously have been impossible or difficult to access on dry skeletal specimens, especially articulated specimens.

In a previous study of gerrhonotine cranial osteology Good (1987) reported three features that diagnose *Elgaria* (see features 25, 31, and 33) and eight features that diagnose *Gerrhonotus* (see features 52, 58, 59, A, Z, AD, AF, and AK). An analysis of anguimorph relationships (Conrad et al., 2011) listed five cranial features that were purportedly unambiguous synapomorphies of *Elgaria* (see features 26, 62, D, E, and AJ). We found that osteological variation in our sample altered the utility of almost all of those previously reported features. We sampled all species of *Elgaria* and five species of *Gerrhonotus*. Although we sampled at least two specimens of each species, except for *G. ophiurus*, to account for some measure of intraspecific variation, future investigations with increased sample sizes will almost certainly reveal additional sources of variation. Based on our current sample we found that no one particular cranial element could be used to identify a particular species of *Elgaria*; however, a few potential autapomorphies on some elements exist for a few species of *Gerrhonotus*. We found few clear differences useful to differentiate between *Elgaria* and *Gerrhonotus* and none were unambiguous. Many differences between species of *Elgaria* and between species of *Gerrhonotus* are subject to intraspecific variation reducing their utility in unambiguously differentiating taxa. Here we present a preliminary assessment of notable osteological differences, including differences subject to relatively smaller amounts of intraspecific variation, that may be useful to differentiate species of *Elgaria* and *Gerrhonotus*.

### Differences between *Elgaria* and *Gerrhonotus* present in at least 60 percent of specimens for each genus

1. Most *Elgaria* (except for some specimens of *E. kingii* and *E. multicarinata*) lack an ossified bridge on the premaxilla that encloses the medial ethmoidal foramen. All *Gerrhonotus* except for *G. parvus* have an ossified bridge on one or both sides of the premaxilla (Feature 2).
2. Most species of *Elgaria* have a midline foramen or foramina on the anterior surface of the alveolar plate of the premaxilla, except for *E. panamintina*, *E. cedrosensis*, and a single specimen of *E. kingii*. All species of *Gerrhonotus*, except for two specimens of *G. infernalis* lack a midline foramen on the anterior surface of the alveolar plate (Feature 3).
3. All *Elgaria* lack contact between the maxilla and frontal, but specimens of *G. infernalis, G. ophiurus*, and *G. lugoi* have the maxilla and the frontal in contact (Feature 6).
4. Almost all *Elgaria* have a distinct supratrigeminal process on the prootic while most *Gerrhonotus* do not have a supratrigeminal process (Feature 46).
5. All *Elgaria* have a raised and curved dorsal surface of the surangular shelf anterior to the articular surface for the quadrate, while most *G. infernalis*, *G. liocephalus*, and *G. ophiurus* have a flat dorsal surface of the surangular shelf (Feature 59).

### Differences between *Egaria* and *Gerrhonotus* not scored in discrete states due to continuous variation

1. Many specimens of *G. infernalis*, *G. liocephalus*, as well as *G. ophiurus* TCWC 35604 have a deeply excavated notch on the posterior edge of the facial process of the maxilla near the lacrimal articulation; however, a similar but smaller notch is also present in several specimens of *Elgaria* (Feature G).
2. The medially projecting lappet on the maxilla is often relatively short in species of *Gerrhonotus* and is often comparatively longer in species of *Elgaria* (Feature H).
3. Specimens of *G. infernalis* and some *G. lugoi* have a propensity to have the posterolateral border of the vomeronasal concavity of the vomer be more shallowly inclined with a less distinct separation from the nasal region, while specimens of *Elgaria* generally have a steeper and more distinct ridge that separates the nasal and vomeronasal regions of the vomer (Feature AA).

### Inter- and intraspecific variation among *Elgaria*

1. Most specimens of *Elgaria* have a spur on the anterior edge of the facial process of the maxilla, except for *E. cedrosensis*, most *E. kingii*, some *E. multicarinata*, some *E. coerulea*, and one *E. velazquezi* (Feature 12).
2. *Elgaria nana* and *E. velazquezi* lack a notch on the posterior edge of the parietal, but that morphology is also seen in some specimens of *E. panamintina*, *E. multicarinata*, and *E. coerulea* (Feature 16).
3. Bilateral concave recesses on the posterior surface of the parietal are present on *E. cedrosensis*, and on some specimens of *E. velazquezi*, *E. coerulea*, and *E. multicarinata* (Feature 18).
4. *Elgaria cedrosensis* and *E. paucicarinata* have an anterior projection on the posteroventral process of the prefrontal; however, some specimens of *E. kingii*, *E. multicarinata*, and *E. velazquezi* also have this condition (Feature 19).
5. *Elgaria paucicarinata* lacks a dorsal projection on the medial shelf of the lacrimal; however, some *E. coerulea*, *E. kingii*, and *E. multicarinata* lack a projection as well (Feature 20).
6. *Elgaria panamintina* has a distinct medial expansion of the postorbital at the anterior end of the supratemporal fenestra; however, some specimens of *E. coerulea*, *E. kingii*, *E. multicarinata*, and *E. velazquezi* have this condition as well (Feature 27).
7. *Elgaria panamintina*, one specimen of *E. kingii* (which is bilaterally asymmetrical), and *E. paucicarinata* (bilaterally asymmetrical in one specimen) are the only *Elgaria* that have a ridge on the pterygoid beginning anterior to the fossa columella and running along the lateral edge of the palatal plate to the ectopterygoid facet on the pterygoid flange (Feature 29).
8. *Elgaria panamintina* has a bifurcated head of the orbitosphenoid, as do some specimens of *E. coerulea*, *E. kingii*, and *E. paucicarinata* (Feature 43).
9. *Elgaria paucicarinata* (bilaterally asymmetric in one specimen) is unique among *Elgaria* in having two posteriorly directed free projections of the intramandibular septum (Feature 54).
10. Only *E. nana* and *E multicarinata* have some keeled cranial dorsal osteoderms anterior to the anteroposterior level of the occipital condyle (Feature 63).

### Inter- and intraspecific variation among *Gerrhonotus*

1. *Gerrhonotus parvus, G. lugoi*, and one specimen of *G. infernalis* have contact between the premaxilla and frontal, while in *G. liocephalus*, *G. ophiurus*, and the other specimen of *G. infernalis* for which the character could be scored, the premaxilla and frontal do not contact (Feature 1).
2. A thin posterior extension of the ventral keel of the premaxilla is present only in *G. parvus* and *G. lugoi* (Feature 5).
3. *Gerrhonotus lugoi* lacks a distinct medial projection at the anterior end of the palatine facet on the palatine process of the maxilla (Feature 8).
4. *Gerrhonotus infernalis* can have up to 26 tooth positions on the maxilla, while other examined specimens of *Gerrhonotus* have up to 23 tooth positions (Feature 9).
5. The nasals are separated near their anterior-posterior midpoint only in specimens of *G. lugoi* (Feature 13).
6. The anteromedial projection of the nasal is relatively far from the anteromedial inflection of the premaxillary process of the maxilla in *G. lugoi* (Feature 14).
7. Most specimens of *G. infernalis* have a medial projection at the anterior end of the medial shelf of the lacrimal (Feature 22).
8. *Gerrhonotus lugoi* has a relatively short posterior process of the septomaxilla compared to other *Gerrhonotus* (Feature 32).
9. *Gerrhonotus parvus* has a relatively short lamina on the lateral edge of the posterior palatine process of the vomer compared to other *Gerrhonotus* (Feature 37).
10. In most specimens of *G. infernalis* the posterolateral tip is positioned level or nearly level to the posterior-most extent of the supraoccipital where the bone forms a part of the margin of the foramen magnum. In other species of *Gerrhonotus*, the posterolateral tip of the dorsal surface of the supraoccipital is positioned anterior to the posterior-most extent of the supraoccipital (Feature 44).
11. In most *G. infernalis* the anterior openings for the internal carotid foramen face anteriorly. In other species of *Gerrhonotus* the anterior openings for the internal carotid foramen face anteromedially (Feature 45).
12. Most specimens of *G. infernalis* have a crest on the posterior edge of the supraoccipital that reaches the ventral margin of the paroccipital processes (Feature 50).
13. The posterior end of the surangular and articular complex of *G. infernalis* has a propensity to have the strongest lateral curvature among our sample of *Gerrhonotus* (Feature AN).

### Taxonomic considerations

Continued taxonomic revisions, newly described species, and novel phylogenetic hypotheses based on molecular data (e.g., Leavitt et al., 2017; Zheng & Wiens, 2016) change interpretations and conceptualizations of known morphological features. Conversely, morphology is also useful for hypothesizing phylogenetic relationships and framing taxonomy. It is therefore valuable to assess whether variation in the skulls of *Elgaria* and *Gerrhonotus* provides support for phylogenetic hypotheses and taxonomy of those groups. *Elgaria multicarinata* recently was found to be paraphyletic with respect to *E. panamintina* (Leavitt et al., 2017). However, we found no consistent differences in the skulls of the northern and southern *E. multicarinata* lineages inferred by Leavitt et al. (2017). We also found no consistent differences between *E. nana* and *E. multicarinata* and found one morphological feature (feature 63) shared between those two species to the exclusion of all other species of *Elgaria*.

The monophyly of *Gerrhonotus* including *G. lugoi* and *G. parvus* is currently undetermined (García-Vázquez et al., 2018a). We found several features shared by *G. lugoi* and other species of *Gerrhonotus* (e.g., features 2, 6, and 7). However, some of those features are present in other gerrhonotine genera according to Good (1987) (e.g., feature 2 and 6). Furthermore, we found several morphologies that, within our sample, are specific to *G. parvus* (e.g., feature 37), *G. lugoi* (e.g., features 8, 13, 14, and 32), or both species (feature 5). The phylogenetic position of *G. lugoi* is particularly interesting, because it was recovered in some analyses as being sister to *Barisia* (García-Vázquez et al., 2018a). We found that *G. lugoi* shares several features reported to occur in *Barisia*, *Mesaspis* (now synonymized with *Abronia*), or *Abronia* (Good, 1987), including a marked separation of the nasals from one another (although only near the midpoint of the nasals of *G. lugoi*) (feature 13) and the reduction of the length of the posterior process of the septomaxilla (feature 32).

## Conclusions

Our study represents the most exhaustive investigation into the cranial osteology of the gerrhonotine genera *Elgaria* and *Gerrhonotus*. We sampled all extant species of *Elgaria* and five of the nine species of *Gerrhonotus*. Most previously reported systematically informative skeletal features for *Elgaria* and *Gerrhonotus* are subject to intra- and interspecific variation, which alters their diagnostic utility. We report 38 new variable features for *Elgaria* and *Gerrhonotus* and present a preliminary assessment of osteological differences that may be useful to differentiate species and genera. Several cranial features may support phylogenetic hypotheses and taxonomy of *Elgaria* and *Gerrhonotus*. Much of the variation that we report in *Elgaria* and *Gerrhonotus* was previously undocumented including some features that were unknown or were poorly discussed in squamates in general. Our findings demonstrate that there is a need for more detailed investigations into patterns of morphological variation in squamate clades to facilitate an increased understanding of patterns of osteological variation for interpreting the fossil record, a conclusion that is broadly applicable across vertebrate clades.

The systematic utility of intraspecifically variable features was previously noted (Wiens, 1999), yet few authors have reported or emphasized such features. In part, that is because methods for integrating polymorphic characters into phylogenetic analyses are not straightforward. Nevertheless, continued investigations into morphological variation have yielded new insights into phylogenetic relationships and morphological evolution of squamates (e.g., Bhullar, 2011; Čerňanský, Smith & Klembara, 2014; Díaz-Fernández, Quinteros & Lobo, 2017; Stilson, Bell & Mead, 2017; Hernández Morales et al., 2019). Investigations into other vertebrate clades including turtles (Joyce & Bell, 2004), frogs (Bever, 2005), birds (Kirchner-Smith, 2015), and mammals (Gould, 2001) have also shown that substantial amounts of previously unreported morphological variation exist, some of which alter the diagnostic utility of previously reported features. Data on morphological variation in vertebrate clades serves as the foundation for interpretation of the fossil record, especially for taxa deeper in time for which genetic data are not available. Continued investigations into morphological variation are needed to better understand patterns of variation, including but not limited to intra- and interspecific variation, ontogenetic variation, and sexual dimorphism. Documenting these patterns of variation will greatly advance our ability to interpret patterns of morphological variation in the fossil record and may provide useful insights for systematics (e.g., Olori & Bell, 2012; Bhullar, 2012).

We highlight the need for researchers to effectively describe and communicate the way in which they are conceptualizing morphological features. Clear morphological descriptions and guiding figures will greatly facilitate continued investigations into morphological variation. Lastly, we reaffirm that X-ray computed tomography provides a unique opportunity to examine both articulated and disarticulated elements of the same specimen and can facilitate novel insights into patterns of morphological variation.

## Supplemental Information

10.7717/peerj.11602/supp-1 Supplemental Information 1 Matrix of scorings for features that involved counting and features that were discretized into distinct states.

## Additional Information and Declarations

### Data Availability

The following information was supplied regarding data availability: A matrix that contains scorings for features that were counts and features that we discretized into distinct states is provided in the Supplemental File. The raw CT data for the image-processed skulls used in this study are available at MorphoSource: https://www.morphosource.org/projects/000344118. The specimens on MorphoSource are available at: *Elgaria coerulea* TNHC 58792 (10.17602/M2/M344495), *Elgaria coerulea* TNHC 14643 (10.17602/M2/M344492), *Gerrhonotus parvus* SRSU 5537 (10.17602/M2/M344487), *Gerrhonotus parvus* SRSU 5538 (10.17602/M2/M344485), *Gerrhonotus lugoi* LACM 116254 (10.17602/M2/M344479), *Elgaria velazquezi* SDNHM 68678 (10.17602/M2/M344478), *Elgaria paucicarinata* SDNHM 45106 (10.17602/M2/M344472), *Elgaria velazquezi* SDNHM 68677 (10.17602/M2/M344469), *Elgaria nana* SDNHM 52886 (10.17602/M2/M344464), *Elgaria paucicarinata* SDNHM 45100 (10.17602/M2/M344460), *Elgaria nana* SDNHM 17102 (10.17602/M2/M344456), *Elgaria kingii* SDNHM 27895 (10.17602/M2/M344338), *Elgaria kingii* SDNHM 24252 (10.17602/M2/M344334), *Elgaria cedrosensis* SDNHM 30296 (10.17602/M2/M344330), *Elgaria cedrosensis* SDNHM 27702 (10.17602/M2/M344325), *Gerrhonotus ophiurus* TCWC 35604 (10.17602/M2/M344317), *Gerrhonotus liocephalus* TCWC 8585 (10.17602/M2/M344313), *Gerrhonotus lugoi* CM 49012 (10.17602/M2/M344300), *Gerrhonotus liocephalus* TCWC 9896 (10.17602/M2/M344292), *Gerrhonotus infernalis* TNHC 92262 (10.17602/M2/M344283), *Gerrhonotus infernalis* TNHC 18988 (10.17602/M2/M344278), *Elgaria panamintina* MVZ 75918 (10.17602/M2/M44396), *Elgaria multicarinata* TNHC 35666 (10.17602/M2/M44368), *Elgaria multicarinata* TNHC 4478 (10.17602/M2/M44360), *Elgaria panamintina* MVZ 191076 (10.17602/M2/M38315).
